# The Hox cluster microRNA miR-615: a case study of intronic microRNA evolution

**DOI:** 10.1186/s13227-015-0027-1

**Published:** 2015-10-07

**Authors:** Shan Quah, Peter W. H. Holland

**Affiliations:** Department of Zoology, University of Oxford, South Parks Road, Oxford, OX1 3PS UK

**Keywords:** Homeobox, miRNA, Mammal, Hoxc5, Intron

## Abstract

**Background:**

Introns represent a potentially rich source of existing transcription for the evolution of novel microRNAs (miRNAs). Within the Hox gene clusters, a miRNA gene, *miR*-*615*, is located within the intron of the *Hoxc5* gene. This miRNA has a restricted phylogenetic distribution, providing an opportunity to examine the origin and evolution of a new miRNA within the intron of a developmentally-important homeobox gene.

**Results:**

Alignment and structural analyses show that the sequence is highly conserved across eutherian mammals and absent in non-mammalian tetrapods. Marsupials possess a similar sequence which we predict will not be efficiently processed as a miRNA. Our analyses suggest that transcription of *HOXC5* in humans is accompanied by expression of *miR*-*615* in all cases, but that the miRNA can also be transcribed independently of its host gene through the use of an intragenic promoter. We present scenarios for the evolution of *miR*-*615* through intronic exaptation, and speculate on the acquisition of independent transcriptional regulation. Target prediction and transcriptomic analyses suggest that the dominant product of *miR*-*615* is involved in the regulation of growth and a range of developmental processes.

**Conclusions:**

The *miR*-*615* gene evolved within the intron of *Hoxc5* in the ancestor of placental mammals. Using *miR*-*615* as a case study, we propose a model by which a functional miRNA can emerge within an intron gradually, by selection on secondary structure followed by evolution of an independent miRNA promoter. The location within a Hox gene intron is of particular interest as the miRNA is specific to placental mammals, is co-expressed with its host gene and may share complementary functions.

**Electronic supplementary material:**

The online version of this article (doi:10.1186/s13227-015-0027-1) contains supplementary material, which is available to authorized users.

## Background

Intronic miRNAs provide an opportunity to gain insight into de novo miRNA innovation and its contribution to the evolution of development. Pre-existing transcribed RNAs, particularly in the form of introns, are thought to be a major source from which new miRNAs evolve [1, 2]. Here we analyse the origin and evolution of *miR*-*615*, a mammalian intronic miRNA located within a Hox cluster. We collate, analyse and integrate data on the regulation, expression pattern and likely biological roles of *miR*-*615*, in concert with comparative analysis, and propose how the origin of this miRNA contributed to eutherian evolution and development.

Homeobox genes form a large, diverse superclass with a wide range of roles in metazoan developmental biology. Most encode transcription factors which exert regulation over the expression of other genes during embryogenesis, thereby having a direct influence on the resultant adult morphology [3]. Perhaps the best characterised of the homeobox genes are the Hox genes, whose primary roles are in the specification of anteroposterior patterning in the developing bilaterian embryo. Hox genes also have other important roles during animal morphogenesis such as patterning of the limbs [4], embryonic kidney [5, 6] and reproductive tract [7]. Metazoan Hox clusters contain miRNA genes of varying ages and evolutionary origin. The *miR*-*10* gene is conserved between arthropods and mammals and occurs in the same position in insect and vertebrate Hox clusters [8]. The *miR*-*196* and the bi-directionally transcribed *miR*-*iab*-*4* genes are chordate-specific [9] and arthropod-specific [10], respectively. Another Hox cluster miRNA gene, *miR*-*615*, is less well-characterised and has been reported to be restricted to either mammals or amniotes [11]. A detailed analysis of the phylogenetic distribution of *miR*-*615* is currently lacking. Unlike *miR*-*10*, *miR*-*196* and *miR*-*iab*-*4*, which are intergenic, *miR*-*615* is nested within an intron of the *Hoxc5* gene in mouse and human. This positioning facilitates the evolutionary analysis of *miR*-*615* because the highly conserved nature of Hox gene exons allows alignment and comparison of intronic regions.

As *miR*-*615* occurs in the same orientation as *Hoxc5*, it is likely to be co-transcribed with its host gene. Work on other miRNAs supports a general concept of co-expression of intronic miRNAs with their host transcripts [12, 13], as well as revealing complementary functions performed by an intronic miRNA and its host gene product [14], although there is also evidence for some intronic miRNAs also having specific promoters [12, 15, 16].

Using DNA sequence data, derived from the public domain and obtained through our own experiments, we investigate the phylogenetic distribution of *mir*-*615* across tetrapods. We perform structural predictions and evaluate minimum folding energies to evaluate whether candidate pre-miR-615 transcripts could be efficiently processed into mature miRNAs, and argue that this most likely occurs only in eutherian mammals. We also integrate RNA-seq data with chromatin modification and expressed sequence tag (EST) studies to argue for the existence of a promoter specific to *mir*-*615* located within the transcription unit of *Hoxc5*. Finally, we carry out target predictions for the dominant 3p product of miR-615 and compare these to expression domains to suggest likely in vivo functions of this miRNA, and how these contributed to the evolution and development of mammals.

## Methods

### Conservation analysis for *mir*-*615*

Eutherian *Hoxc5* intronic sequences, along with those from *Xenopus tropicalis* and *Anolis carolinensis* were obtained from the UCSC Genome Browser [17] and the Ensembl genome database [18]. The *Sarcophilus harrisii* sequence was obtained from Ensembl while *Macropus eugenii* sequence data were obtained from GenBank (accession: JN378720.1). Monotreme DNA was obtained from Stephen Donnellan in the South Australian Museum via the Oxford University Museum of Natural History (CITES registration number: GB026). Three species were sampled: Platypus (*Ornithorhynchus anatinus*), Short-beaked echidna (*Tachyglossus aculeatus*) and Long-beaked echidna (*Zaglossus bruijni*). Xenarthran samples were provided by Mads Bertelsen at the Copenhagen Zoo; xenarthran genomic DNA was obtained from sloth (*Choeloepus didactylus*) heart and anteater (*Myrmecophaga tridactyla*) liver by phenol–chloroform extraction. Data for American pika (*Ochotona princeps*), lesser Egyptian jerboa (*Jaculus jaculus*) and common shrew (*Sorex araneus*) were obtained by running BLASTn using the hsa-miR-615 sequence against draft genome assemblies for these species. Alignment was carried out using MAFFT [19].

Primer sequences used for amplification of all *Hoxc5* intronic sequences are: 5′-TTGGACTTAAGCATCACTTTCCCACCG-3′ and 5′-CCAGAGTCTGGTAGCGCGTGTAACTGG-3′. These were designed to bind a region in the flanking coding exons conserved between most tetrapods (Additional file 1: Supplement S1). The annealing temperature was 60 °C for anteater and 55 °C for sloth. PCR products were gel purified using the illustra GFX PCR and DNA Gel Band Purification Kit (GE Healthcare, 28-9034-71). The sloth sequence was cloned into the pGEM-T Easy Vector System (Promega) before sequencing; the anteater amplicon was directly sequenced following gel purification with the same primers used for amplification.

Structural criteria for the annotation of functional miR-615 are: stable hairpin structure with <−18 kcal/mol free energy, at least 18 paired bases on the main stem and the absence of large internal loops and bulges in mature regions. Minimum free energy and predicted secondary structure was calculated for each putative miR-615 sequence using RNAeval.

### Bioinformatics and target prediction

Data used to determine expression of *mir*-*615* and *HOXC5* in human cell lines were obtained from the ENCODE project and visualised on the UCSC Genome Browser (http://genome.ucsc.edu/ENCODE/).

A list of publicly released datasets used in bioinformatic analysis of *mir*-*615* and mRNA expression is provided in Additional file 2: Supplement S2. These data were obtained from the Gene Expression Omnibus [20] and the ENCODE project. In the microarray studies, a gene was determined to be expressed based on the ABS_CALL value (P = present, A = absent, if provided). If this was not provided, a cut-off *p* value of <0.05 was used to determine expression. Raw data for the transcriptome assemblies were quality checked using FastQC, followed by trimming of the first 15 bases using the fastqtrim.py script. Trimmed reads were assembled using Trinity and resultant contig FPKMs determined with RSEM. BLASTn with standard parameters was used to determine Hox gene expression in each transcriptome, with appropriate sequences from the corresponding species obtained from GenBank. Small RNAseq data were analysed through the miRDeep2 pipeline using the quantifier.pl script to map reads onto known human miRNAs. Handling of raw reads prior to mapping was carried out as described in Quah et al. [14]. Read counts for mouse developmental stages as presented by Chiang et al. [21] were obtained from miRBase [22].

Target prediction for hsa-miR-615 was run using the downloadable executable for the PITA prediction algorithm [23] on all human cDNAs downloaded from Ensembl BioMart (genome assembly: GRCh38.p2). Predicted targets were ranked by score and a cutoff score of −20 was applied. Documented functions for predicted targets were obtained from UniProtKB/Swiss-Prot (http://www.uniprot.org/ [24]). RPKM values for tissue-specific expression of each gene were obtained from the Human Protein Atlas [25], except for cerebellum where RPKM values were obtained from gene quantifications available through the ENCODE project (accession: ENCFF917ANZ).

### *mir*-*615* promoter prediction

Histone methylation, DNase I and CAGE data used to find the position of the *mir*-*615* promoter were obtained using ENCODE data tracks available through the UCSC Genome Browser (http://genome.ucsc.edu/ENCODE/). The EST data track used (‘Human ESTs Including Unspliced’) was generated at UCSC from publicly available EST sequencing data.

## Results

### A sequence with high similarity to *mir*-*615* is present in therian mammals

miR-615 is reported as being restricted to either mammals or amniotes [11] but current literature lacks a detailed analysis of its evolutionary history. We first sought to characterise its phylogenetic distribution to determine its most likely time of origin.

**Fig. 1:**
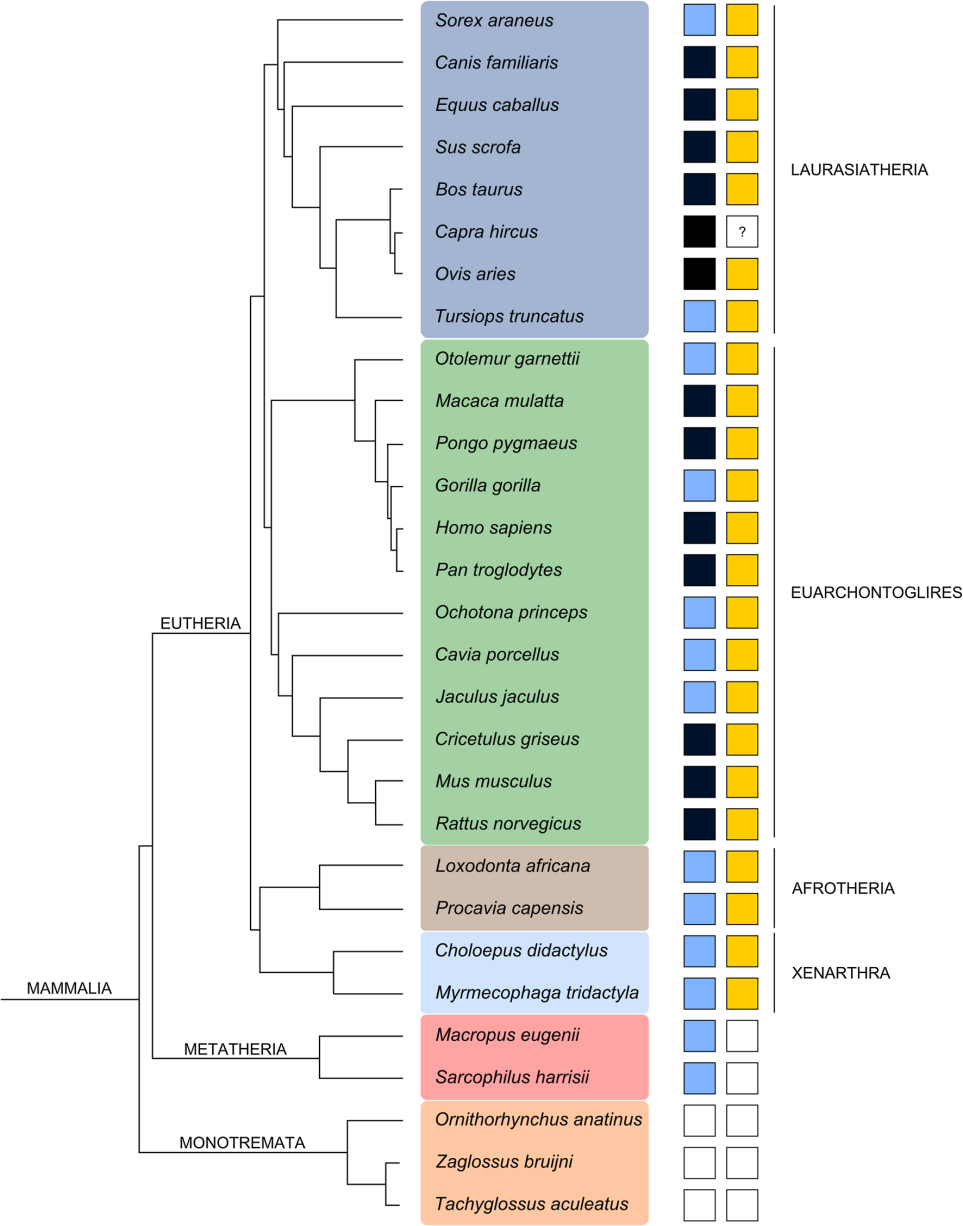
Phylogenetic distribution of *mir*-*615* in mammals. *Black squares* represent miRBase entries for miR-615 or published deep sequencing data. *Blue squares* indicate BLASTn predictions from genome data or PCR products obtained in this study. *Yellow squares* indicate strong secondary structure predictions

Available entries for *mir*-*615* on miRBase [22] are limited to two of the four main clades of eutherian mammals (Laurasiatheria and Euarchontoglires, Fig. 1). To determine whether miR-615 is present in other amniotes, we searched for its precursor gene sequence in the genomes of other eutherians, plus a marsupial (*Monodelphis domestica*), a monotreme (*Ornithorhynchus anatinus*) and three non-mammalian vertebrates (chicken *Gallus gallus*, anole lizard *Anolis carolinensis* and zebrafish *Danio rerio*). The non-eutherian species did not produce definitive matches, while a positive control BLASTn run using the same parameters successfully recovered *mir*-*615* in mouse (*Mus musculus*). Within eutherian mammals, we were able to identify the *mir*-*615* gene by BLASTn in the elephant (*Loxodonta africana*; Afrotheria), but not in the sloth (*Choloepus hoffmanni*; Xenarthra).

**Fig. 2:**
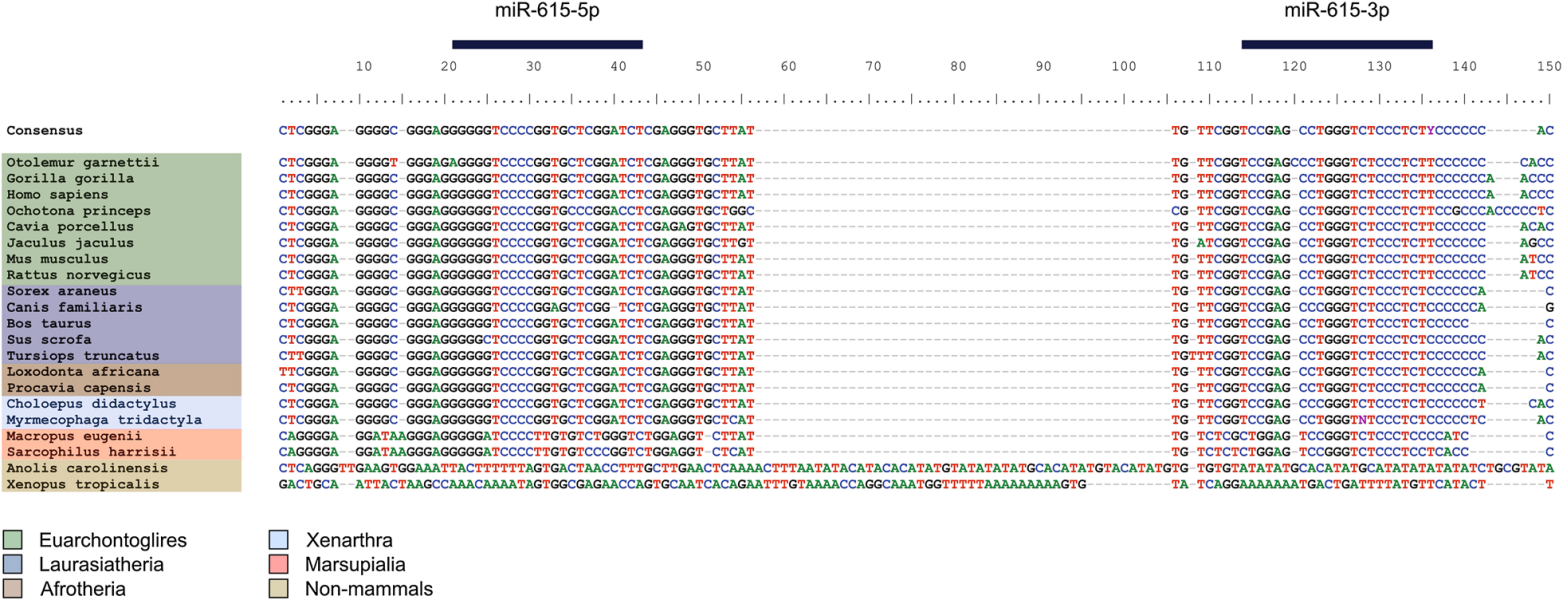
Part of a *Hoxc5* intronic alignment across tetrapods corresponding to the eutherian mir-615 region, extracted from an alignment of *Hoxc5* loci including exons. The complete alignment is provided in Additional file 3

The syntenic position of *mir*-*615* within a highly conserved Hox gene allows for direct comparisons of the corresponding region to be made between vertebrate species. In human and mouse, *mir*-*615* is located within the intron of *Hoxc5*. We performed alignments of this intron and its flanking coding exons across the four main eutherian lineages (Laurasiatheria, Euarchontoglires, Afrotheria, and Xenarthra), non-eutherian mammals and other tetrapods to examine sequence conservation across the syntenic position of *mir*-*615* (Fig. 2, Additional file 3: Supplement S3A). As no sequence data for *Hoxc5* were available for Xenarthra, we sequenced the corresponding region from sloth (*Choloepus didactylus*) and anteater (*Myrmecophaga tridactyla*) using primers designed against highly conserved regions within vertebrate *Hoxc5* coding regions (Additional file 1: Supplement S1). Sequence alignments reveal strong similarity to other eutherian *mir*-*615* loci (Fig. 2). In marsupials, both the Tasmanian devil (*Sarcophilus harrisii*) and the tammar wallaby (*Macropus eugenii*) possess a sequence resembling eutherian *mir*-*615* within *Hoxc5*. However, the regions corresponding to the mature miR-615 products differ slightly from the corresponding eutherian versions. We were unable to amplify the *Hoxc5* gene from any monotremes with the same primers. This genomic region is also missing in the platypus (*Ornithorhynchus anatinus*) genome assembly.

These alignment data suggest that the *mir*-*615* region is strongly conserved at the nucleotide sequence level within the Eutheria, and to a lesser extent in Metatheria, and confirm the lack of an orthologous sequence in monotremes and non-mammalian tetrapods (Fig. 1).

### Candidate miR-615 precursors fulfil structural and energetic criteria only in Eutheria

**Fig. 3:**
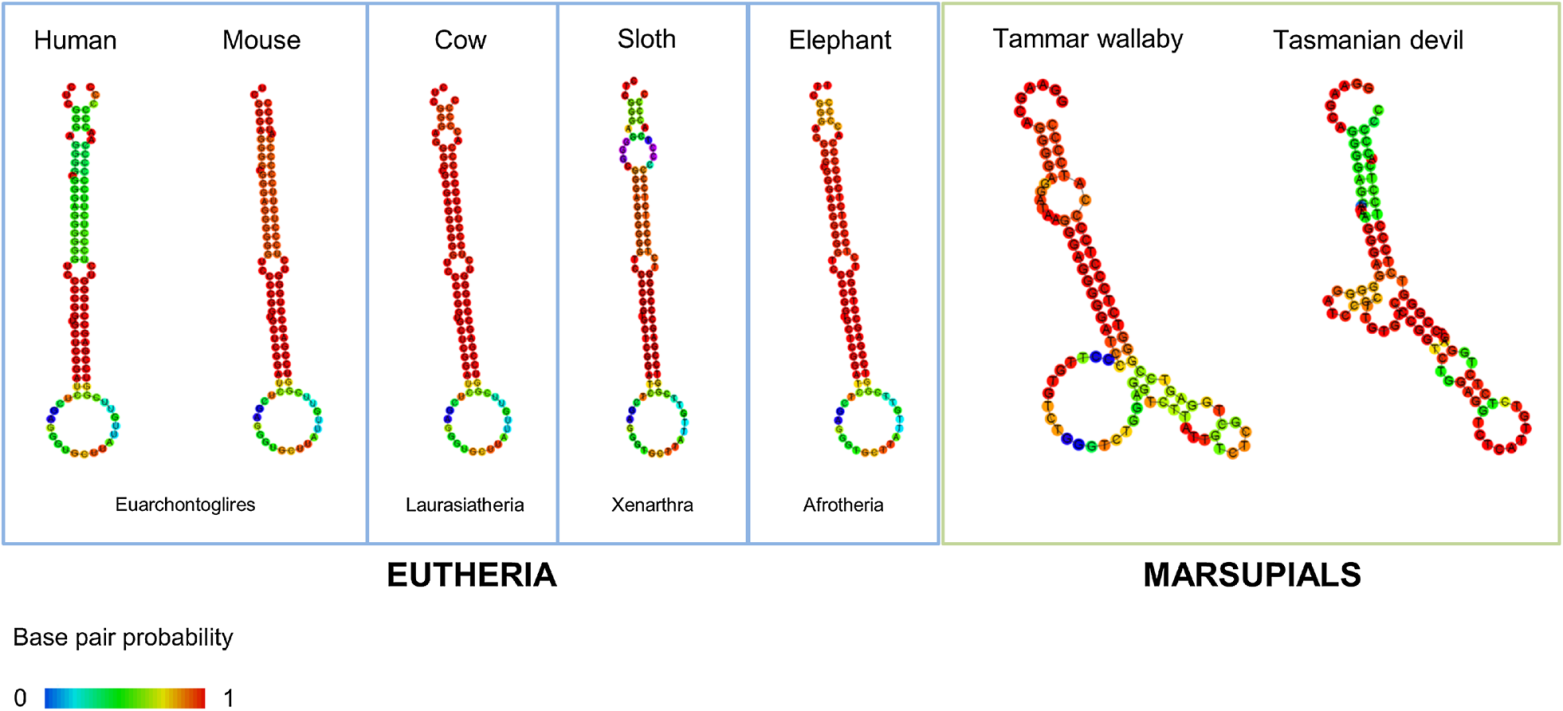
Predicted secondary structures for example miR-615 precursors in marsupials and the four major eutherian clades. Minimum free energies for all species tested are listed in Additional file 4

Having established that all therian mammals (eutherians and marsupials) studied possess a sequence similar to the annotated *mir*-*615* genes of human and mouse (Figs. 1, 2), we analysed these sequences for fulfilment of published structural and energetic criteria used in miRNA annotation. This analysis reveals that all the eutherian *mir*-*615* orthologues are likely to produce functional pre-miR-615. Analysis of aligned eutherian pre-miR-615 structures using RNAz is strongly supportive of the existence of a conserved fold (Additional file 3: Supplement S3B). However, in the two marsupials analysed minimum folding energy (MFE) for the putative pre-miR-615 homologues (−39.40 kcal/mol in *M. eugenii* and −42.60 kcal/mol in *S. harrisii*) are higher than those observed for all eutherian miR-615 precursors (mean MFE −59.40 kcal/mol). Both marsupial sequences also have large internal loops, bulges and mismatches which are likely to preclude efficient entry into the miRNA pathway (Fig. 3). These predicted structures also differ between *M. eugenii* and *S. harrisii*, suggesting a lack of selection on the secondary structure of this region in marsupials. It is therefore likely that marsupials lack the ability to generate mature miR-615.

### miR-615 expression in mammalian cell lines and adult tissues

Using small RNA deep sequencing data from the ENCODE project [26] obtained through the UCSC Genome Browser [17], we analysed miR-615 expression in 36 human cell lines and tissues (Additional file 5: Supplement S5). miR-615 is expressed in the majority of these datasets. A minority of cell lines lacked expression of miR-615, notably those with immune-related functions (CD14+, CD34+ and CD20+) as well as H1 embryonic stem cells, H1-neurons and HAoEC. There is no evidence for antisense transcription of miR-615 in any of the samples. In all cases where miR-615 is expressed, the miR-615-3p product is dominant.

Since the above browser included only ENCODE data released prior to 2013, we extended these analyses using available raw sequence reads from more recent small RNA deep sequencing projects available through the ENCODE Project Portal using the miRDeep2 quantifier module. We first confirmed that this approach gives comparable results to the UCSC browser information by analysing the raw sequence reads from the human K562 and GM12878 cell lines (Additional file 6: Supplement S6A). Using other deposited raw sequencing data sets, we then analysed the expression of miR-615 in five organs (cerebellum, cerebral cortex, heart, testis and kidney) in human and mouse [1], as well as ovary in goats [27] and sheep [28]. These data indicate that miR-615 is expressed in kidneys, cerebellum, testis and ovary (Additional file 6: Supplement S6B). Although miR-615 was not detected in whole mouse testis, it is present in the Sertoli cells and spermatocytes (see [1], Additional file 6: Supplement S6B). In the adult goat ovary, expression of both mature products of *mir*-*615* is increased during pregnancy (see [27], Additional file 6: Supplement S6C). Our analysis of existing small RNA sequencing data in the mouse (obtained from [21]) reveals that miR-615 expression is detected in whole mouse embryos (Additional file 7: Supplement S7). Expression of the miRNA is variable between replicates at each developmental stage, but displays a general trend of increase through embryogenesis and persistence in the newborn.

### Expression of miR-615 can occur independently from *Hoxc5*

Comparing the expression patterns of *mir*-*615* and those of *Hoxc5* would help test whether expression of *mir*-*615* is solely driven by its host transcription unit, or whether there is independent regulation of this intronic miRNA. Out of the 36 cell lines and tissue types used to survey miR-615 expression, 34 also had available RNA-seq data (Additional file 5: Supplement S5), which we analysed to study expression of *HOXC5*. We found evidence for simultaneous expression of both miR-615 and *HOXC5* in several datasets, such as the adipose-derived hMSC-AT mesenchymal stem cell line and NHDF human dermal fibroblasts. Some cell lines, such as H1 embryonic stem cells, express neither the miRNA nor its host gene. However, it is notable that in many of the cell lines where miR-615 is found, including the lymphoblastoid cell line GM12878 and the leukaemia-derived K562 cell line, there is little to no expression of *HOXC5*. To validate the observation that *HOXC5* is not expressed in K562 cells, we constructed a de novo transcriptome assembly for this cell line using raw data from the ENCODE Consortium [29] and again did not find evidence for *HOXC5* expression (Additional file 8: Supplement S8). There are no instances of *HOXC5* expression without concurrent expression of miR-615 in all the cell lines surveyed. Taken together, these data suggest that miR-615 is generated both from transcription of *HOXC5* and from its own promoter.

**Fig. 4:**
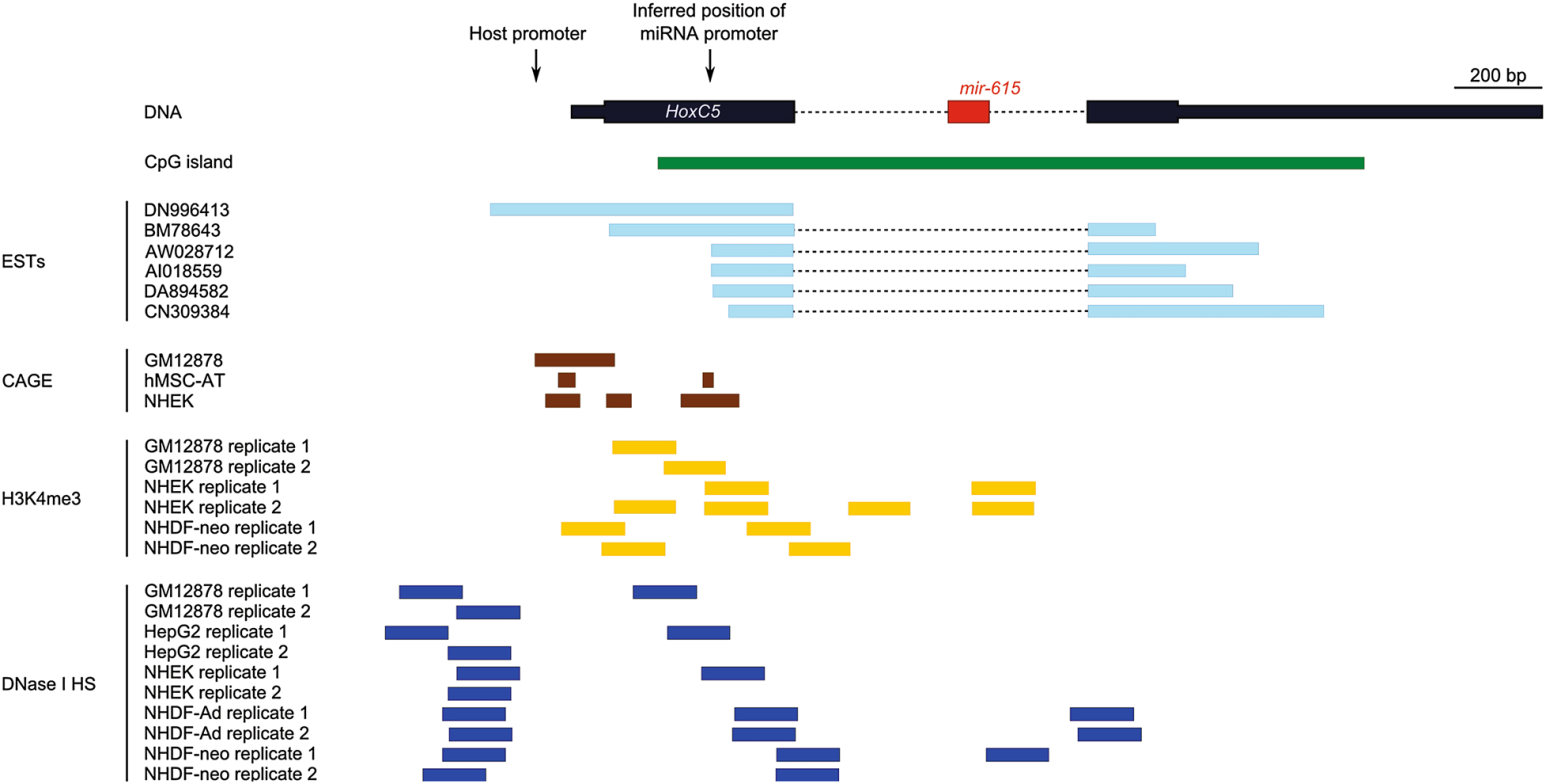
Inferred position of the mir-615 promoter based on EST, CAGE, H3K4 trimethylation and DNase I hypersensitivity data

Analysis of the reported chromatin landscape surrounding *mir*-*615* in ENCODE human cell lines provides evidence for the existence and location of a miRNA-specific promoter (Fig. 4). Trimethylation of histone H3 on lysine 4 (H3K4me3), a marker of active chromatin associated with transcriptional start sites (TSSs) is distributed throughout the first exon and the intron of *HOXC5* in cell lines that express *mir*-*615*. Cell lines which express neither *HOXC5* nor *mir*-*615*, represented by H1-hESC and AG044540, have minimal H3K4 trimethylation at the locus. DNase I hypersensitivity data also provides a measure of chromatin accessibility and is concentrated in two regions, one ~150 bp upstream of the annotated TSS for *HOXC5*, and the other distributed more broadly across the first exon and the 5′ end of the intron. A third region of DNase I hypersensitivity is also seen at the 3′ end of the intron, but only in NHDF cells.

The strongest indication of an independent promoter for *mir*-*615* comes from 5′ cap analysis of gene expression (CAGE) data, which identify potential initiation sites within the *HOXC5* locus through the detection of 5′ capped RNA transcripts. These sites map to both the known *HOXC5* TSS and a region about ~200 bp upstream of the exon–intron boundary, corresponding well with the 5′ ends of several expressed sequence tags (ESTs). This position also aligns with the DNase I hypersensitivity sites. Collectively, these analyses of chromatin accessibility and transcription at the *HOXC5* locus, coupled with evidence for expression of *mir*-*615* in the absence of *HOXC5*, strongly support of the existence of an internal miRNA-specific promoter, in addition to the *HOXC5* gene promoter.

### Candidate targets for miR-615-3p

Identifying likely targets for miR-615, integrated with the expression pattern data presented above, would contribute to our understanding of how this miRNA contributes to eutherian biology and evolution. Dominance of the 3p arm is observed in all cases where we have observed expression of *mir*-*615*, hence we undertook target prediction analysis for miR-615-3p against all known human cDNAs (Additional file 9: Supplement S9). This suggested that potential targets include transcripts encoding proteins with roles in development and function of the nervous system (e.g. KIF1A and PAX6), transcriptional regulation (e.g. YY1AP1), interaction with Hox proteins (PBX3) and cell cycle control (e.g. RSL1D1). For each target above the cut-off, we ascertained expression levels in tissues known to express miR-615 (kidney, testis, ovary and cerebellum). Some targets, such as *AES*, are co-expressed with *mir*-*615* in all tissues examined. Others are expressed with the miRNA in some tissues but not in others; *PAX6*, for example, is expressed in the cerebellum but not in the kidney or gonads. GO term distribution was compared between the 72 predicted targets and the input set of human cDNAs (Additional file 10: Supplement S10). GO terms upregulated in the target prediction dataset relative to all human cDNAs include those associated with growth and development, as well as those involved with signalling pathways (e.g. Notch) known to regulate development [30].

## Discussion

### Origins of eutherian *mir*-*615*

Sequence alignment and structural prediction suggests that *mir*-*615* generates functional mature products in all eutherian mammals. A similar sequence exists in the *Hoxc5* intron of marsupials and adopts a very approximate hairpin structure (Figs. 2, 3), but has not been identified as a miRNA gene [31]. This secondary structure, while not a completely random fold, does not conform to the energetically stable stem loop required for efficient entry into the miRNA processing pathway. We speculate that it is perhaps occasionally captured by the miRNA processing machinery, but is unlikely to be processed as efficiently as the eutherian precursor.

The presence of functional miR-615 in eutherians and its absence from marsupials could be explained by either the acquisition of *mir*-*615* on the lineage leading to the Eutheria following its divergence from marsupials, or through acquisition of the miRNA in a common therian ancestor followed by its lineage-specific loss in marsupials. While an approximation of the ancestral therian condition might be inferred through examination of the corresponding region in monotremes, we were unable to recover any convincing hits to *Hoxc5* by BLASTn against the *O. anatinus* (platypus) genome assembly, or amplify its intron through PCR in three monotreme species (platypus *O. anatinus*, short-beaked echidna *Tachyglossus aculeatus*, long-beaked echidna *Zaglossus bruijni*). It is plausible that monotremes have undergone lineage-specific loss of part of the *Hoxc* cluster; posterior *Hoxc* genes (*Hoxc10*–*Hoxc13*) are present in the platypus draft genome while *Hoxc4*, *Hoxc5*, *Hoxc6*, *Hoxc8* and *Hoxc9* are absent [32]. A hypothetical monotreme homolog of *miR*-*615* could therefore either have been lost with the *Hoxc5* gene, and we did not detect in any other genomic region. Given that that none of the 17 eutherian species studied across the four major eutherian lineages have lost this miRNA or significantly altered either of its mature products, we argue that *mir*-*615* is more likely to be a eutherian novelty rather than the alternative scenario where it arose on the common mammalian lineage and was lost in monotremes and marsupials.

### miR-615 as a model for understanding intronic miRNA evolution

Intronic exaptation has been proposed as a major contributor to miRNA innovation [1, 2]. Under this model, an intronic origin for a novel miRNA eliminates the need to evolve a separate promoter driving miRNA transcription, at least initially. This reduces the requirement for novel miRNA emergence to the acquisition of a suitable secondary structure by the existing transcript.

**Fig. 5:**
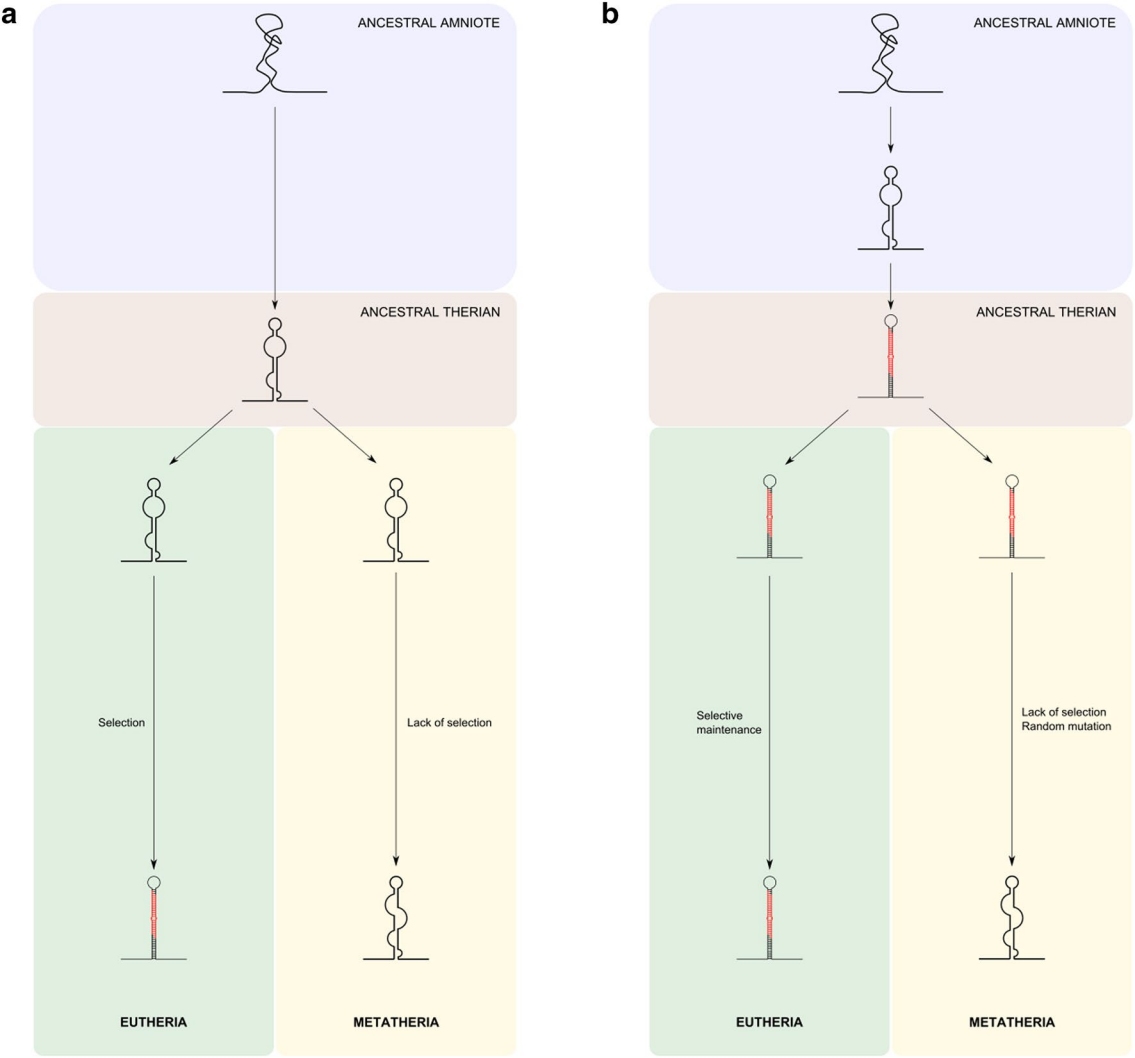
Hypothetical scenarios for the origin of miR-615. **a** Eutherian-specific origin. **b** Origin in the ancestral therian lineage followed by secondary loss in Metatheria

A possible model for the origin of miR-615, involving eutherian-specific origin via intronic exaptation, is shown in Fig. 5a. In this scenario, the ancestral sequence was not processed efficiently as a miRNA or stably integrated into any genetic regulatory pathways. Following the divergence of the Eutheria from the Metatheria, different evolutionary forces shaped the evolution of the intron in each lineage. Modification of the eutherian sequence, perhaps initially through drift and subsequently through positive selection on adaptive miRNA-target interactions, resulted in the emergence of a precise stem-loop structure which could efficiently enter the miRNA processing pathway [33]. The lack of either the stabilising mutations, or the lack of suitable selection pressure after the emergence of such a structure, would explain the absence of functional miR-615 in marsupials. The observed sequence-level similarity between the corresponding *Hoxc5* intronic regions in marsupials and eutherians, with a precise stem-loop structure present in eutherians but not marsupials, presents a strong case for the evolution of miR-615 through intronic exaptation.

Although we favour the above model as the most likely mode of origin of miR-615, we note that the predicted secondary structure of the homologous region in the *Hoxc5* intron of modern marsupials resembles an approximate hairpin more than a completely random fold (Fig. 3). This means we should at least consider the alternative possibility of secondary loss or degradation in the Metatheria (Fig. 5b). Terminal branches of phylogenies tend to be rich in young miRNAs, most of which undergo rapid turnover and will eventually be lost [1, 14, 34]. It is therefore not implausible that miR-615, newly emergent in the ancestral therian lineage, was captured by selection and retained in only in the eutherian lineage but not in Metatheria. Under this model, a young *mir*-*615* gene would not have been stably integrated into any genetic regulatory networks at the time of the eutherian-metatherian split. Positive selection on any beneficial miRNA-target interactions and drift represent two counteracting forces governing the fate of new miRNA genes in each lineage. In the Eutheria, the effect of positive selection in maintaining *mir*-*615* in the genome outweighed the degradation of the secondary structure through drift, while the opposite would have been true for the Metatheria. Such a model would push back the origin of *mir*-*615* to the common therian lineage.

Despite the finding that *Hoxc5* mRNA and *miR*-*615* are produced together, we also find evidence for regulation of *mir*-*615* independently from its host gene in modern eutherians. We thus argue there are two modes of miR-615 production. RNA-seq data indicate that mature products of *mir*-*615* are detected in many human cell lines and tissues with no *HOXC5* expression. This independent transcription of *mir*-*615* most likely occurs through the use of separate transcriptional control sequences which do not result in production of a functional host transcript (Fig. 4). It has been reported that most miRNA promoters are located within 500 bp upstream of the pre-miRNA sequence [35], suggesting the putative miR-615 specific promoter lies close to the start of the intron or in the first coding exon of *HOXC5*. The region upstream of *mir*-*615*, within the limits of the large CpG island spanning most of the *HOXC5* locus (Fig. 4), undergoes DNA methylation in pancreatic cancer cell lines and is reported as including a promoter for *mir*-*615* [36] although no precise promoter elements were identified. Histone methylation, DNase I hypersensitivity and CAGE tag data, combined with analysis of ESTs, points to a probable transcription initiation site within the first exon, approximately 550 bp upstream of *mir*-*615* (Fig. 4). As the majority of the *HOXC5* locus including the candidate miRNA promoter lies within the CpG island, it is likely that transcription is driven over a broad ranges of TSSs rather than a precise initiation site [37, 38].

We hypothesise that the acquisition of the CpG island in eutherian *Hoxc5* could have facilitated the evolution of independent promoter sequences for *mir*-*615*. The existence of TSSs located within exons has been noted [39], and is hypothesised as a significant contributor to truncated non-coding mRNAs [40]. Internal initiation leading to the production of such transcripts may have enabled *mir*-*615* to be expressed independently of its host gene. While this would have occurred initially by chance, it would afford subsequent selection the opportunity to increase expression levels, perhaps in a tissue-specific manner. CpG island-associated promoters are known to evolve rapidly [37], which would promote the evolution of *mir*-*615* regulation. Interestingly, the CpG island covering the putative TSS for *mir*-*615* along with much of the *Hoxc5* locus in eutherians seems to be absent in marsupials. In the Tasmanian devil (*Sarcophilus harrisii*), the corresponding region is not annotated as a CpG island, and the GC content of the *Hoxc5* intron is lower in marsupials than in eutherians (52 % in Tasmanian devil and tammar wallaby compared to 60 % in human, 60 % in mouse and 62 % in cow). It is more likely that the CpG island was gained in eutherians, rather than present in a common ancestor and lost in marsupials [41].

### Putative functions for miR-615

Several studies have explored the roles of miR-615 in the context of human pathologies [36, 42–45]. The miRNA is known to be highly expressed in the prostate cancer cell line LNCaP relative to normal prostate epithelial cells [44], as well as in cirrhotic and cancerous liver tissues and cell lines but not healthy liver [43]. miR-615-3p is also one of five Hox cluster miRNAs significantly upregulated in the prefrontal cortex in patients with Huntington’s Disease [45]. It is likely that in these contexts, miR-615-5p is acting as a tumour suppressor [36, 43] through interfering with the *insulin*-*like growth factor 2* (*IGF2*) transcript. However, the involvement of miR-615 in non-pathologic biological processes is currently unknown.

The conservation of *mir*-*615* across the Eutheria is indicative of biological function. Our analysis of data from Chiang et al. indicates that whole-body expression of miR-615 increases throughout mouse embryonic development and persists in the newborn [21]. Although expression levels across replicates in this study were somewhat variable, the greatest increase in expression occurs between E9.5 and E12.5. miR-615 is also amongst the 10 miRNAs which are most upregulated upon differentiation of human embryonic stem cells [46]. Known tissues in which miR-615 expression is conserved in more than one eutherian species include the cerebellum, kidney and gonads. While the observation that miR-615 is strongly upregulated in goat ovaries during pregnancy [27] is striking, these data come from a single biological replicate and should be interpreted with caution.

We also find that GO terms associated with regulation of growth and development are overrepresented by in a high stringency target prediction dataset for miR-615-3p. This observation, coupled with the high expression of miR-615-3p in the mouse embryo, suggests a potential role for miR-615-3p in the regulation of growth and development during embryogenesis. Although the expression of *Hoxc5* during axial patterning is likely to result in production of miR-615-3p and may account for some of the observed expression, axial Hox gene expression is initiated prior to E9.5 [47] and is unlikely to explain the upregulation of the miRNA in between E9.5 and E12.5. We hypothesize that the observed increase in expression results from either upregulation through the host gene promoter elsewhere in the developing embryo, or through the miRNA-specific promoter. The observation that ovaries from newborn mice express miR-615-3p at a relatively high level [48] suggests that some of this may result from ovarian expression of miR-615. Other major developmental events occurring between E9.5 and E12.5 include organogenesis of the kidney [49, 50], sex-specific differentiation of the gonads [51] and outgrowth of the limb primordia [52]. A study on the embryonic gonads of the sheep (*Ovis aries*) lends some weight to the hypothesis that miR-615 may be involved in gonadal differentiation [28], since the miRNA is identified as expressed at significantly higher levels (fold change ≥2) in ovaries at early gestation (42 days) than in mid-gestation. This difference is not observed in the foetal testis. However, caution should be applied in interpretation of data across different species. Another possibility is that miR-615 is involved in the modulation of embryonic growth; studies on human cancer cell lines have implicated this miRNA in the regulation of cell growth, proliferation and migration [36, 43].

## Conclusions

The Hox cluster miRNA *mir*-*615* represents an interesting case of acquisition of a novel miRNA by intronic exaptation and an unusual example of a new functional gene arising within a conserved Hox gene cluster. We demonstrate using sequence analysis and structural prediction that miR-615 is a eutherian-specific miRNA, although a similar sequence exists in extant marsupials that may have a low probability of miRNA processing. Using a data mining approach combining epigenetic studies with RNAseq and CAGE tag data, we show that miR-615 can be expressed under the control of a Hox gene promoter, as well as independently from its host gene transcript. Based on these observations, we propose a model for intronic miRNA evolution through selection on intronic secondary structure followed by evolution of an independent miRNA promoter. Target prediction data support the involvement of miR-615 in growth and developmental pathways.

## Additional files

10.1186/s13227-015-0027-1 Position of forward and reverse primers used to amplify the *Hoxc5* intronic region in Xenarthra. Primers were designed against highly conserved regions within the *Hoxc5* coding sequence. Corresponding sequences for each vertebrate species are shown.
10.1186/s13227-015-0027-1 Datasets used in analysis of *mir*-*615* and *Hoxc5* expression.
10.1186/s13227-015-0027-1 (A) Alignment of tetrapod *Hoxc5* loci including exonic regions. (B) RNAz analysis for eutherian pre-miR-615.
10.1186/s13227-015-0027-1 Predicted secondary structures and minimum free energies for the region corresponding to *mir*-*615* across therian mammals.
10.1186/s13227-015-0027-1 Cell lines available through the ENCODE Consortium (2003–2012) for which small RNA sequencing data are available.
10.1186/s13227-015-0027-1 (A) Read counts for miR-615 mature products in the GM12878 and K562 cell lines, as generated by the miRDeep2 quantifier.pl script. Raw small RNA sequencing data were obtained from the ENCODE Consortium. (B) Data from Zhang et al. (2013) showing normalized tissue-specific read counts for the dominant product of miR-615 (miR-615-3p). Sertoli cells, spermatocytes and spermatids were only sampled in mouse [27]. (C) Read counts for both mature products of miR-615 in pooled ovarian sample from six pregnant and six non-pregnant goats. The dataset used for this analysis was obtained from Zhang et al. (2013).
10.1186/s13227-015-0027-1 miR-615-3p read counts in mouse small RNA libraries as reported in the dataset of Chiang et al. (2010), refer to [21]. Data were obtained through miRBase.
10.1186/s13227-015-0027-1 RPKM values for *HOXC* genes in a K562 transcriptome assembly. Raw reads were obtained through the ENCODE Consortium.
10.1186/s13227-015-0027-1 PITA prediction targets for miR-615-3p.
10.1186/s13227-015-0027-1 GO terms identified in the miR-615-3p target prediction dataset. The normalised frequency of each term is plotted relative to its frequency in a set of all human cDNAs. The dotted line indicates equal frequency of expression in both datasets.

## Abbreviations

BLAST: basic local alignment search tool
BLASTn: nucleotide BLAST
Bp: base pair
cDNA: complementary DNA
CITES: convention on international trade in endangered species of wild fauna and flora
ENCODE: encyclopedia of DNA elements
EST: expressed sequence tag
FPKM: fragments per kilobase of transcript per million mapped reads
GEO: gene expression omnibus
miRNA: microRNA
nt: Nucleotide
PCR: polymerase chain reaction
PITA: probability of interaction by target accessibility
ssRNA: single-stranded RNA
TSS: transcription start site
UTR: untranslated region
