# Supplementary material for: The Hox cluster microRNA miR-615: a case study of intronic microRNA evolution
Source: EvoDevo. 2015 Oct 7;6:31. doi: 10.1186/s13227-015-0027-1 (PMC4597612; doi:10.1186/s13227-015-0027-1)

**Supplement S1**

Position of forward and reverse primers used to amplify the *HoxC5* intronic region in Xenarthra. Primers were designed against highly conserved regions within the *HoxC5* coding sequence. Corresponding sequences for each vertebrate species are shown.


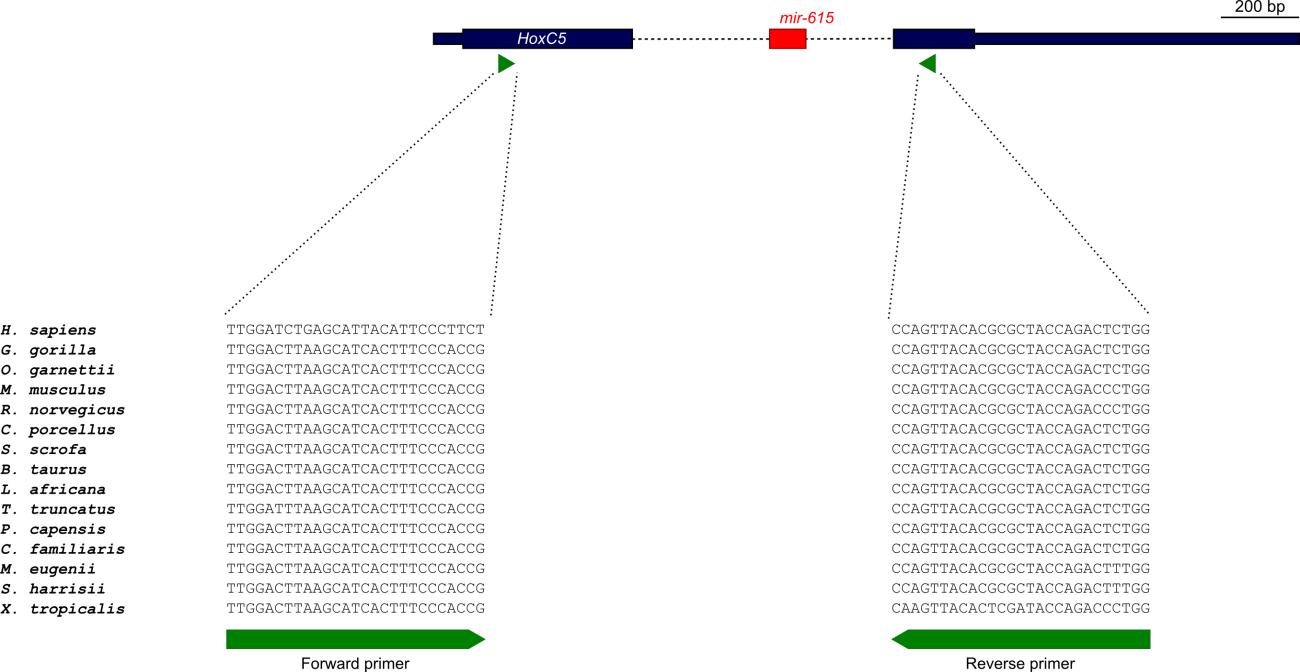

Supplement: Supplementary file 1 — 10.1186/s13227-015-0027-1 Position of forward and reverse primers used to amplify the Hoxc5 intronic region in Xenarthra. Primers were designed against highly conserved regions within the Hoxc5 coding sequence. Corresponding sequences for each vertebrate species are shown. [file 13227_2015_27_MOESM1_ESM.docx]
