# Supplementary material for: The Hox cluster microRNA miR-615: a case study of intronic microRNA evolution
Source: EvoDevo. 2015 Oct 7;6:31. doi: 10.1186/s13227-015-0027-1 (PMC4597612; doi:10.1186/s13227-015-0027-1)
Supplement: Supplementary file 2 — 10.1186/s13227-015-0027-1 Datasets used in analysis of mir-615 and Hoxc5 expression. [file 13227_2015_27_MOESM2_ESM.docx]

**Supplement S2**

Datasets used in analysis of *mir-615* and *Hoxc5* expression

| **Accession number** | **Tissue/Cell line** | **Species** | **Type** |
| --- | --- | --- | --- |
| ENCFF917ANZ | Cerebellum (foetal, 37 weeks and 19 weeks) | *Homo sapiens* | RNA-seq |
| ENCSR046XHI | Ovary | *Homo sapiens* | RNA-seq |
| ENCSR516UNF | Ovary (age = 10 weeks) | *Mus musculus* | RNA-seq |
| GSM874239 | Ovary (adult) | *Mus musculus* | Microarray |
| GSM874241 | Ovary (neonatal) | *Mus musculus* | Microarray |
| GSM35553 | Ovary (commercial RNA) | *Mus musculus* | Microarray |
| GSE22776 | Ovarian follicles | *Macaca mulatta* | Microarray |
| ENCSR109IQO | K562 | *Homo sapiens* | RNA-seq |
| ENCSR000AET | K562 | *Homo sapiens* | Small RNA-seq |
| ENCSR000AJG | GM12878 | *Homo sapiens* | Small RNA-seq |
