# Supplementary material for: The Hox cluster microRNA miR-615: a case study of intronic microRNA evolution
Source: EvoDevo. 2015 Oct 7;6:31. doi: 10.1186/s13227-015-0027-1 (PMC4597612; doi:10.1186/s13227-015-0027-1)

**Supplement S3A**

CLUSTAL X (1.81) multiple sequence alignment

Homo_sapiens --------------------------------------------------

Gorilla_gorilla --------------------------------------------------

Otolemur_garnettii --------------------------------------------------

Mus_musculus CTCCAGTCACATCCCAGAGAGTACCTAGGACTCTCAGCCTCAGGACCTGT

Rattus_norvegicus --------------------------------------------------

Jaculus_jaculus --------------------------------------------------

Cavia_porcellus --------------------------------------------------

Ochotona_princeps --------------------------------------------------

Sorex_araneus --------------------------------------------------

Sus_scrofa --------------------------------------------------

Bos_taurus --------------------------------------------------

Tursiops_truncatus --------------------------------------------------

Canis_familiaris --------------------------------------------------

Loxodonta_africana --------------------------------------------------

Procavia_capensis --------------------------------------------------

Choloepus_didactylus --------------------------------------------------

Myrmecophaga_tridactyla --------------------------------------------------

Macropus_eugenii --------------------------------------------------

Sarcophilus_harrisii --------------------------------------------------

Anolis_carolinensis --------------------------------------------------

Xenopus_tropicalis --------------------------------------------------

Homo_sapiens --------------------------------------------------

Gorilla_gorilla --------------------------------------------------

Otolemur_garnettii --------------------------------------------------

Mus_musculus CTGCTGCTTCTGCTCATTCAGGAACCCTCTCTCCTCTTGGGTATCCTGCC

Rattus_norvegicus --------------------------------------------------

Jaculus_jaculus --------------------------------------------------

Cavia_porcellus --------------------------------------------------

Ochotona_princeps --------------------------------------------------

Sorex_araneus --------------------------------------------------

Sus_scrofa --------------------------------------------------

Bos_taurus --------------------------------------------------

Tursiops_truncatus --------------------------------------------------

Canis_familiaris --------------------------------------------------

Loxodonta_africana --------------------------------------------------

Procavia_capensis --------------------------------------------------

Choloepus_didactylus --------------------------------------------------

Myrmecophaga_tridactyla --------------------------------------------------

Macropus_eugenii --------------------------------------------------

Sarcophilus_harrisii --------------------------------------------------

Anolis_carolinensis --------------------------------------------------

Xenopus_tropicalis --------------------------------------------------

Homo_sapiens --------------------------------------------------

Gorilla_gorilla --------------------------------------------------

Otolemur_garnettii --------------------------------------------------

Mus_musculus TACCTCCCAGAGGTTAATTTTTCCAAGCATACAAGACAAGCTTCTCTTTA

Rattus_norvegicus --------------------------------------------------

Jaculus_jaculus --------------------------------------------------

Cavia_porcellus --------------------------------------------------

Ochotona_princeps --------------------------------------------------

Sorex_araneus --------------------------------------------------

Sus_scrofa --------------------------------------------------

Bos_taurus --------------------------------------------------

Tursiops_truncatus --------------------------------------------------

Canis_familiaris --------------------------------------------------

Loxodonta_africana --------------------------------------------------

Procavia_capensis --------------------------------------------------

Choloepus_didactylus --------------------------------------------------

Myrmecophaga_tridactyla --------------------------------------------------

Macropus_eugenii --------------------------------------------------

Sarcophilus_harrisii --------------------------------------------------

Anolis_carolinensis --------------------------------------------------

Xenopus_tropicalis --------------------------------------------------

Homo_sapiens --------------------------------------------------

Gorilla_gorilla --------------------------------------------------

Otolemur_garnettii --------------------------------------------------

Mus_musculus TCAAAACTGAGTTCAATCCTCCCAAGCAAGAGACCTTGACCTTTAGTTTA

Rattus_norvegicus --------------------------------------ACCTTTAGTTTA

Jaculus_jaculus --------------------------------------------------

Cavia_porcellus --------------------------------------------------

Ochotona_princeps --------------------------------------------------

Sorex_araneus --------------------------------------------------

Sus_scrofa --------------------------------------------------

Bos_taurus -------------------------------------------------A

Tursiops_truncatus --------------------------------------------------

Canis_familiaris --------------------------------------------------

Loxodonta_africana --------------------------------------------------

Procavia_capensis --------------------------------------------------

Choloepus_didactylus --------------------------------------------------

Myrmecophaga_tridactyla --------------------------------------------------

Macropus_eugenii --------------------------------------------------

Sarcophilus_harrisii --------------------------------------------------

Anolis_carolinensis --------------------------------------------------

Xenopus_tropicalis --------------------------------------------------

Homo_sapiens --------------------------------------------------

Gorilla_gorilla --------------------------------------------------

Otolemur_garnettii --------------------------------------------------

Mus_musculus GGTAACCCAAGATATGTCCTCCTGGCTCTCCACGCTGGGCTTTAGCTACC

Rattus_norvegicus GGTAACCCAAGATA-ATCCTCTTGGTTCTCCACCCTGGGCTTTAGCTACT

Jaculus_jaculus --------------------------------------------------

Cavia_porcellus --------------------------------------------------

Ochotona_princeps --------------------------------------------------

Sorex_araneus --------------------------------------------------

Sus_scrofa --------------------------------------------------

Bos_taurus TTTAGAGAACCTGAGATAAGGCTCCTCCAGCCTGGGTTGGGGCTCTCCTA

Tursiops_truncatus --------------------------------------------------

Canis_familiaris --------------------------------------------------

Loxodonta_africana --------------------------------------------------

Procavia_capensis --------------------------------------------------

Choloepus_didactylus --------------------------------------------------

Myrmecophaga_tridactyla --------------------------------------------------

Macropus_eugenii --------------------------------------------------

Sarcophilus_harrisii --------------------------------------------------

Anolis_carolinensis --------------------------------------------------

Xenopus_tropicalis ------------------------------ATATAGCAATTTATGGATCA

Homo_sapiens --------------------------------------------------

Gorilla_gorilla -------------------CATTTTTGAAGATTGGAAGACCCTGGAGGTT

Otolemur_garnettii ------------CCTCAGCCATTTTTGAAGATTAGAAGACCACTGAGATT

Mus_musculus -CCAACTCTCTTTCTCAGTCATCATTGAAGTTTGGAAGGCTCTGGAGGTA

Rattus_norvegicus ACCCCCAAGTCTCCTCAGTCATCATTGAAGTTTGCAAGGCGCTGGAGATT

Jaculus_jaculus --------------------------------------------------

Cavia_porcellus --------------------------GAAGTTTAGCAGACTTAGGAATTA

Ochotona_princeps --------------------------------------------------

Sorex_araneus --------------------------------------------------

Sus_scrofa --------------------------------------------------

Bos_taurus CCTCAATCCCCTTCTCAGCCATTTTTGAACATTGAAACTCCCTGGGGTTG

Tursiops_truncatus ----------------------------------GAAAACCCCGAAAAAA

Canis_familiaris --------------------------------------------------

Loxodonta_africana -------------------CATTTTTGAGTACGGGAAGACCCTGGAAGTG

Procavia_capensis ------------CTTCAGTCTTTTTTGAGTACTAGAAGGCCCTGGAAGTG

Choloepus_didactylus --------------------------------------------------

Myrmecophaga_tridactyla --------------------------------------------------

Macropus_eugenii --------------------------------------------------

Sarcophilus_harrisii ---------------------TCCAGCGAACCTAGAAGGCCCTGGGGA--

Anolis_carolinensis --------------------------------------------------

Xenopus_tropicalis CAAAGGCAATTATTACTACGCGCAATTAATTTCCTGAGAGCATCAAGTTA

Homo_sapiens --------------------------------------------------

Gorilla_gorilla GG-----GAGAACGGGGCTTTCATTAC---A-AAAAGCACTCTGTGCATT

Otolemur_garnettii GAG----GGAATGGGATTATCCATTACAAAATAAAAACACGATGTGCATC

Mus_musculus GG-----GAACTGGCACTATTCGTTTC---AAAAACCACCGGTGTAATTC

Rattus_norvegicus GG-----GGACTGGCACTATCCATTAC---GAAAACCACCAGAGTAATTC

Jaculus_jaculus --------------------------------------------------

Cavia_porcellus GG-----TAGAACGGGCTATACATTAC--AA-AAATCACCGCTGTACATC

Ochotona_princeps --------------------------------------------------

Sorex_araneus --------------------------------------------------

Sus_scrofa ----------ACAGGGCTATCTGTTAC-CAAAAAAACCTCTCTGTACATC

Bos_taurus TGG----AGGATGGGGCTATCCGTTAT---CAAAAAACGCTCTGCGCATC

Tursiops_truncatus CA-----GAAAACAAAAAACCAAAAAA-CAGAACAAACCCTCTGTGCATC

Canis_familiaris -----------CGGGGCTATCCGCTAG-------------TGCTTGCACC

Loxodonta_africana GAGGGGTGGGGGGGAACTATCCATTAC----AAAAGTCGCTTTGCGCACC

Procavia_capensis G------GGGGAAGGGCTATACATTAC----AAAAGCCTCTTTCCACATG

Choloepus_didactylus --------------------------------------------------

Myrmecophaga_tridactyla --------------------------------------------------

Macropus_eugenii --------------------------------------------------

Sarcophilus_harrisii -------AGGGAGGGGCGACGATCTATTCAGAAAAGCCCCTCTGCGCATC

Anolis_carolinensis --------------------------------------------------

Xenopus_tropicalis AATAAAAGCGTCCTTACTTTTACTGGGGAAAATAAAAAATGAAACGGCTC

Homo_sapiens --------------------------------------------------

Gorilla_gorilla ATAAGTGGGGCCTCATCTTTTCCCAATTTTAAATAGAATTTAAAAGGCTA

Otolemur_garnettii CTAAGTGGAGCCTCATCTTTTCTCAAATTTAAATAGAATTTAAAAGGCTA

Mus_musculus ATAAATAAGGCCTCATCCTTTCTCAATTTTAAGCAGAATTTTAAAGACAA

Rattus_norvegicus TTAAATAAGGTCTCATCTTTTCTGAAATTTTAACAGAATTTTAAAGACAA

Jaculus_jaculus --------------------------------------------------

Cavia_porcellus GTAAGTGAGACCTCATCTCTTCTCCAATTTAAGTAGAATTGCAAGGGCCT

Ochotona_princeps --------------------------------------------------

Sorex_araneus --------------------------------------------------

Sus_scrofa CTAG--TGAAGCTCATCTCCCCTTAAAATTAAATATAATTTAAAAGGCCA

Bos_taurus CT-GGTGAAGGCTTATCTCCAGTTAAAATTAAATACAATTTAAAAGGCCA

Tursiops_truncatus CT-AGTGAAGCCTCATCTCCCCTTAAAATGAAATACAATTTAAAAGGCCA

Canis_familiaris CTAAGCAAGACCTCATCTTTCCTTAAAATTAAAGACAATTTAAAAGGCCA

Loxodonta_africana GTAAATGAAACCCCATCTTTTCTTAAAACTAAATACAATCTAAAAGGCCA

Procavia_capensis CTAAATGAAGCCCCATCTTTTCTTAAAATTAAATACAATCTAAAAGGCCA

Choloepus_didactylus --------------------------------------------------

Myrmecophaga_tridactyla --------------------------------------------------

Macropus_eugenii --------------------------------------------------

Sarcophilus_harrisii CTAAATGAGGCCCCATCTTTCCTTAAAATTAAATACAATTTAAAAGGCCA

Anolis_carolinensis --------------------------------------------------

Xenopus_tropicalis CTCCAACCAGTGAGATCCTTATCCATAATTAGGCGACCATTACAATTAAA

Homo_sapiens --------------------------------------------------

Gorilla_gorilla GCTCAGGG--------------GTTGCCC-CAACAACCA-----------

Otolemur_garnettii GCTCAGGGGTTT----------GTTGCTGTTAACAACCA-----------

Mus_musculus GCTCAATGGGTT----------AGTTTGTCCAACAAGCA-----------

Rattus_norvegicus GCTCAA-GGGTT----------AGTTTGTCCAACAAGCA-----------

Jaculus_jaculus --------------------------------------------------

Cavia_porcellus GCCCAGGAAATC----------TTTGCAG-CAGCAACTA-----------

Ochotona_princeps --------------------------------------------------

Sorex_araneus --------------------------------------------------

Sus_scrofa TTTCAGGGGTTT----------GTTGCAT-CAACACCCA-----------

Bos_taurus TTTCAGGAGTTT----------ATTGCAG-CAACACCCA-----------

Tursiops_truncatus TTTCAGTGGTTT----------GTTGCAG-CAACACCCA-----------

Canis_familiaris TTTCAAGGGTTT----------GTTGCAG-CAGCAGCAGCAGCAGCAGCA

Loxodonta_africana GCTGGGAGGGGTGGGAGGG---TTTACAG-CAACAGCTA-----------

Procavia_capensis GCTTGGGAGAAGGGGAAAGGTTGTTGCCA-CAACAGCCA-----------

Choloepus_didactylus --------------------------------------------------

Myrmecophaga_tridactyla --------------------------------------------------

Macropus_eugenii --------------------------------------------------

Sarcophilus_harrisii ACTCAGGGGGGT----------TTGTGTAAAACAGCACA-----------

Anolis_carolinensis --------------------------------------------------

Xenopus_tropicalis GAGTGGTAGTTGATAGAATATAGATGTAAAATTTTAGTA-----------

Homo_sapiens --------------------------------------------------

Gorilla_gorilla -----GCTTCTCCTTCACCGTGTAGGAAATCC--AGCCACCGGAAAGCAA

Otolemur_garnettii -----GCTTCTCC---ACAGGGAAGAAAATTC--AGCCTCCTGAAGGCGT

Mus_musculus -----ACAACTTCTCCACAAACT-----------GACTGCTAGAAGGCCA

Rattus_norvegicus -----ACAACTTCTCTACAATCT-----------GACTGCCAGAAGACCA

Jaculus_jaculus --------------------------------------------------

Cavia_porcellus -----CTTTCTCC---ACGCGATAGAAAATCC--AGCCACCAAAAGGCGA

Ochotona_princeps -----------------------------------------------GCA

Sorex_araneus --------------------------------------------------

Sus_scrofa -----GCCTCTTC---ACAGGAGAAAAAGCCA----AGGCCTGAAGGCCA

Bos_taurus -----GCTTCTCCTCTACAGGGTAGAAAATCCTGAGAGGCCTGAAGGCCA

Tursiops_truncatus -----GCTTCTCCTCTACAGCGTGGAAAATCC--AGAGTCCTGAAGGCCA

Canis_familiaris GCAACCAGCCTTCTCTACAGGGTAGAAAATCC--AGCGGTCTGAAGGCGG

Loxodonta_africana -----GTGTCTCC---ACATGGTAGAAAATTC--------------AGCC

Procavia_capensis -----GTGTCTCC---ACAGGGCAGAAAATTA--AACCTCCTGCG-----

Choloepus_didactylus --------------------------------------------------

Myrmecophaga_tridactyla --------------------------------------------------

Macropus_eugenii --------------------------------------------------

Sarcophilus_harrisii -----GCTTCTCTTCGCTAGGGTAGAAAAATCC-ACCCAGGAGAGGCGAG

Anolis_carolinensis --------------------------------------------------

Xenopus_tropicalis ---------AATAGTCAAAATCATTGACCTACAAGAGCCATAGTGGCGCA

Homo_sapiens --------------------------------------------------

Gorilla_gorilla GCTGGCGCTCCAGGATGCAATTCCCCCACATAGGCACCAGGTGTCGCTGT

Otolemur_garnettii GTTAGAGCTCGAGGATGCAATTTCTCCAAGTAGGCACCAGGTGTCGCTGT

Mus_musculus AGCGATGCTACAAGATGAAATTCCTCCGAGTCGGCACCAGGTGTCGCTGT

Rattus_norvegicus AGCGATGCTAC-AGATGCAATTCCTCCAAGTCGGCACCAGGTGTCGCTGT

Jaculus_jaculus --------------------------------------------------

Cavia_porcellus GCAGCAGCATCATGATGCAATTCCTACAAGTAGACACCAGGTGTCGCTGT

Ochotona_princeps GCCGGCGCGGCAGGATGCAATTCCTACAAGTAGGCACCAGGTGTCGCTGT

Sorex_araneus --------------------------------------------------

Sus_scrofa GCCAGAGAGCCAGGCTGCAATTCCTCGAAGTAGGCACCAGGTGTCGCTGT

Bos_taurus GCCAGCGAGCCAGGATGCAATTCCTCCAAGTAGGCACCAGGTGTCGCTGT

Tursiops_truncatus GCCAGCGAGCCAGGCTGCAATTCCTCCAAGGAGGCACCAGGTGTCGCTGT

Canis_familiaris GCACACAAGCCAGGCTGCAATCCCCCCAAGTAGGCACCGGGTGTCGCTGT

Loxodonta_africana GCCAGCTCTCCAGGCTGCAATTCCTCCATGTCGGCACCAGGTGTCGCTGT

Procavia_capensis --------TCTAAACTGCAATTCCTCCATGTCGGCACCAGGTGTCGCTGT

Choloepus_didactylus --------------------------------------------------

Myrmecophaga_tridactyla --------------------------------------------------

Macropus_eugenii --------------------------------------------------

Sarcophilus_harrisii GATGACAAGCCAGGATGCAATACCGCCGAGCCTACGCCAGGTGTCGCTGT

Anolis_carolinensis --------------------------------------------------

Xenopus_tropicalis GCCATGTTCAGCAGGTGCTCTACCACCCAACTGGCACCAGGTGACGCTGT

Homo_sapiens --------------------------------------------------

Gorilla_gorilla GGGCTTGTTGTCCCGGCTA-CCCCCAATTCCAAGAACCTTTTTTTTCCCC

Otolemur_garnettii GAGCTCGTTGTCCCGGCTACCCCCCAATTCCAAGAACCATTTTTTTCTCC

Mus_musculus GAGCTCGTTGTCCCGACTACCCCCCAATTCCAAGAACCATTTTTTTTTCC

Rattus_norvegicus GAGCTCGTTGTCCCGACTACCCCCCAATTCCAAGAACCA---TTTTTTCC

Jaculus_jaculus --------------------------------------------------

Cavia_porcellus GGGCTCGTTGTCCCGCCTACCCCCCAATTCCAAGAACCATTTTTTTTTTT

Ochotona_princeps GGACTCGTTGTGTCGGCTACCCCCC-ATCCCAAGAACCATTTTTTTTCCT

Sorex_araneus --------------------------------------------------

Sus_scrofa GGGCTCGTTGTCCCGGCTACCCCCCAATTCCAAGAACCATTTTTTTTTTC

Bos_taurus GGGCTTGTTGTCCCGGCTACCCCCCAATTCCAAGAACCATTTTTTTTTTC

Tursiops_truncatus GGGCTCGTTGTCCCGCCTACCCCCCAATTCCAAGAAGCTTTTTTTTTTTT

Canis_familiaris GGGCTCGTTGTCCCGGCTACCCCCCAATTCCAAGAACCATTTTTTTCTCC

Loxodonta_africana GGGCTCGTTGTCCCGGCTACCCCCCAATTCCAAGAACCATTTTTTTTTTC

Procavia_capensis GAGCTCGTTGTCCCAGCTACCCCCCAATTCGAAGAGCGATTTTTTTTTCT

Choloepus_didactylus --------------------------------------------------

Myrmecophaga_tridactyla --------------------------------------------------

Macropus_eugenii --------------------------------------------------

Sarcophilus_harrisii GGACTCGTTGTCCCCGCTACCCCCTAATTCCAACAACCATTTTTTTTTCT

Anolis_carolinensis --------------------------------------------------

Xenopus_tropicalis GGTCTCATTGTCTTCTTT--------------------------------

Homo_sapiens --------------------------------------------------

Gorilla_gorilla TCCCCCTTC-----------------------------------CCTCTT

Otolemur_garnettii CCCTTCACT-----------------------------------------

Mus_musculus TCCCCCTTG-----------------------------------CTTCTC

Rattus_norvegicus TCCCCCTTG-----------------------------------CTTCTC

Jaculus_jaculus --------------------------------------------------

Cavia_porcellus CTCCCCCTT------------------------------CCCTCTTTTTC

Ochotona_princeps CCCCCCTCC-----------------------------TCCTCTCCCTTT

Sorex_araneus --------------------------------------------------

Sus_scrofa CTCCCCCTTCTCTCCCTTTCTCTCTCTCTCTCTCTCTCTCTCTCTCTCTC

Bos_taurus TCCCCCTTC-------------------------------TCTCCTTTTC

Tursiops_truncatus CTCCCCCTT----------------------------CTCTCTCTTTCTC

Canis_familiaris CCCTTCTCT--------------CTCTCTCTCTCTCTCCCTCTCTCTCTC

Loxodonta_africana TCCCCCTTC--------------------------------------ACT

Procavia_capensis CCCCCTTCC----------------------------------------C

Choloepus_didactylus --------------------------------------------------

Myrmecophaga_tridactyla --------------------------------------------------

Macropus_eugenii --------------------------------------------------

Sarcophilus_harrisii CCCCCTTCT----------------------------------------C

Anolis_carolinensis --------------------------------------------------

Xenopus_tropicalis --------------------------------------------------

Homo_sapiens --------------------------------------------------

Gorilla_gorilla TCTCTCTCTCA-CTCCCTCT-CCCACTTGGTTGGGCTTTGCCAACATATC

Otolemur_garnettii -------CTCT-TTCTCTCT-CCCCCTTAGTTGGGCTTTGCCAACATATC

Mus_musculus TTTCTCTTTCA-TCCCCCCCCCCCCAACCCCCGGGCTTTGCCAACATATC

Rattus_norvegicus TTTCTCTTTCAGCTCCCCCCCCCCCCGCCCCACGGCTTTGCCAACATATC

Jaculus_jaculus --------------------------------------------------

Cavia_porcellus TCTGTCTCTGA-CTCCCTCT-CCCCCTTGGTTAGGCTTTGCCAACATATC

Ochotona_princeps CTCTACTCTCA-CTCCCTCT-CCCCCTTGGTTGGGCTTTGCCAACATATC

Sorex_araneus --------------------------------------------------

Sus_scrofa TCTCACTCTCA-CTCCCTCA-CCCCCTTGGTTGGGCTTTGCCAACATATC

Bos_taurus TCTCTCTCTCA-CTCCCTCT-CCCCCTTGGTTGGGCTTTGCCAACATATC

Tursiops_truncatus TCTCTCCCTCA-CTCCCTCT-CCCCCTTGGTTGGGCTTTGCCAACATATC

Canis_familiaris TTTCTCTCTCA-CTCCCTCTCCCCCCTTGGTTGGGCTTTGCCAACATATC

Loxodonta_africana CTTTTCTCTGA-CTCCCTCT-CCCCCTTGGTTGGGCTTTGCCAACATATC

Procavia_capensis TGTTTCTCGCA-CTCCCTCT-CCCCCTTGGTTGGGCTTTGCCAACATATC

Choloepus_didactylus --------------------------------------------------

Myrmecophaga_tridactyla --------------------------------------------------

Macropus_eugenii --------------------------------------------------

Sarcophilus_harrisii TCCCTTTCTCT-CGATCCCTCTCCCCTCGGTTGGGCTGTGCCAACATATC

Anolis_carolinensis --------------------------------------------------

Xenopus_tropicalis ------------TCCAACTTGGGGCCTTCGGTTTGTTCCGCCAACATATC

Homo_sapiens --------------------------------------------------

Gorilla_gorilla GAGATGCGTTTCGCCGGCTTCCATCACTAACCTCCCGGAGGTCATCAAGC

Otolemur_garnettii AAGATGCGTTTTGCCGGCTTCCATCACTAACCTCCCGGAGGTCATCAAGC

Mus_musculus AAGATGCGTTTCGCCGGCTTCCATCACTAACCTCCCGGAGGTCATCAAGC

Rattus_norvegicus AAAATGCGTTTCGCCGGCTTCCATCACTAACCTCCCGGAGGTCATCAAGC

Jaculus_jaculus --------------------------------------------------

Cavia_porcellus AAGATGCGTTTCTCCGGCTTCCATCACTAACCTCCCGGAGGTCATCAAGC

Ochotona_princeps AAGATGGGTTTCGCCGGCTTCCATCACTAACCTCCCGGAGGTCATCAAGC

Sorex_araneus --------------------------------------------------

Sus_scrofa AAGATGCGTTTCGCCGGCTTCCATCACTAACCTCCCGGAGGTCATCAAGC

Bos_taurus AAGATGCGTTTCGCCGGCTTCCATCACTAACCTCCCGGAGGTCATCAAGC

Tursiops_truncatus AAGATGCGTTTCGCCGGCTTCCATCACTAACCTCCCGGAGGTCATCAAGC

Canis_familiaris AAGATGCGTTTCGCCGGCTTCCATCACTAACCTCCCGGAGGTCATCAAGC

Loxodonta_africana AAGATGCGTTTCGCCGGCTTCCATCACTAACCTCCCGGAGGTCATCAAGC

Procavia_capensis AAGATGCGTTTCGCCGGCTTCCATCACTAACCTCCCGGAGGTCATCAAGC

Choloepus_didactylus --------------------------------------------------

Myrmecophaga_tridactyla --------------------------------------------------

Macropus_eugenii --------------------------------------------------

Sarcophilus_harrisii AAGATGCGTTTCGCCGGCTTCCATCACTAACCTCCCGGAGGTCATCAAGC

Anolis_carolinensis --------------------------------------------------

Xenopus_tropicalis -AGATACGTTTCACAGGCTTCCATCAATAACCTCCTGG-GGTCATCAAGC

Homo_sapiens --------------------------------------------------

Gorilla_gorilla CAAATTTATGAGTGGCCGCTCGAGTCACGTGACTCTATTTAAGGCTCCCT

Otolemur_garnettii CAAATTTATGAGTGGCCGCTGGAGTCACGTGACTCTATTTAAGGCTCCCT

Mus_musculus CAAATTTATGAGTGGCCGCTCCAGTCACGTGACTCTATTTAAGGCTCCCT

Rattus_norvegicus CAAATTTATGAGTGGCCGCTCCAGTCACGTGACTCTATTTAAGGCTCCCT

Jaculus_jaculus --------------------------------------------------

Cavia_porcellus CAAATTTATGAGTGGCCGCTCGAGTCACGTGACTCTATTTAAGGCTCCCT

Ochotona_princeps CAAATTTATGAGTGGCCGCCGGAGTCACGTGACTCTATTTAAGGCTCCCT

Sorex_araneus --------------------------------------------------

Sus_scrofa CAAATTTATGAGTGGCCGCTCGAGTCACGTGACTCTATTTAAGGCTCCCT

Bos_taurus CAAATTTATGAGTGGCCGCTCGAGTCACGTGACTCTATTTAAGGCTCCCT

Tursiops_truncatus CAAATTTATGAGTGGCCGCTCGAGTCACGTGACTCTATTTAAGGCTCCCT

Canis_familiaris CAAATTTATGAGTGGCCGCTCGAGTCACGTGACTCTATTTAAGGCTCCCT

Loxodonta_africana CAAATTTATGAGTGGCCGCTCCAGTCACGTGACTCTATTTAAGGCTCCCT

Procavia_capensis CAAATTTATGAGTGGCCGCTCCAGTCACGTGACTCTATTTAAGTCTCCCT

Choloepus_didactylus --------------------------------------------------

Myrmecophaga_tridactyla --------------------------------------------------

Macropus_eugenii --------------------------------------------------

Sarcophilus_harrisii CAAATTTATGAGTGGCCGCCCGAGTCACGTGACTCTATTTAAGGATCCCT

Anolis_carolinensis --------------------------------------------------

Xenopus_tropicalis CAAATTTATGACTGGCCAACAGAATCACGTGATTCTATTTAAACATCCCA

Homo_sapiens --------------------------------------------------

Gorilla_gorilla TATTTGGGAAGAGCGCATAGGATAAAGAAAGAGATATCTCCACCTATAAA

Otolemur_garnettii TATTTGGGAAGAGCGCATAGGATAAAGAAAGAGATATCTCCACCTATAAA

Mus_musculus TATTTGGGAAGAGCGCATAGGATAAAGAAAGAGATATCTCCACCTATAAA

Rattus_norvegicus TATTTGGGAAGAGCGCATAGGATAAAGAAAGAGATATCTCCACCTATAAA

Jaculus_jaculus -------------------------------AGATATCTCCACCTATAAA

Cavia_porcellus TATTTGGGAAGAGCGCATAGGATAAAGAAAGAGATATCTCCACCTATAAA

Ochotona_princeps TATTTGGGAAGAGCGCATAGGATAAAGAAAGAGATATCTCCACCTATAAA

Sorex_araneus ---------------CATAGGATAAAGAAAGAGATATCTCCACCTATAAA

Sus_scrofa TATTTGGGAAGAGCGCATAGGATAAAGAAAGAGATATCTCCACCTATAAA

Bos_taurus TATTTGGGAAGAGCGCATAGGATAAAGAAAGAGATATCTCCACCTATAAA

Tursiops_truncatus TATTTGGGAAGAGCGCATAGGATAAAGAAAGAGATATCTCCACCTATAAA

Canis_familiaris TATTTGGGAAGAGCGCATAGGATAAAGAAAGAGATATCTCCACCTATAAA

Loxodonta_africana TATTTGGGAAGAGCGCACAGGATAAAGAAAGAGATATCTCCACCTATAAA

Procavia_capensis TATTTGGGAAGAGCGCATAGGATAAAGAAAGAGATATCCCCACCTATAAA

Choloepus_didactylus --------------------------------------------------

Myrmecophaga_tridactyla --------------------------------------------------

Macropus_eugenii --------------------------------------------------

Sarcophilus_harrisii TATTTGGAAAGCGCGCATAGGATAAAGAAAGAGATATCTCCACCTATAAA

Anolis_carolinensis ------------------GGGAGAGGGAAAGAG-----------------

Xenopus_tropicalis TATTTGGGCAGAGCACATAGAATAAAGAAAGAGGAATCCCCAGCTATAAA

Homo_sapiens --------------------------------A-CCCCTCAACTTCAAAG

Gorilla_gorilla TTGTCCACTTTGGAGAAC--------AAAAAAA-CCACTCAACTTCAAAG

Otolemur_garnettii TTGTGCACTTTGGAGAAC---------AAAAAA--CCCTCAACTTCAAAG

Mus_musculus TTGTGCACTTTGGAGAAC---------AAAAAC-CCCCTCAACTTCAAAG

Rattus_norvegicus TTGTGCACTTTGGAGAAC---------AAAAAC-CCCCTCAACTTCAAAG

Jaculus_jaculus TTGTGCACTTTGGAGAACAAAAAAAAAAAAAAA-ACCCTCAACTTCAAAG

Cavia_porcellus TTGTGCACTTTGGAGAAC----------AAAAA-CCCCTCAACTTCAAAG

Ochotona_princeps TTGTGCACTTTGGAGAAC---------AAAAAAGCCCCTCAACTTCAAAG

Sorex_araneus TTGTGCACTTTGGAGAAC---------AAAAAA-CCCCTCAACTTCAAAG

Sus_scrofa TTGTGCACTTTGGAGAAC---------AAAAAA-CCCCTCAACTTCAAAG

Bos_taurus TTGTGCACTTTGGAGAAC---------AAAAAA-CCCCTCAACTTCAAAG

Tursiops_truncatus TTGTGCACTTTGGAGAAC--------AAAAAAA-CCCCTCAACTTCAAAG

Canis_familiaris TTGTGCACTTTGGAGAAC---------AAAAAA-CCCCTCAACTTCAAAG

Loxodonta_africana TTGTGCACTTTGGAGAAC---------AAAAAACCCCCTCAACTTCAAAG

Procavia_capensis TTGTGCACTTTGGAGAAC---------AAAAAA-CCCCTCAACTTCAAAG

Choloepus_didactylus --------------------------------------------------

Myrmecophaga_tridactyla --------------------------------------------------

Macropus_eugenii --------------------------------------------------

Sarcophilus_harrisii TTGTGCACTTTGGAGAAC---------AAAAAA-CCCCTCAACTTCAAAG

Anolis_carolinensis ----------------------------------------------AGAG

Xenopus_tropicalis TTCTGCACTTTAGAGAAC------------AAAACCCCTGAACTTCAAAG

Homo_sapiens AGTCACAAATCACCCTTAATCAAAAAGGGTGCAGAAA----TTTTTTTGG

Gorilla_gorilla AGTCACAAATCACCCTTAATCAAAAAGGGTGCAGAAA----TTTTTTTGG

Otolemur_garnettii AGTCACAAATCACCCTTAATCAAAAAGGGTGCAGAAA----TTTTTTTGG

Mus_musculus AGTCACAAATCACCCTTAATCAAAAAGGGTGCAGAAA---TTTTTTTTGG

Rattus_norvegicus AGTCACAAATCACCCTTAATCAAAAAGGGTGCAGAAA----TTTTTTTGG

Jaculus_jaculus AGTCACAAATCACCCTTAATCAAAAAGGGTGCAGAAA----TTTTTTTGG

Cavia_porcellus AGTCACAAATCACCCTTAATCAAAAAGGGTGCAGAAA---TTTTTTTTGG

Ochotona_princeps AGTCACAAATCACCCTTAATCAAAAAGGGTGCAGAAA---TTTTTTTTGG

Sorex_araneus AGTCACAAATCACCCTTAATCAAAAAGGGTGCAGAAA-TTTTTTTTTTGG

Sus_scrofa AGTCACAAATCACCCTTAATCAAAAAGGGTGCAGAAA---TTTTTTTTGG

Bos_taurus AGTCACAAATCACCCTTAATCAAAAAGGGTGCAGAAA---TTTTTTTTGG

Tursiops_truncatus AGTCACAAATCACCCTTAATCAAAAAGGGTGCAGAAA---TTTTTTTTGG

Canis_familiaris AGTCACAAATCACCCTTAATCAAAAAGGGTGCAGAAAATTTTTTTTTTGG

Loxodonta_africana AGTCACAAATCACCCTTAATCAAAAAGGGTGCAGAAA---TTTTTTTTGG

Procavia_capensis AGTCACAAATCACCCTTAATCAAAAAGGGTGCAGAAA---TTTTTTTTGG

Choloepus_didactylus --------------------------------------------------

Myrmecophaga_tridactyla --------------------------------------------------

Macropus_eugenii --------------------------------------------------

Sarcophilus_harrisii AGTCACAAATCACCCTTAATCAAAAAGGGTGCAGAAA----TTTTTTTCG

Anolis_carolinensis AGAGAAAGAGAAACTGTGGGGTGGGGTGGGGGGGAAG------------T

Xenopus_tropicalis GGCCACAAATCAACCATAATCAAAGGGTGAAAAAAAC----TAATAATAA

Homo_sapiens GCCCTCCCCGCCATGAGCTCCTACGTAGCCAATTCATTCTATAAGCAGAG

Gorilla_gorilla GCCCTCCCCGCCATGAGCTCCTACGTAGCCAATTCATTCTATAAGCAGAG

Otolemur_garnettii GCCCTCCCCGCCATGAGCTCCTACGTAGCCAATTCATTCTATAAGCAGAG

Mus_musculus GCCCTCCCCGCCATGAGCTCCTACGTAGCCAATTCATTCTATAAGCAGAG

Rattus_norvegicus GCCCTCCCCGCCATGAGCTCCTACGTAGCCAATTCATTCTATAAGCAGAG

Jaculus_jaculus GCCCTCCCCGCCATGAGCTCCTACGTAGCCAATTCATTCTATAAGCAGAG

Cavia_porcellus GCCCTCCCCGCCATGAGCTCCTACGTAACCAATTCATTCTATAAGCAGAG

Ochotona_princeps GCCCTCCCCGCCATGAGCTCCTACGTAGCCAATTCATTCTATAAGCAGAG

Sorex_araneus GCCCTCCCCGCCATGAGCTCCTACGTAGCCAATTCATTCTATAAGCAGAG

Sus_scrofa GCCCTCCCCGCCATGAGCTCCTACGTAGCCAATTCATTCTATAAGCAGAG

Bos_taurus GCCCTCCCCGCCATGAGCTCCTACGTAGCCAATTCATTCTATAAGCAGAG

Tursiops_truncatus GCCCTCCCCGCCATGAGCTCCTACGTAGCCAATTCATTCTATAAGCAGAG

Canis_familiaris GCCCTCCCCGCCATGAGCTCCTACGTAGCCAATTCATTCTATAAGCAGAG

Loxodonta_africana GCCCTCCCCGCCATGAGCTCCTACGTAGCCAATTCATTCTATAAGCAGAG

Procavia_capensis GCCCTCCCCGCCATGAGCTCCTACGTAGCCAATTCATTCTATAAGCAGAG

Choloepus_didactylus --------------------------------------------------

Myrmecophaga_tridactyla --------------------------------------------------

Macropus_eugenii ------------ATGAGCTCCTACGTAGCCAATTCATTCTATAAGCAAAG

Sarcophilus_harrisii TCCCTCCCCGCCATGAGCTCCTACGTAGCCAATTCATTCTATAAGCAAAG

Anolis_carolinensis ATAATTCCTGACATGAGTTCCTACGTAGCCAACTCCTTCTATAAGCAAAA

Xenopus_tropicalis CAATAATCAGCCATGAGTTCGTACGTAGCCAATTCATTCTATAAGCAAAG

Homo_sapiens CCCCAATATCCCTGCCTATAACATGCAAACTTGTGGGAACTATGGATCGG

Gorilla_gorilla CCCCAATATCCCTGCCTATAACGTGCAAACTTGTGGGAACTATGGATCGG

Otolemur_garnettii CCCCAATATCCCTGCCTATAACATGCAAACTTGTGGGAACTATGGATCGG

Mus_musculus CCCCAATATCCCTGCCTATAACATGCAAACTTGTGGGAACTATGGATCGG

Rattus_norvegicus CCCCAATATCCCTGCCTATAACATGCAAACTTGTGGGAACTATGGATCGG

Jaculus_jaculus CCCCAATATCCCTGCCTATACCATGCAAACTTGTGGGAACTATGGATCGG

Cavia_porcellus CCCCAATATCCCTGCCTATAACATGCAAACTTGTGGGAACTATGGATCGG

Ochotona_princeps CCCCAATATCCCTTCCTATAACATGCAAACTTGTGGGAACTATGGATCGG

Sorex_araneus CCCCAATATCCCTGCCTATAACATGCAAACTTGTGGGAACTATGGATCGG

Sus_scrofa CCCCAATATCCCTGCCTATAACATGCAAACTTGTGGGAACTATGGATCGG

Bos_taurus CCCCAATATCCCTGCCTATAACATGCAAACTTGTGGGAACTATGGATCGG

Tursiops_truncatus CCCCAATATCCCTGCCTATAACATGCAAACTTGTGGGAACTATGGATCGG

Canis_familiaris CCCCAATATCCCTGCCTATAACATGCAAACTTGTGGGAACTATGGATCGG

Loxodonta_africana CCCCAATATCCCTGCCTATAACATGCAAACTTGTGGGAACTATGGATCGG

Procavia_capensis CCCCAATATCCCTGCCTATAACATGCAAACTTGTGGGAACTATGGATCGG

Choloepus_didactylus --------------------------------------------------

Myrmecophaga_tridactyla --------------------------------------------------

Macropus_eugenii CCCTAATATTCCGGCCTATAGCATGCAAACTTGTGGGAACTATGGATCTG

Sarcophilus_harrisii CCCTAATATTCCTGCCTATAGCATGCAAACTTGTGGGAACTATGGATCTG

Anolis_carolinensis TCAAAATGTTCCTGCGTATTCCATGCAAAGTTATGGGAATTATGGATCTG

Xenopus_tropicalis TCAAAATGTTCCAGCCTATTCCATGCAAAGTTATGGGAATTATGGATCTG

Homo_sapiens CCTCAGAGGTGCAGGCATCCAGGTACTGCTACGGCGGATTGGACTTAAGC

Gorilla_gorilla CCTCAGAGGTGCAGGCATCCAGGTACTGCTACGGCGGATTGGACTTAAGC

Otolemur_garnettii CCTCAGAGGTGCAGGCATCCAGGTACTGCTACGGCGGATTGGACTTAAGC

Mus_musculus CCTCAGAGGTGCAGGCATCCAGGTACTGCTACGGCGGATTGGACTTAAGC

Rattus_norvegicus CCTCAGAGGTGCAGGCATCCAGGTACTGCTACGGCGGATTGGACTTAAGC

Jaculus_jaculus CCTCAGAGGTGCAGGCATCCAGGTACTGCTACGGCGGATTGGACTTAAGC

Cavia_porcellus CCTCAGAGGTGCAGGCATCCAGGTACTGCTACGGCGGATTGGATTTAAGC

Ochotona_princeps CCTCAGAGGTGCAGGCATCCAGGTACTGCTACGGTGGATTGGACTTAAGC

Sorex_araneus CCTCAGAGGTGCAGGCATCCAGGTACTGCTACGGCGGATTGGACTTAAGC

Sus_scrofa CCTCAGAGGTGCAGGCATCCAGGTACTGCTACGGCGGATTGGACTTAAGC

Bos_taurus CCTCAGAGGTGCAGGCATCCAGGTACTGCTACGGCGGATTGGACTTAAGC

Tursiops_truncatus CCTCAGAGGTGCAGGCATCCAGGTACTGCTACGGCGGATTGGACTTAAGC

Canis_familiaris CCGCAGAGGTGCAGGCATCCAGGTACTGCTACGGTGGATTGGACTTAAGC

Loxodonta_africana CCTCAGAGGTGCAGGCATCCAGGTACTGCTACGGCGGATTGGACTTAAGC

Procavia_capensis CCTCAGAGGTGCAGGCATCCAGGTACTGCTACGGCGGATTGGACTTAAGC

Choloepus_didactylus --------------------------------------------------

Myrmecophaga_tridactyla --------------------------------------------------

Macropus_eugenii TCTCAGAGGTGCAGCCATCCAGGTACTGCTACAGTGGATTGGACTTAAGC

Sarcophilus_harrisii TCTCAGAGGTGCAGCCATCCAGGTACTGCTACAGTGGATTGGACTTAAGC

Anolis_carolinensis TGTCTGAGATTCAGCCATCCAGGTATTGCTACAGTGGGCTGGATCTGAGC

Xenopus_tropicalis TATCCGAGGTCCCTTCATCCAGGTATTGCTACAGTGGGTTGGATCTGAGC

Homo_sapiens ATCACTTTCCCACCGCCTGCGCCTTCCAACTCTCTCCACGGGGTAGACAT

Gorilla_gorilla ATCACTTTCCCACCGCCTGCGCCTTCCAACTCTCTCCACGGGGTAGACAT

Otolemur_garnettii ATCACTTTCCCACCGCCTGCGCCTTCCAACTCTCTCCACGGGGTAGACAT

Mus_musculus ATCACTTTCCCACCGCCTGCGCCTTCCAACTCTCTCCACGGGGTAGACAT

Rattus_norvegicus ATCACTTTCCCACCGCCTGCGCCTTCCAACTCTCTCCACGGGGTAGACAT

Jaculus_jaculus ATCACTTTCCCACCGCCTGCGCCTTCCAACTCTCTCCACGGGGTAGACAT

Cavia_porcellus ATCACTTTCCCACCGCCTGCGCCTTCCAACTCTCTCCACGGGGTAGACAT

Ochotona_princeps ATCACTTTCCCACCGCCTGCGCCTTCCAACTCTCTCCACGGGGTAGACAT

Sorex_araneus ATCACTTTCCCACCGCCTGCGCCTTCCAACTCTCTCCACGGGGTAGACAT

Sus_scrofa ATCACTTTCCCACCGCCTGCGCCTTCCAACTCTCTCCACGGGGTAGACAT

Bos_taurus ATCACTTTCCCACCGCCTGCGCCTTCCAACTCTCTCCACGGGGTAGACAT

Tursiops_truncatus ATCACTTTCCCACCGCCTGCGCCTTCCAACTCTCTCCACGGGGTAGACAT

Canis_familiaris ATCACTTTCCCACCGCCTGCGCCTTCCAACTCTCTCCACGGGGTAGACAT

Loxodonta_africana ATCACTTTCCCACCGCCTGCGCCTTCCAACTCTCTCCACGGGGTAGACAT

Procavia_capensis ATCACTTTCCCACCGCCTGCGCCTTCCAACTCTCTCCACGGGGTAGACAT

Choloepus_didactylus -----TTTCCCACCGCCTGCGCCTTCCAACTCTCTCCACGGGGTAGACAT

Myrmecophaga_tridactyla --------------------------------------------------

Macropus_eugenii ATCACTTTCCCACCGCCTGCTCCTTCTAACTCTCTCCACGGGTTAGACAT

Sarcophilus_harrisii ATCACTTTCCCACCGCCTGCTCCTTCCAACTCTCTTCACGGGTTAGACAT

Anolis_carolinensis ATTACATTCCCTCCATCTGGGGCTTCCAACTCTCTCAACGGTGTGGACAT

Xenopus_tropicalis ATTACATTCCCTTCTCCTGGTTCCTCTAACTCTCTGAGCGCTCTGGACAT

Homo_sapiens GGCTGCCAACCCCCGGGCTCACCCCGACCGCCCCGCCTGCAGCGCCGCGG

Gorilla_gorilla GGCTGCCAACCCCCGGGCTCACCCCGACCGCCCAGCCTGCAGCGCCGCGG

Otolemur_garnettii GGCTGCCAACCCCCGGGCTCACCCCGACCGCCCTGCCTGCAGCGCCGCGG

Mus_musculus GGCTGCCAACCCCCGGGCTCACCCCGACCGCCCCGCCTGCAGCGCCGCGG

Rattus_norvegicus GGCTGCCAACCCCCGGGCTCACCCCGACCGCCCCGCCTGCAGCGCTGCGG

Jaculus_jaculus GGCTGCCAAGCCCCGGGCTCACCCCGACCGCCCCGCCTGCAGCGCCGCA-

Cavia_porcellus GGCTGCCAACCCCCGGGCTCACCCGGACCGCCCCGCCTGCAGCGCTGCGG

Ochotona_princeps GGCTGCCAGCCCCCGGGCTCACCCCGAACGCCCCGCCTGCAGCGCCACGG

Sorex_araneus GGCTGCCAAGCCCCGGGCTCACCCCGACCGCCCCGCCTGCAGCGCCGCGG

Sus_scrofa GGCTGCCAACCCCCGGGCTCACCCCGACCGCCCCGCCTGCAGCGCCGCGG

Bos_taurus GGCTGCCAACCCCCGGGCTCACCCCGACCGCCCCGCCTGCAGCGCCGCAG

Tursiops_truncatus GGCTGCCAACCCCCGGGCTCACCCCGACCGCCCCGCCTGCAGCGCCGCGG

Canis_familiaris GGCAGCCAGCCCCCGGGCTCACCCCGACCGCCCCGCCTGCAGCGCCGCGG

Loxodonta_africana GGCTGCCAACCCCCGGGCTCACCCCGACCGCCCCGCCTGCAGCGCCGCTG

Procavia_capensis GGCTGCCAACCCCCGGGCTCACCCCGACCGCCCCGCCTGCAGCGCCACGG

Choloepus_didactylus GGCTGCCAACCCCCGGGCTCACCCCGACCGCCCCGCCTGCAGCGCTGCGG

Myrmecophaga_tridactyla --------------------------------------------------

Macropus_eugenii GGCTGCCACTCCTAGACCTCACCCAGACCGACCCGCCTGTACCACGGTGG

Sarcophilus_harrisii GGCTGCCACTCCTAGCCCTCACCCAGACCGACCCGCCTGTACCACGGTGG

Anolis_carolinensis GTCTTCAACCCCCAGACCTAACCCCGAGCGACCTTCTTGTACAGTCATGG

Xenopus_tropicalis GCCTTCAAACCCAAATGCTAACTCTGAGCGGCCTTCCTGCACTGTTATGG

Homo_sapiens CCGCTCCGGGACACGCTCCGGGCAGAGACGAAGCGGCTCCTCTGAACCCC

Gorilla_gorilla CCGCTCCGGGACACGCTCCGGGCAGAGACGAAGCGGCTCCTCTGAACCCC

Otolemur_garnettii CTGCTACGGGACACGCTCTGGGGAGAGACGAAGCGGCTCCTCTGAACCCT

Mus_musculus CCGCTCCGGGACACGCTCTGGGCAGAGACGAAGCGGCTCCTCTGAACCCT

Rattus_norvegicus CCGCTCCGGGACACGCTCTGGGCAGAGACGAAGCGGCTCCTCTGAACCCT

Jaculus_jaculus --GCTTCGGGACACGCTCTGGGCAGAGACGAAGCGGCTCCTCTGAACCCC

Cavia_porcellus CCGCTCCGGGACACGCTCTGGGCAGAGACGAAGCGGCTCCTCTGAACCCC

Ochotona_princeps CCGCTCCGGGACGCGCTCTGGGCAGAGACGAAGCGGCTTCTCTGAACCCC

Sorex_araneus CCGCTCCGGGACACGCTCTGGGCAGAGATGAAGCGGCTCCTCTGAACCCC

Sus_scrofa CCGCTCCGGGACACGCTCTGGGCAGAGATGAAGCGGCTCCTCTGAACCCC

Bos_taurus CCGCTCCGGGACACGCTCTGGGCAGAGATGAAGCGGCTCCTCTGAACCCC

Tursiops_truncatus CCGCTCCGGGACACGCTCTGGGCAGAGATGAAGCGGCTCCTCTGAACCCC

Canis_familiaris CCGCTCCGGGACACGCTCTGGGCAGAGACGAAGCGGCTCCTCTGAACCCC

Loxodonta_africana CCGCTCCGGGACACGCTTTGGGCAGAGATGAAGCGGCTCCTCTGAACCCC

Procavia_capensis CCGCTCCGGGACACGCTCTGGGCAGAGATGAAGCGGCTCCTCTGAACCCC

Choloepus_didactylus CCGCTCCGGGACACGCTCTGGGCAGAGACGAAGCGGCTCCTCTGAACCCC

Myrmecophaga_tridactyla --------------------------------------------------

Macropus_eugenii CAGCTCCGGGACACGCTCTGGGCAGAGACGAACCGGCTCCTCTAAACCCC

Sarcophilus_harrisii CAGCTCCGGGACACGCTCTGGGCAGAGACGAACCGGCTCCTCTAAACCCC

Anolis_carolinensis GATCTTCAGGGCACACTTTAGGCAGAGACGACCAGGCTTCTCTGAATCCA

Xenopus_tropicalis GTTCTTCTGGGCACACTGTGGGAAGAGGAGAGCAGAGTGCACTCAACTCT

Homo_sapiens GGGATGTACAGTCAGAAGGCGG----------------------------

Gorilla_gorilla GGGATGTACAGTCAGAAGGCGG----------------------------

Otolemur_garnettii GGGATGTACAATCAGAAGGCGG----------------------------

Mus_musculus GGGATGTACAGTCAGAAGGCGG----------------------------

Rattus_norvegicus GGGATGTACAGTCAGAAGGCGG----------------------------

Jaculus_jaculus GGGATGTACAATCAGAAGGCGG----------------------------

Cavia_porcellus GGGATGTACAGTCAGAAGGCGG----------------------------

Ochotona_princeps GGGATGTACAGCCAGAAGGCGG----------------------------

Sorex_araneus GGGATGTACAGTCAGAAGGCGG----------------------------

Sus_scrofa GGGATGTACAGTCAGAAGGCGG----------------------------

Bos_taurus GGGATGTACAGTCAGAAGGCGG----------------------------

Tursiops_truncatus GGGATGTACAGTCAGAAGGCGG----------------------------

Canis_familiaris GGGATGTACAGTCAGAAGGCGG----------------------------

Loxodonta_africana GGGATGTACAGTCAGAAGGCGG----------------------------

Procavia_capensis GGGATGTACAGTCAGAAGGCAG----------------------------

Choloepus_didactylus GGGATGTACAGTCAGAAGGCGG----------------------------

Myrmecophaga_tridactyla --------------------------------------------------

Macropus_eugenii GGAATGTACAATCAGAAGGCGG----------------------------

Sarcophilus_harrisii GGGATGTACAATCAGAAGGCGG----------------------------

Anolis_carolinensis GGAATTTACAGTCAGAAAGCTGGTGGGAACAGCAGCAGCAGCAACAACAG

Xenopus_tropicalis GGGATTTACAATCAGAAAGCTG----------------------------

Homo_sapiens --------------CTCGCCCGGCGCTGGAGGAGCGAGCTAAGAGCAGTG

Gorilla_gorilla --------------CTCGCCCGGCGCTGGAGGAGCGAGCTAAGAGCAGTG

Otolemur_garnettii --------------CTCGCCCGGCGCTGGAGGAGCGAGCTAAGAGCAGTG

Mus_musculus --------------CTCGCCCGGCGCTGGAGGAGCGAGCTAAGAGCAGTG

Rattus_norvegicus --------------CCCGCCCGGCGCTGGAGGAGCGAGCTAAGAGCAGTG

Jaculus_jaculus --------------CTCGGCCGGCGCCGGAGGAGCGAGCTCAGAGCGGTG

Cavia_porcellus --------------CTCGCCCGGCGCTGGAGGAGCGAGCTAAGAGCAGAG

Ochotona_princeps --------------CCCGCCCGGCGCTGGAGGAGCGAGCTGCGAGCGCTG

Sorex_araneus --------------CTCGCCCAGCGCTGGAGGAGCGAGCTAAGAGCGGTG

Sus_scrofa --------------CTCGCCCAGCGCTGGAGGAGCGAGCTAAGAGCAGTG

Bos_taurus --------------CTCGCCCAGCGCTGGAGGAGCGAGCTAAGAGCAGTG

Tursiops_truncatus --------------CTCGCCCAGCGCTGGAGGAGCGAGCTAAGAGCAGTG

Canis_familiaris --------------CTCGCCCAGCGCCGGAGGAGCGAGCTAGGAGCACTG

Loxodonta_africana --------------CTCGCCCGGCGCTGGAGGACCGAGCTAAGAGCAGTG

Procavia_capensis --------------CTCGCCCGGCGCTGGAGGAGCGAGCTAAGAGCAGTG

Choloepus_didactylus --------------CTCGCCCAGCGCTGGAGGAGCGAGCTAAGAGCAGTG

Myrmecophaga_tridactyla --------------------------------------------------

Macropus_eugenii --------------CTCGCCCGGCGCTGGAGGACAGATCTAAGGGGAGTG

Sarcophilus_harrisii --------------CTCGCCCGGCGCTGGAGGACAGATCTAAGGGGAGTG

Anolis_carolinensis CCACCCCAACAACCCCAACACGCCGTTGGAAGATAGATCTAAGAGCAGTG

Xenopus_tropicalis --------------CGACTACATCGCTGGAGGAGAGATCTAAAGGGATTG

Homo_sapiens GGGAGATCAAAGAGGAGCAGGCGCAGACAGGGCAGCCCGCCGGACTGAGC

Gorilla_gorilla GGGAGATCAAAGAGGAGCAGGCGCAGACAGGGCAGCCCGCCGGACTGAGC

Otolemur_garnettii GGGAGATCAAAGAGGAGCAGGCGCAGACAGGGCAGCCCGCCGGACTGAGC

Mus_musculus GGGAGATCAAAGAGGAGCAGGCGCAGACAGGGCAGCCTGCCGGACTGAGC

Rattus_norvegicus GGGAGATCAAAGAGGAGCAGGCGCAGACAGGGCAGCCTGCCGGACTGAGC

Jaculus_jaculus GGGAGATCAAAGAGGAGCAGGCGCAGACAGGGCCAGCCGCCGGACTGAGC

Cavia_porcellus GGGAGATCAAAGAGGAGCAGGCGCAGACAGGGCAACCCGCCGGACTGAGC

Ochotona_princeps GGCAGATCAAAGAGGAGCAGGCGCAGCCTGGGCAGCCCGCAGCGCGGAGC

Sorex_araneus GGGAGATCAAAGAGGAGCAGGCGCAGACAGGGCAGCCCGCCGGACTGAGC

Sus_scrofa GGGAGATCAAAGAGGAGCAGGCGCAGACAGGGCAGCCCGCCGGACTGAGC

Bos_taurus GGGAGATCAAAGAGGAGCAGGCGCAGACAGGGCAGCCTGCCGGACTGAGC

Tursiops_truncatus GGGAGATCAAAGAGGAGCAGGCGCAGACAGGGCAGCCCGCCGGACTGAGC

Canis_familiaris GGGAGATCAAAGAGGAGCAGGCGCAGACAGGGCAGCCCGCCGCCCGGAGC

Loxodonta_africana GGGAAATCAAAGAGGAGCAGGCGCAGACAGGGCAGCCCGCCGGACTGAGC

Procavia_capensis GGGAGATCAAAGAGGAGCAGGCGCAGACAGGGCAGCCCGCCGGACTGAGC

Choloepus_didactylus GGGAGATCAAAGAGGAGCAGGCGCAGACAGGGCAGCCCGCCGGACTGAGC

Myrmecophaga_tridactyla --------------------------------------------------

Macropus_eugenii GGGAGATCAAGGAAGAACAGGCGCAGACTGGGCAGCCCGCAGGACTGAGC

Sarcophilus_harrisii GGGAGATCAAGGAAGAACAGGCTCAGACTGGGCAGCCCGCAGGACTGAGC

Anolis_carolinensis GGGAAATCAAGAGTGAGCCGGTGCAAACAGCCCAGCAAAGCGGGCAGTCC

Xenopus_tropicalis AGAGCATCAAAACTGAGCCGGCACAGAGCACTCCACAAGGTGGGCAACCC

Homo_sapiens --------------------------------------------------

Gorilla_gorilla --------------------------------------------------

Otolemur_garnettii --------------------------------------------------

Mus_musculus --------------------------------------------------

Rattus_norvegicus --------------------------------------------------

Jaculus_jaculus --------------------------------------------------

Cavia_porcellus --------------------------------------------------

Ochotona_princeps --------------------------------------------------

Sorex_araneus --------------------------------------------------

Sus_scrofa --------------------------------------------------

Bos_taurus --------------------------------------------------

Tursiops_truncatus --------------------------------------------------

Canis_familiaris --------------------------------------------------

Loxodonta_africana --------------------------------------------------

Procavia_capensis --------------------------------------------------

Choloepus_didactylus --------------------------------------------------

Myrmecophaga_tridactyla --------------------------------------------------

Macropus_eugenii --------------------------------------------------

Sarcophilus_harrisii --------------------------------------------------

Anolis_carolinensis CTGCAGCAGCCACAGCACCACCAACACCAACAACACCACCAACACCATCA

Xenopus_tropicalis --------------------------------------------------

Homo_sapiens ----------------------CAGCCACCGGCCCCGCCACAGATTTACC

Gorilla_gorilla ----------------------CAGCCAGCGGCCCCGCCACAGATTTACC

Otolemur_garnettii ----------------------CAGCCACCGGCCCCGCCACAGATTTACC

Mus_musculus ----------------------CAGCCACCGGCCCCGCCACAGATTTACC

Rattus_norvegicus ----------------------CAGCCACCGGCCCCGCCACAGATTTACC

Jaculus_jaculus ----------------------CAGCCACCGGCCCCGCCACAGATTTACC

Cavia_porcellus ----------------------CAGCCACCGGCCCCGCCACAGATTTACC

Ochotona_princeps ----------------------CAGCCGCCGGCCCCGCCACAGATTTACC

Sorex_araneus ----------------------CAGCCACCGGCTCCGCCACAGATTTACC

Sus_scrofa ----------------------CAGCCACCGGCCCCGCCACAGATTTACC

Bos_taurus ----------------------CAGCCACCGGCCGCGCCACAGATTTACC

Tursiops_truncatus ----------------------CAGCCACCGGCCCCGCCACAGATTTACC

Canis_familiaris ----------------------CAGCCACCGGCCCCGCCACAGATTTACC

Loxodonta_africana ----------------------CAGCCACCGGCCCCGCCACAGATTTACC

Procavia_capensis ----------------------CAGCCACCGGTCCCGCCACAGATATACC

Choloepus_didactylus ----------------------CAGCCACCGGCCCCGCCACAGATTTACC

Myrmecophaga_tridactyla --------------------------------------------------

Macropus_eugenii ----------------------CAGCCACCGGCCCCGCCACAAATATACC

Sarcophilus_harrisii ----------------------CAGCCACCGGCCCCGCCACAAATATACC

Anolis_carolinensis CCACCAGCAGCAGCAACAACAGCAGCAACAGCAGCCACCGCAAATATATC

Xenopus_tropicalis ----------------CAGCAGCAGCAGCAGCAGCCACCACAAATATATC

Homo_sapiens CGTGGATGACCAAACTGCACATGAGCCACGGT---AAACTTTAGGACTTC

Gorilla_gorilla CGTGGATGACCAAACTGCACATGAGCCACGGT---AAACTTTAGGACTTC

Otolemur_garnettii CGTGGATGACCAAACTGCACATGAGCCACGGT---AAACTTTAGGACTTC

Mus_musculus CGTGGATGACCAAACTGCACATGAGCCACGGT---AAACTTTAGGACTTC

Rattus_norvegicus CGTGGATGACCAAACTGCACATGAGCCACGGT---AAACTTTAGGACTTC

Jaculus_jaculus CGTGGATGACCAAACTGCACATGAGCCACGGT---AAACTTTAGGACTTC

Cavia_porcellus CGTGGATGACCAAACTGCACATGAGCCACGGT---AAACTTTAGGACTTC

Ochotona_princeps CGTGGATGACCAAACTGCACATGAGCCACGGTAAAAAACTTTAGGACTTC

Sorex_araneus CGTGGATGACCAAACTGCACATGAGCCACGGT---AAACTTTAGGACTTC

Sus_scrofa CGTGGATGACCAAACTGCACATGAGCCACGGT---AAACTTTAGGACTTC

Bos_taurus CGTGGATGACCAAACTGCACATGAGCCACGGT---AAACTTTAGGACTTC

Tursiops_truncatus CGTGGATGACCAAACTGCACATGAGCCACGGT---AAACTTTAGGACTTC

Canis_familiaris CGTGGATGACCAAACTGCACATGAGCCACGGT---AAACTTTAGGACTTC

Loxodonta_africana CGTGGATGACCAAACTGCACATGAGCCACGGT---AAACTTTAGGACTTC

Procavia_capensis CGTGGATGACCAAACTGCACATGAGCCACGGT---AAACTTTAGGACTTC

Choloepus_didactylus CGTGGATGACCAAACTGCACATGAGCCACGGT---AAACTTTAGGACTTC

Myrmecophaga_tridactyla --------------------------------------------------

Macropus_eugenii CGTGGATGACCAAACTCCACATGAGCCACGGT---AAACTTTAGGGCTTC

Sarcophilus_harrisii CGTGGATGACCAAACTCCACATGAGCCACGGT---AAACTTTAGGGCTTC

Anolis_carolinensis CGTGGATGACCAAACTACACATGAGCCACGGT---AAACTTTCCCTTCTC

Xenopus_tropicalis CTTGGATGACCAAACTGCACATGAGCCATGGT---AAACTTTCACTTCTT

Homo_sapiens ATTTTGCGCTCT-CGGGTCCGCC--TGGGTTTTATAGGCCATGCGGGGC-

Gorilla_gorilla ATTTTGCGCGCT-CGGGTCCGCC--TGGGTTTTATAGGCCATGCGGGGC-

Otolemur_garnettii ATTTTGCGCGCTAAGGGTCCGCC--TGAGTTTTATAGGC-ATGCGGGGCA

Mus_musculus ATTTTGCGCGCT-CGGGTCCGCC--TGGGTTTTATAGGCCATGCGGGGC-

Rattus_norvegicus ATTTTGCGCGCT-CGGGTCCGCC--TGGGTTTTATAGGCCATGCGGGGC-

Jaculus_jaculus ATTTTGCGCGCT-CGGGTCCGCC--TGGGTTTTATAGGCCATGCGGGGC-

Cavia_porcellus ATTTTGCGCGCT-CGGGTCCGCC--TGGGTTTTATAGGCCATGCGGGGC-

Ochotona_princeps ATTTTGCGCGCT-CGGGTCCGCC--TGGGTTTTATAGGCCATGCGGGGC-

Sorex_araneus ATTTTGCGCGCT-CGGGTCCGCC--TGGGTTTTATAGGCCATGCGGGGC-

Sus_scrofa ATTTTGCGCGCT-CGGGTCCGCC--TGGGTTTTATAGGCCATGCGGGGC-

Bos_taurus ATTTTGCGCGCT-CGGGTCCGCC--TGGGTTTTATAGGCCATGCGGGGC-

Tursiops_truncatus ATTTTGCGCGCT-CGGGTCCGCC--TGGGTTTTATAGGCCATGCGGAGCA

Canis_familiaris ATTTTGCGCGCT-CGGGTCCGCC--TGGGTTTTATAGGCCATGCGGGGC-

Loxodonta_africana ATTTTGCGCGCT-CGGGTCCGCC--TGGGTTTTATAGGCCATGCGGGGC-

Procavia_capensis ATTTTGCGCGCT-CGGGTCCGCC--TGGGTTTTATAGGCCATGCGGGGC-

Choloepus_didactylus ATTTTGCGCGCT-CGGGTCCGCC--TGGGTTTTATAGGCCATGCGGGGC-

Myrmecophaga_tridactyla --------------------------------------------------

Macropus_eugenii ATTTTGTGCGCT-CGGGTCCGCC--TGGGTTTTATAGGCCATACGGGGC-

Sarcophilus_harrisii ATTTTGTGCGCT-CGGGTCCGCC--TGGGTTTTATAGGCCATACGGGGC-

Anolis_carolinensis GATTTTGTTTCACAGGATTTGCC--TGGGTTTTATAGGCCATACGGGGC-

Xenopus_tropicalis GATTTTGTTTCATAGGCTTTTGCATTGGGTTTTATAGGCCATACGGGGC-

Homo_sapiens AAATAA----AG-AAAAAAAACCTGCGGCCATAAATTTTACGATCCAGGC

Gorilla_gorilla AAATAA----AG-AAAAAAAACCTGCGGCCATAAATTTTACGATCCAGGC

Otolemur_garnettii ACCTCT----AG-TGCAAAAACCTGCGGCCATAAATTTTACGATC--AGC

Mus_musculus AAATAA----AG-AAAAAAAACCTGCGGCCATAAATTTTACGATCCAGGC

Rattus_norvegicus AAATAA----AGAAAAAAAAACCTGCGGCCATAAATTTTACGATCCAGGC

Jaculus_jaculus AAATAA----AGAAAAAAAAACCTGCGGCCATAAATTTTACGATCCAGGC

Cavia_porcellus AAATAA----AG-AAAAAAAACCTGCGGCCATAAATTTTACGATCCAGGC

Ochotona_princeps AAATAA----AGAAAAAAAAACCTGCGGCCATAAATTTTACGATCCAGGC

Sorex_araneus AAATAA----AG-AAAAAAAACCTGCGGCCATAAATTTTACGATCCAGGC

Sus_scrofa AAATAA----AG-AAAAAAAACCTGCGGCCATAAATTTTACGATCCAGGC

Bos_taurus AAATAA----AG-AAAAAAAACCTGCGGCCATAAATTTTACGATCCAGGC

Tursiops_truncatus AAATAA----AG-AAAAAAAACCTGCGGCCATAAATTTTACGATACAGGC

Canis_familiaris AAATAA----AGAAAAAAAAACCTGCGGCCATAAATTTTACGATCCAGGC

Loxodonta_africana AAATAA----AG-AAAAAAAACCTGCGGCCATAAATTTTACGATCCAGGC

Procavia_capensis AAATAA----AG--AAAAAAACCTGCGG-CATAAATTTAACGATCCAGGC

Choloepus_didactylus AAATAA----AGAAAAAAAAACCTGCGGCCATAAATTTTACGATCCAGGC

Myrmecophaga_tridactyla --------------------------------------------------

Macropus_eugenii AAATAA----AA-AGAAAAAACCTGCGGCCATAAATTTTACGGTGCAGGC

Sarcophilus_harrisii AAATAA----AA-AGAAAAAACCTGCGGCCATAAATTTTACGGTGCAGGC

Anolis_carolinensis AAATAATAAGGGGGAAAAAAACCTGGGGTCGTAAATTTTACGACTAAGGC

Xenopus_tropicalis AAATAA--------TAAAAAACCTGGGGTCGTAAATTTTACGACTAAGGC

Homo_sapiens ATCAATGGCTCGTAAAACTGTCCACTAAAAGGCTTAGAGGCTGTG-TGCG

Gorilla_gorilla ATCAATGGCTCGTAAAACTGTCCACTAAAAGGCTTAGAGGCTGTG-TGCG

Otolemur_garnettii ATCA--TGCTCGTAA---CTGTCACTTAAAGGCTTAGA-GCTGTG-TGCG

Mus_musculus ATCAATGGCTCGTAAAACTGTCCACTAAAAGGCTTAGAGGCTGTG-TGCG

Rattus_norvegicus ATCAATGGCTCGTAAAACTGTCCACTAAAAGGCTTAGAGGCTGTG-TGCG

Jaculus_jaculus ATCAATGGCTCGTAAAACTGTCCACTAAAAGGCTTAGAGGCTGTG-TGCG

Cavia_porcellus ATCAATGGCTCGTAAAACTGTCCACTAAAAGGCTTAGAGGCTGTG-TGCG

Ochotona_princeps ATCAATGGCTCGTAAAACTGTCCACTAAAAGGCTTAGAGGCTGTG-TGCG

Sorex_araneus ATCAATGGCTCGTAAAACTGTCCACTAAAAGGCTTAGAGGCTGTG-TGCG

Sus_scrofa ATCAATGGCTCGTAAAACTGTCCACTAAAAGGCTAGAAGGCTGTG-TGCG

Bos_taurus ATCAATGGCTCGTAAAACTGTCCACTAAAAGGCTTAGAGGCTGTG-TGCG

Tursiops_truncatus ATCAATGGCTCGTAAAACTGTCCACTAAAAGGCTTAGAGGCTGTG-TGCG

Canis_familiaris ATCAATGGCTCGTAAAACTGTCCACTAAAAGGCTTAGAGGCTGTG-TGCG

Loxodonta_africana ATCAATGGCTCGTAAAACTGTCCACTAAAAGGCTTAGAGGCTGTG-TGCG

Procavia_capensis ATCAATGGCTCGTAAAACTGTCCACTAAAAGGCTTAGAGGCTGTGTTGCG

Choloepus_didactylus ATCAATGGCTCGTAAAACTGTCCACTAAAAGGCTTAGAGGCTGTG-TGCG

Myrmecophaga_tridactyla --------------------------------------------------

Macropus_eugenii ATCAATTGCTCGTAAAACTGTCCACTAAAAGGCTTAGAGGCTGTA-TGCG

Sarcophilus_harrisii ATCAATTGCTCGTAAAACTGTCCACTAAAAGGCTTAGAGGCTGTA-TGCG

Anolis_carolinensis ATCAATTGCTCGTAAAACTGTCCACTAAAAGGCTTAGAGGCTATA-GACG

Xenopus_tropicalis ATCAATTGCTCGTAAAACTGTCCACTAAAAGGCTTAGAGGCTATA-GACG

Homo_sapiens CCCAAATTTACGACG-ACATAATTGGATCATA-GGAACAAAACGTGT---

Gorilla_gorilla CCCAAATTTACGACG-ACATAATTGGATCATA-GGAACAAAACGTGT---

Otolemur_garnettii CCC-AATTTACGACG-ACATA--TTGATCATA-GCA--CAAACGTGT---

Mus_musculus CCCAAATTTACGACG-ACATAATTGGATCATA-GGAACAAAACGTGT---

Rattus_norvegicus CCCAAATTTACGACG-ACATAATTGGATCATA-GGAACAAAACGTGT---

Jaculus_jaculus CCCAAATTTACGACG-ACATAATTGGATCATA-GGAACAAAACGTGT---

Cavia_porcellus CCCAAATTTACGACG-ACATAATTGGATCATA-GGAACAAAACGTGT---

Ochotona_princeps CCCAAATTTACGACG-ACATAATTGGATCATA-GGAACAAAACGTGT---

Sorex_araneus CCCAAATTTACGACG-ACATAATTGGATCATA-GGAACAAAACGTGT---

Sus_scrofa CCCAAATTTACGACGAACATAATTGGATCATA-GGAACAAAACGTGT---

Bos_taurus CCCAAATTTACGACG-ACATAATTGGATCATA-GGAACAAAACGTGT---

Tursiops_truncatus CCCAAATTTACGACG-ACATAATTGGATCATA-GGAACAAAACGTGT---

Canis_familiaris CCCAAATTTACGACG-ACATAATTGGATCATA-GGAACAAAACGTGT---

Loxodonta_africana CCCAAATTTACGACG-ACATAATTGGATCATA-GGAACAAAACGTGT---

Procavia_capensis CCCAGAGTTACGAGG-GCATAATTGGGTCATAGGGAACAAAACGTGT---

Choloepus_didactylus CCCAAATTTACGACG-ACATAATTGGATCATA-GGAACAAAACGTGT---

Myrmecophaga_tridactyla --------------------------------------------------

Macropus_eugenii CCCAAATTTACGACG-ACATAATTGGATCATA-GGAACAAAACGTGT---

Sarcophilus_harrisii CCCAAATTTACGACG-ACATAATTGGATCATA-GGAACAAAACGTGT---

Anolis_carolinensis CCCAAATTTATGACG-GCATAATTGGATCATA-GAAACAAAACGGGT---

Xenopus_tropicalis CTCAAATTTATGACA-GCATAATTGGATCATA-GGAACACAACGCGTAGG

Homo_sapiens ATAAAAGGCAATATTCAATTTTTGGGGGAGAGGGAGGGAGTTAAAAAAAT

Gorilla_gorilla ATAAAAGGCAATATTCAATTTTTGGGGGAGAGGGAGGGAGTTAAAAAAAT

Otolemur_garnettii ATAAAAGCATA---TTCATTTTTGGGGGAGAGGGAGGGAGTTAAAAAAAT

Mus_musculus ATAAAAGGCAATATTCAATTTTGGGGGGAGAGGGAGGGAGTTAAAAAAAT

Rattus_norvegicus ATAAAAGGCAATATTCAATTTTGGGGGGAGAGGGAGGGAGTTAAAAAAAT

Jaculus_jaculus ATAAAAGGCAATATTCAATTTTTGGGGGAGAGGGAGGGAGTTAAAAAAAT

Cavia_porcellus ATAAAAGGCAATATTCAATTTTTGGGGGAGAGGGAGGGAGTTAAAAAAAT

Ochotona_princeps ATAAAAGGCAATATTCAATTTTGGGGGGAGAGGGAGGGAGTTAAAAAAAT

Sorex_araneus ATAAAAGGCAATATTCAATTTTTGGGGGAGAGGGAGGGAGTTAAAAAAAT

Sus_scrofa ATAAAAGGCAATATTCAATTTTTGGGGGAGAGGGAGGGAGTTAAAAAAAT

Bos_taurus ATAAAAGGCAATATTCAATTTTTGGGGGAGAGGGAGGGAGTTAAAAAAAT

Tursiops_truncatus ATAAAAGGCAATATTCAATTTTGGGGGGAGAGGGAGGGAGTTAAAAAAAT

Canis_familiaris ATAAAAGGCAATATTCAATTTTTGGGGGAGAGGGAGGGAGTTAAAAAAAT

Loxodonta_africana ATAAAAGGCAATATTCAATTTTTGGGGGAGAGGGAGGGAGTTAAAAAAAT

Procavia_capensis AT-AAAGGCAATATTCAGTTGTGGGGGGAGAGGGAGGGAGTTAAAAGAAT

Choloepus_didactylus ATAAAAGGCAATATTCAATTTTTGGGGGAGANNNNNNNNNNNNNNNNNNN

Myrmecophaga_tridactyla --------------------------------------------------

Macropus_eugenii ATAAAAGGCAATATTCAATTTTTGGGGGGGAGGGAGGGAGTTAAAAAAAC

Sarcophilus_harrisii ATAAAAGGCAATATTCAATTTTTGGGGGGGAGGGAGGGAGTTAAAAAAAT

Anolis_carolinensis AT-AAAGGCAATATTCCATTTTTGGGGGAGAGAGTGGGGGAGAAGGAAAA

Xenopus_tropicalis ATAGACGGTAATTCCACATTTTTTGGAAGGAGACAGGAAAAAAAAAAAAA

Homo_sapiens AGAGGGATCTGAAGGG----------TGAGGA---GCGCGGGG--CTCC-

Gorilla_gorilla AGAGGGATCTGAAGGG----------TGAGGA---GCGCGGGG--CTCC-

Otolemur_garnettii AGAGGGATCTGTAGAG----------TGAGGA---GCGCGGGG--CTCC-

Mus_musculus AGAGGGATCTGAAGGG----------TGAGGA---GCATGGGG--CTCC-

Rattus_norvegicus AGAGGGATCTGAAGGG----------TGAGGA---GCATGGGG--CTCC-

Jaculus_jaculus AGAGGGATGTGAAGGG----------TGAGGAGGGGCGCGAGG--CTCC-

Cavia_porcellus AGAGGGATCTGAAGGG----------TGAGGA---GCGCGGGG--CTCC-

Ochotona_princeps AGAGGGATCTGAAGGG----------TGAGCG---GCGCGGGGGTCTCC-

Sorex_araneus AGAGGGATCTGAAGGG----------TGAGCT---GCGCGGGG--CTCC-

Sus_scrofa AGAGGGATCTGAAGGG----------TGAGGA---GCGCGGGG--CTCC-

Bos_taurus AGAGGGATCTGAAGGG----------TGAGGA---GCGCGGGG--CTCC-

Tursiops_truncatus AGAGGGATCTGAAGGG----------TGAGGA---GCGCGGGG--CTCCA

Canis_familiaris AGAGGGATCTGAAGGG----------TGAGGA---GCGCGGGG--CTGC-

Loxodonta_africana AGAGGGATCTGAAGGG----------TGAGGA---ATGCGGGG--CTCC-

Procavia_capensis AGAGGGATCTGAAGGG----------TGAGGA---ATGCGGGG--CTCC-

Choloepus_didactylus NNNNNNNNNNNNNNNN----------NNNNNN---NNNNNNNN--NNNN-

Myrmecophaga_tridactyla --------------------------------------------------

Macropus_eugenii ATAGGGAACTGAGGGG----------TAAGGC---AAACTGGC--CTTAC

Sarcophilus_harrisii ATAGGGATCTGAGGGG----------TAAGGT---ACACCGGC--CTTAT

Anolis_carolinensis AGATTTTTTAAGAGGGAGAAAAAACTTGGAAATCCGCATAAGG---TTTA

Xenopus_tropicalis GAATGAAAAAATAAAATTACAACTTTTATATTTTTATTTG-----CTCTT

Homo_sapiens AGAGCGGGGAT-CCCCCCGCGG--C--------------------TCCCT

Gorilla_gorilla AGAGCGGGGAT-CCCCCCGCGG--C--------------------TCCCT

Otolemur_garnettii AGAGCGGGGAT-CCCCCCGCGGCTC--------------------TCTCT

Mus_musculus AGAGTGGGGAT-CCCCCCGCGG--C--------------------TCCCT

Rattus_norvegicus AGAGTGGGGAT-CCCCCCGCGG--------------------------CT

Jaculus_jaculus AGAGCGGGGAT-CCCCCCGCGG--C--------------------TCCCA

Cavia_porcellus AGAGCGGGGAT-CCCCCCGCGG--C--------------------T-CCC

Ochotona_princeps GGCGCGGGGAT-CCCCCCGCGGCTCACCCCGCTCGCTCGCGCGCTCCCCT

Sorex_araneus AGAGCGGGGAT-CCCCCCGCGG--C--------------------TCCCT

Sus_scrofa AGAGCGGGGAT-CCCCCCGCGG------------------------TCCC

Bos_taurus AGAGCGGGGAT-CCCCCCGCGG------------------------TCCC

Tursiops_truncatus AGAGCGGGGAT-CCCCCCGCGG------------------------TCCC

Canis_familiaris AGAGCGGGGATCCCCCCCGAGG--C--------------------TCCCT

Loxodonta_africana AGAGCGGGGTT-CCCCCCGCGG--C--------------------TCCCT

Procavia_capensis AGAGCGGGGAT-CCCCCCGCGG--C--------------------TCCCT

Choloepus_didactylus NNNNNNNNNNN-NNNNNNNNNN--N--------------------NNNNT

Myrmecophaga_tridactyla -----------------------------------------------CCT

Macropus_eugenii AGAGAGGGGAT-CCCCTTTTCA--C--------------------CATCA

Sarcophilus_harrisii AGAGAGGGGATCCCCCCCTTCA--G--------------------CATCA

Anolis_carolinensis ATAGGAAGGAG-CTGGGGGTTTGTGTTGGGGATTTTTAAAAAATCGTTCC

Xenopus_tropicalis ACTGCTAGGAA---------------------------------------

Homo_sapiens CCCTC-----------------CCTCCCCTGCGGAGCCGGCTCCGC----

Gorilla_gorilla CCCTC-----------------CCTCCCCTGCGGAGCCGGCTCCGC----

Otolemur_garnettii CCTCC-----------------CTCTCCCTGCGGAGCCGGCTCGGCCGTC

Mus_musculus CCCTC-----------------CCTCCCCTGCGGAGCCGGCTCGGC----

Rattus_norvegicus CCCTC-----------------CCTCCCCTGCGGAGCCGGCTCGGC----

Jaculus_jaculus CCCTC-----------------CCTCCCCTGCGGAGCCGGCTCGGC----

Cavia_porcellus TCCTC-----------------CCTCCCCTGCGGAGCCGGCTCGGC----

Ochotona_princeps CCCGC-----------------CCTCCCCAGCGGACCGAGCCCGCTCGGC

Sorex_araneus CCCT------------------CCTCCCGCGTGGAGCCGGCTCGGC----

Sus_scrofa TCCTC-----------------CCTCCCCTGCGGAGCCGGCTCGGC----

Bos_taurus TCCTC-----------------CCTCCCCTGCGGAGCCGGCTCGGC----

Tursiops_truncatus TCCTC-----------------CCTCCCCTGCGGAGCCGGCTCGGC----

Canis_familiaris CCCTC-----------------CCTCCCCCGTGCAGCCGG--CGCC----

Loxodonta_africana CCCTC-----------------CCTCCCCTGTGGAGCCGGCTCGGC----

Procavia_capensis CCCTC-----------------CCTGTCCTGCGTAGCCGGCTCGGC----

Choloepus_didactylus CCCTC-----------------CCTCCCCTGCGGAGCCGGCTCGGC----

Myrmecophaga_tridactyla CCCTC-----------------CCTCCCCTGTGGAGCCGGCTCGGC----

Macropus_eugenii CCCCCATTATTAGGGAAATGATTCTCATTT--GGGGGGGGATTGAG----

Sarcophilus_harrisii CCCCCATTATTAGGGAAATGATTCTCATTTGGGGGGGGGGATTGAG----

Anolis_carolinensis CCACCTCTAGACCTTTCCATTTCCTCCCCTGAAACCATGACTTTGCCTCT

Xenopus_tropicalis --------------------GTTGTGTTTACAGTGGACAATTTAGCCTGC

Homo_sapiens ---CG-GCGCTTGCGGC---------TCCGGAGGATTCCAG-------CG

Gorilla_gorilla ---CG-GCGCTTGCGGC---------TCCGGAGGATTCCAG-------CG

Otolemur_garnettii GCTCG-CCGCTCGCAGC---------TCCGGAGGATTTCAG-------CG

Mus_musculus ---CA-GTGCTCGCGGC---------TCCGGAGGATTCCAG-------CG

Rattus_norvegicus ---CA-GTGTTCGCGGC---------TCCGGAGGATTCCAG-------CG

Jaculus_jaculus ---CC-GGGGTGGGGGTGGCTCGGCGGCCGGAGGATTCCAG-------CG

Cavia_porcellus ---CG-GCGCTCGCGGC---------TCCGGAGGATCCCAG-------CG

Ochotona_princeps CCCGGCGCGCTCGCCGC---------CCCGGAGGCTCCCAGCGCCACTCG

Sorex_araneus ---CG-CCGC-GGCCGC---------TCCGGAGGATCCCCG-------AG

Sus_scrofa ---CG-CCGCTTGCCGC---------TCCGGAGGATTCCAG-------CG

Bos_taurus ---CG-CCGCTTGCGGC---------TCCGGAGGATTCCAG-------CG

Tursiops_truncatus ---CG-CCGCTTGCCGC---------TCCGGAAGATTCCAG-------CG

Canis_familiaris ---CG-GCCTGGGCCGC---------TCCGGAGGCTTCCAG-------CG

Loxodonta_africana ---GG-GCGCTAGCGGA---------TCCGGAGGATTCCTG-------CG

Procavia_capensis ---GG-GCGCTTGCGGC---------TCCGGAGGATTCCAG-------CG

Choloepus_didactylus ---GG-GCGCTCGCGGC---------TCCGGAGGATTCCAG-------AG

Myrmecophaga_tridactyla ---GG-GCGCTTGCGGC---------TCCGGAGGATTCAAG-------AG

Macropus_eugenii ---GA-GGCTTTCCAGC----------CAAGAGGGAGGGAA-------GG

Sarcophilus_harrisii ---GA-GGCTTTCCCAG---------TCAGGAGGAAGGGAG-------GG

Anolis_carolinensis CTCCTAACCTAGGAAATGGAAGATTGCATATAATTTCCCTTCTTTATATA

Xenopus_tropicalis TATTTAAGATCATCAAT---------GTAACAGAAATATAAATAAATGTA

Homo_sapiens A-----CTCGGGAGGGGCGGGAGGGGGG--TCCCCGGTGCTCGGATCTCG

Gorilla_gorilla A-----CTCGGGAGGGGCGGGAGGGGGG--TCCCCGGTGCTCGGATCTCG

Otolemur_garnettii A-----CTCGGGAGGGGTGGGAGAGGGG--TCCCCGGTGCTCGGATCTCG

Mus_musculus A-----CTCGGGAGGGGCGGGAGGGGGG--TCCCCGGTGCTCGGATCTCG

Rattus_norvegicus A-----CTCGGGAGGGGCGGGAGGGGGG--TCCCCGGTGCTCGGATCTCG

Jaculus_jaculus A-----CTCGGGAGGGGCGGGAGGGGGG--TCCCCGGTGCTCGGATCTCG

Cavia_porcellus A-----CTCGGGAGGGGCGGGAGGGGGG--TCCCCGGTGCTCGGATCTCG

Ochotona_princeps A-----CTCGGGAGGGGCGGGAGGGGGG--TCCCCGGTGCCCGGACCTCG

Sorex_araneus A-----CTTGGGAGGGGCGGGAGGGGGG--TCCCCGGTGCTCGGATCTCG

Sus_scrofa A-----CTCGGGAGGGGCGGGAGGGGGC--TCCCCGGTGCTCGGATCTCG

Bos_taurus A-----CTCGGGAGGGGCGGGAGGGGGG--TCCCCGGTGCTCGGATCTCG

Tursiops_truncatus A-----CTTGGGAGGGGCGGGAGGGGGG--TCCCCGGTGCTCGGATCTCG

Canis_familiaris A-----CTCGGGAGGGGCGGGAGGGGGG--TCCCCGGAGCTCGG-TCTCG

Loxodonta_africana A-----TTCGGGAGGGGCGGGAGGGGGG--TCCCCGGTGCTCGGATCTCG

Procavia_capensis A-----CTCGGGAGGGGCGGGAGGGGGG--TCCCCGGTGCTCGGATCTCG

Choloepus_didactylus A-----CTCGGGAGGGGCGGGAGGGGGG--TCCCCGGTGCTCGGATCTCG

Myrmecophaga_tridactyla A-----CTCGGGAGGGGCGGGAGGGGGG--TCCCCGGTGCTCGGATCTCG

Macropus_eugenii AGGAAGCAGGGGAGGATAAGGGAGGGGG-ATCCCCTTGTGTCTGGGTCTG

Sarcophilus_harrisii AGGAAGCAGGGGAGGATAAGGGAGGGGG-ATCCCCTTGTGTCCCGGTCTG

Anolis_carolinensis TACTTTCTCAGGGTTGAAGTGGAAATTACTTTTTTAGTGACTAACCTTTG

Xenopus_tropicalis GTCAACGACTGCAATTACTAAGCCAAAC--AAAATAGTGGCGAGAACCAG

Homo_sapiens AGGGTGCTTATTG-TTCG--------------------------------

Gorilla_gorilla AGGGTGCTTATTG-TTCG--------------------------------

Otolemur_garnettii AGGGTGCTTATTG-TTCG--------------------------------

Mus_musculus AGGGTGCTTATTG-TTCG--------------------------------

Rattus_norvegicus AGGGTGCTTATTG-TTCG--------------------------------

Jaculus_jaculus AGGGTGCTTGTTG-ATCG--------------------------------

Cavia_porcellus AGAGTGCTTATTG-TTCG--------------------------------

Ochotona_princeps AGGGTGCTGGCCG-TTCG--------------------------------

Sorex_araneus AGGGTGCTTATTG-TTCG--------------------------------

Sus_scrofa AGGGTGCTTATTG-TTCG--------------------------------

Bos_taurus AGGGTGCTTATTG-TTCG--------------------------------

Tursiops_truncatus AGGGTGCTTATTGTTTCG--------------------------------

Canis_familiaris AGGGTGCTTATTG-TTCG--------------------------------

Loxodonta_africana AGGGTGCTTATTG-TTCG--------------------------------

Procavia_capensis AGGGTGCTTATTG-TTCG--------------------------------

Choloepus_didactylus AGGGTGCTTATTG-TTCG--------------------------------

Myrmecophaga_tridactyla AGGGTGCTCATTG-TTCG--------------------------------

Macropus_eugenii GAGG-TCTTATTG-TCTC--------------------------------

Sarcophilus_harrisii GAGG-TCTCATTG-TCTC--------------------------------

Anolis_carolinensis CTTGAACTCAAAA-CTTTAATATACATACACATATGTATATATATGCACA

Xenopus_tropicalis TGCAATCACAGAA-TTTGTAAAACCAGGCAAATGGTTTTTAAAAAAAAAG

Homo_sapiens ---GTCCGAG-CCTGGGTCTCCCTCTTCCC----------------CCCA

Gorilla_gorilla ---GTCCGAG-CCTGGGTCTCCCTCTTCCC----------------CCCA

Otolemur_garnettii ---GTCCGAGCCCTGGGTCTCCCTCTTCCC----------------CCCC

Mus_musculus ---GTCCGAG-CCTGGGTCTCCCTCTTCCC----------------CCCA

Rattus_norvegicus ---GTCCGAG-CCTGGGTCTCCCTCTTCCC----------------CCCA

Jaculus_jaculus ---GTCCGAG-CCTGGGTCTCCCTCTTCCC----------------CCCA

Cavia_porcellus ---GTCCGAG-CCTGGGTCTCCCTCTTCCC----------------CCCA

Ochotona_princeps ---GTCCGAG-CCTGGGTCTCCCTCTTCCG----------------CCCA

Sorex_araneus ---GTCCGAG-CCTGGGTCTCCCTCTCCCC----------------CCA-

Sus_scrofa ---GTCCGAG-CCTGGGTCTCCCTCTCCCC----------------CCCA

Bos_taurus ---GTCCGAG-CCTGGGTCTCCCTCTCCCC----------------C---

Tursiops_truncatus ---GTCCGAG-CCTGGGTCTCCCTCTCCCC----------------CCCA

Canis_familiaris ---GTCCGAG-CCCGGGTCTCCCTCTCCCC----------------CCAG

Loxodonta_africana ---GTCCGAG-CCTGGGTCTCCCTCTCCCC----------------CCA-

Procavia_capensis ---GTCCGAG-CCTGGGTCTCCCTCTCCCC----------------CCA-

Choloepus_didactylus ---GTCCGAG-CCCGGGTCTCCCTCTCCCC----------------CCT-

Myrmecophaga_tridactyla ---GTCCGAG-CCTGGGTNTCCCTCTCCCC----------------CTCA

Macropus_eugenii ---GCTGGAG-TCCGGGTCTCCCTCCCCAT----------------C---

Sarcophilus_harrisii ---TCTGGAG-TCCGGGTCTCCCTCCTCA---------------------

Anolis_carolinensis TATGTACATA-TGTGTGTGTATATATGCACATATGCATATATATATATCT

Xenopus_tropicalis TGTATCAGGA-AAAAAATGACTGATTTTAT--------------GTTCAT

Homo_sapiens ACC---CCCCCTCAGCCCCT-----CCGG-CTGCAGAGTGAAGGC----T

Gorilla_gorilla ACC---CCCACTCAGCCCCT-----CCGG-CTGCAGAGTGAAGGC----T

Otolemur_garnettii -AC---CCCCTTCAGCCCCT-----CCGG-TTGCTGAGTGAAGCC----T

Mus_musculus -TC---CCCCCTCAGCCCCT-----CCGG-CCGCAGAGTGAAGGC----G

Rattus_norvegicus -TC---CCCCCTCAGCCCCT-----CCGG-CCGCAGAGTGAAGGC----G

Jaculus_jaculus -GC---CCCCTTCGGCCTCT-----CCGG-CTGCGGAGTGCAGGC----G

Cavia_porcellus -CA---CCCCCCTCAGCCCT-----CCGG-CTGCGGAGTGAAGGC----C

Ochotona_princeps CCCCCTCCCCCTCCGCCCCTCCGGGCCGG-CCGCCGAGTGAAGGC----T

Sorex_araneus ------CCCCCTCAGCCCCT-----CGGGCCCGCAGAGTGCAGGCCGCGC

Sus_scrofa ------CCCCCTCAGCCCCT-----CCGG-CTGCTGAGTGAAGGC----T

Bos_taurus ------CACCCCCAGCCCCT-----CCGG-CTGCTGAGTGAAGGC----T

Tursiops_truncatus ------CCCCCTCAGCCCCT-----CTGG-CTGCTAAGTGAAGGC----T

Canis_familiaris --------CCCTCAGCCCCT-----ACGG-CTGCAGAGTGAAGGC----T

Loxodonta_africana ------CCCCTTCAGCCCCT-----CCGG-CTGCTGAGTGAAGGC----T

Procavia_capensis ------CCCCTTCAGCCCCT-----CCGG-CTGCTGAGTGAAGGC----T

Choloepus_didactylus -CA---CCCCCTCAGCCCCT-----CCGG-CTGCTGAGTGAAGGC----T

Myrmecophaga_tridactyla ------CCCCCTCAGCCCCT-----TCGG-CTGCTGAGTGAAGGC----T

Macropus_eugenii ------CCCCTCGGCCCCCT-----CTGG----GTAAGTGAGTGT----C

Sarcophilus_harrisii -CC---CCCCCCTCACCTTT-----CTGG----GTAAGTGAGTGT----C

Anolis_carolinensis GCGTATATCTATCTATCTGTATATATATGTGTGTGTGATACATACATGAT

Xenopus_tropicalis ACT---TCTACAAAGTGTGACTGCACTTTTCACTAAGGCAACTATGAAAG

Homo_sapiens ------GCGGTGGAAAGTTTCCTGC------------------------C

Gorilla_gorilla ------GCGGTGGAAAGTTTCCTGC------------------------C

Otolemur_garnettii ------GCGGTGGAAAGTTTCCTGC------------------------C

Mus_musculus ------GCGGTGGAAAGTTTCCTGC------------------------C

Rattus_norvegicus ------GCGGTGGAAAGTTTCCTGC------------------------C

Jaculus_jaculus ------GTGGTGGAAAGTTTCCTGCGGGGCCGGCGCGCGGGGCAGGCGGG

Cavia_porcellus ------GCGGTGGAAAGTTTCCTGC------------------------C

Ochotona_princeps ------GCGATGCAAAGTTTCCTTC------------------------C

Sorex_araneus ------GCGGCGGAAAGTTTGCTGC------------------------C

Sus_scrofa ------GCGGCGGAAAGTTTCCTGC------------------------C

Bos_taurus ------GCGGCGGAAAGTTTCCTGC------------------------C

Tursiops_truncatus ------ACGGCTGAAAGTTTCCTGC------------------------C

Canis_familiaris ------GCGGTGCAAAGTTCCT--G------------------------C

Loxodonta_africana ------GCGGCGCAAAGTTTCCTGC------------------------C

Procavia_capensis GCAGCGGCGGCGCAAAGTTTACTGC------------------------C

Choloepus_didactylus ------ACGGCGGAAAGTTTCCTGC------------------------C

Myrmecophaga_tridactyla ------ACGGCAGAAAGTTTCCTGC------------------------C

Macropus_eugenii ------GGAGCGGAAAGTTTCCCTA------------------------G

Sarcophilus_harrisii ------GGAGCGGAAAGTTTCC---------------------------C

Anolis_carolinensis ACACGCATATATAAAGTATGGGTAA------------------------G

Xenopus_tropicalis CTCTGTGTGTATCGAATTTAATTGT------------------------G

Homo_sapiens TGG-----GCGGAGGCGCCTCTCCCG------------------------

Gorilla_gorilla TGG-----GCGGAGACGCCTCTCCCG------------------------

Otolemur_garnettii AGG-----GCGGAGGCGCCTCTCACC------------------------

Mus_musculus CAG-----GCGGAGGCGCTTCTCCCG------------------------

Rattus_norvegicus CAG-----GCAGAGGCTCTTCTCCCG------------------------

Jaculus_jaculus CGG-----GCGGAGGCGCCGCTCTCC------------------------

Cavia_porcellus CGG-----GCGGAGGCGCCTTTCCCG------------------------

Ochotona_princeps CGGGGCGGGCGGGCGTGCGTGTCGGC-------------------GGGCG

Sorex_araneus TG------GCGGAGGCGCCTCTTCGG------------------------

Sus_scrofa TGG-----GCGGAGGCGCCTCTCCCC------------------------

Bos_taurus TGG-----GCGGAGGCTCCTCTCCCG------------------------

Tursiops_truncatus CGG-----GCGGAGGCGCCTCTCCCG------------------------

Canis_familiaris CGG-----GCCGAGGCGCCTCTCCCGGGCGGGCCGGGCCGGGCTGCACCG

Loxodonta_africana AGG-----GCGGAGGCGTATCTCCCG------------------CGGCTA

Procavia_capensis AGG-----GC-GAGGCGTCTCTCTCG------------------------

Choloepus_didactylus AGG-----GCGGAGGCGCCTCTCC-----------------------CGT

Myrmecophaga_tridactyla AGG-----GCGGAGGTGCCTCTCCGC------------------GGGCTG

Macropus_eugenii CACAGAGCCAAGTGTCGCCTCTCCCT------------------------

Sarcophilus_harrisii TAG-----CAAGTGTCGCCTCTCCCT------------------------

Anolis_carolinensis AAG-----AAAAAAGGTTCTGTTACT-------TTCCTATATTTTGTTCT

Xenopus_tropicalis GGG-----GGGGGGGGGGTTATGTAT------------------------

Homo_sapiens ----GGGCT---GGGCTGGGCT------------------------GGCC

Gorilla_gorilla ----GGGCT---GGGCTGGGCT------------------------GGCC

Otolemur_garnettii ----GGGCT---GGGCTGGGCTGG------------------ACT-GGTC

Mus_musculus ----GGACC---GGGCC-----------------------------TGCC

Rattus_norvegicus ----GGACT---GGGCC-----------------------------TGTC

Jaculus_jaculus ----GGGGG---AAGCTGGGCCCG------------------GTC-CGCC

Cavia_porcellus ----GGGCT---CGGCTGGGCCGG------------------GCCTGTCC

Ochotona_princeps GTGGAGGCGCCTTGGCCCCGGGGC------------------TCCGGGCC

Sorex_araneus ----GGGCC---GGGCCGGGC-----------------------------

Sus_scrofa ----GGGCC---AGGCTGGGCTGG------------------GCCGGGCC

Bos_taurus ----GGGCC---AGGCTGGGCTGG------------------GCCGGGCC

Tursiops_truncatus ----GGGCC---AGGCTGGGCTGG------------------GCCGGGCC

Canis_familiaris GGCTGCGCC---GGGCTGCGATGG------------------GCCGGGCC

Loxodonta_africana TACTGGACT---AGGCTGGGCCGG------------------GCCGGGCC

Procavia_capensis ------------GGGCCGGGCTGG------------------GCCGGGCC

Choloepus_didactylus GGCTGGGCT---GGGCTGGGCTGA------------------GTCGCGCC

Myrmecophaga_tridactyla GGCTGGGCT---GGGCTGGACTGA------------------GTCGGACC

Macropus_eugenii ----AGGGCCAGGGGCTGCTTTCG------------------TTCCCACC

Sarcophilus_harrisii ----AGGATCAGGGGCCGCTTTCA------------------TCCCCTCC

Anolis_carolinensis AAAAAGGTTCTGTTACTTTCCTAGTTGTTGTTTACCTATGTTGTTCGTTC

Xenopus_tropicalis ---------------------------------------------TCGTT

Homo_sapiens ----CGCCTGCGGCCCGCGTGGCCTG-------TCTTGCGGCTCTCGCCT

Gorilla_gorilla ----CGCCTGCGGCCCGCGTGGCCTG-------TCTTGCGGCTCTCGCCT

Otolemur_garnettii ----CGCCTGCGGCCCGCCCGGCCTG-------TCTCGCCTCTTTTGCCT

Mus_musculus ----CGCCTGCGGCCCTTCTGGCCTG-------TCCCACCGCGCTCACCT

Rattus_norvegicus ----CGCCTGCGGCCCTTCTGGCCTG-------TCCCACCGCGCTCACCT

Jaculus_jaculus ----CGCCTGCGGCCCACCGGCCCCGGGGATGCCGCGCGCGCGCCTGTTT

Cavia_porcellus ------GCTGCGGCCCGCCGGGCCTG-------GCTCGCG----GCGCTG

Ochotona_princeps ----CGCGCGGGGCCTGCCCGGCCGT-----------GCTCCCCTCTCTC

Sorex_araneus -------------CCGCCGGGGCCTG-------CGGCGCCCCGTGCGCCC

Sus_scrofa ----CGCCTGCGGCCCGCCGGGCCTG-------TCCTGCCCTGCTCGCCT

Bos_taurus ----CGCCTGCGGCCCGCCGGGCCTG-------TCCGGCCCCGCTCGCGT

Tursiops_truncatus ----CGCCTGCGGCCCGCCGGGCCTG-------TCCTGCCCCGCTCGCGT

Canis_familiaris ----CGCCTGCGGCCCGCGGGGCCTG-------TGCCGGCCGCTCCGGG-

Loxodonta_africana ----CGCCTGCGGCCCGCCGGGCCTG-------GCTTG-CCGGCTCGCCT

Procavia_capensis ----CGCCTGCGGCCCGCCAGGCCTG-------CCCTGCCCGGCTCGCCT

Choloepus_didactylus ----CGCCTGCGGCCAGCCGGGCCTG-------TCTTACCCCGCTCGCTT

Myrmecophaga_tridactyla ----TCCCTGCGGCCAGCCGGGCCTG-------TCATGCCCCGCTCGCTC

Macropus_eugenii CTTACCTCCTTTGCCCGCCTGCCCTG-------TTCTCTAGTCAGTTGGG

Sarcophilus_harrisii TTTACCCCCTTTGCCCGCCTGCCCAG-------TTTTTTAGTCAGCTGGG

Anolis_carolinensis AAAATAGTTGTTGTTTACCTATGTTGTTGCTGTTGTCATTTATTATTATT

Xenopus_tropicalis AAAAGAATTGTGAAAAATAAAGCCCGGGGAGTCTATAAGACGTTGTTCTG

Homo_sapiens CCTCTCCCTCCGGCCG------CGGGTGGG-GGCCTG----GGCTGGGGT

Gorilla_gorilla CCTCTCCCTCCGGCCG------CGGGTGGG-GGCCTG----GGCTGGGGT

Otolemur_garnettii CCTCTCCCTCCCGCCG------CGGGCGGG-GGCCTGG---GGCTGGGGG

Mus_musculus CTACTCCCTCCGACCA------CGGGTGGG--GCCTGG---GACTAGGGT

Rattus_norvegicus CCACTCCCTCCGACCA------CGGGTGGG--GCCTGG---GACTGGGGT

Jaculus_jaculus CCTCGCTCTCCGGCCG------CGGGTGGG-GG--------GGCCCGGGT

Cavia_porcellus CCTCTCTCTCCGGCTG------CGGGTGGG-GGCTGGG---GACAGGGAT

Ochotona_princeps CCTCTCCCATCGTTCGCGTCGGGGGAAGGGAGGACCCCCGGGGCTGGGCT

Sorex_araneus CCTCTCCCTGCGGCCG------CGAGTGGG-GGCCCGGGCTGGCTGGGAT

Sus_scrofa CGTCTCCCTCTGGCCG------CGGGTGGG-GGCCTG----GGCTGGGAC

Bos_taurus CTTCTCCCTCTGGCCG------CGGGTGGG-GGCCTGG---GGCTGGGAT

Tursiops_truncatus CTTCTCCCTCCGGCCG------CTGGTGGG-GGCCTGG---GGCTGGGAT

Canis_familiaris ---CTCCCTGCGGCCG-------GGGTGGG-GGCCTG----GGCCGGGGC

Loxodonta_africana CTTCTCCCTCCGGCCG------CGGGTGGGAGGCCTGG---AGCTGGGGT

Procavia_capensis CTCTTCCTCCAGCCAG------CGGTTGGGAGGCCTGG---TGCTGGGGT

Choloepus_didactylus CTC--TCCCCAGGCCG------CGGGTGGGGGGCCTGG---GATCTGGGT

Myrmecophaga_tridactyla CTC--TCCCCAGGCCG------CGGGTGGGGGACCCGG---GATGAGGGT

Macropus_eugenii GCTAGTTCTGAGACCCATCTT-AGGGTGGG--GGTGGA---GGTGGGGGT

Sarcophilus_harrisii GCTAGTTCTGAGACCGATCTTAAGGGTGGG--GAGTGG---GGGTGGGGT

Anolis_carolinensis ATTATTATTATTATTATTATTATTATTATTATTATTAT---TATTAACTG

Xenopus_tropicalis ATTATTTTCTTGCTTGC---------------------------------

Homo_sapiens GGGGACG---GGGGAACGCT----GCAAG---CTATTCACCCCTTCCTGG

Gorilla_gorilla GGCGACA---GGGGACCGCT----GCAAG---TTATTCACCCCTTCCTGG

Otolemur_garnettii GGAGGTGGGCACTGGGCACT----GCACG---CTATTCACCCCTTCCTGG

Mus_musculus GGG-ACG---TTGGAGTGCT----GCACG---CTATTCATCCCTCCCTGG

Rattus_norvegicus GGG-ACG---TTGGGGTGCT----GCACG---CTATTCACCCCTCCCTGG

Jaculus_jaculus GGGAAC----CTGGGGTGCT----GCACG---CTATTCACCCCTGCCTGG

Cavia_porcellus GGG-GCG---CTGGGGCACT----GCACA---CTATTCACCCCTTCCTGG

Ochotona_princeps TGCA------GGGGTGCGTG----GCACG---CTGTTCATTCCTGCCTGG

Sorex_araneus GGGCACG---CCGGGGCGCT----ACCCGCTACTATTCACCCCTGCCGGG

Sus_scrofa CGGGACG---CTGGGGCGCT----GCACG---CTATTCACCCCTTCCTGG

Bos_taurus GGGGACG---CTGGGGTGCT----GCACG---CTATTCACCCCTTCCTGG

Tursiops_truncatus GGGGAAG---CTGGGGCGCT----GCACG---CTATTCACCCCTTCCTGG

Canis_familiaris GGG-CCG---CTGGGGCGCT----GCTCC---CTATTCACCCCTCCCTGG

Loxodonta_africana GGGGGCG---CTGGGACGGT----GCACG---CTATTCACCCCTTCCTGG

Procavia_capensis GGGTGCG---CTGGGACGGT----GCACG---CTATTTACTCCTTCCTGG

Choloepus_didactylus GGGGGCG---CTGGGGCGCT----GCACG---CTATTCACCCCTTCCTGG

Myrmecophaga_tridactyla GGGG----------------------------------------------

Macropus_eugenii AGGGAGA---CGGTGGGACTTTTAAAAAA---CTATTCACCCGTTCCTGC

Sarcophilus_harrisii AGGGAGA---CGGTGGGACTTTTTAAAAA---CTATTCACCCGTTCCTGC

Anolis_carolinensis CTGCAGCTGCTGCAGCTGCTGCTGCTACTATTAGGTTTTTTTATCCCATG

Xenopus_tropicalis --------------------------------CCCCTCCCCTTGTCCCCC

Homo_sapiens CTT----GGGGTGGG----------G-TTTATGTTCCAGAGACGGACGGC

Gorilla_gorilla CTT----GGGGTGGG----------G-TTTATGTTCCAGAGACGGACGGC

Otolemur_garnettii CTT----GGGCGGGG----------G-GTTATGTTCCAGAAACGGATGGC

Mus_musculus TTT----GG---GGG----------AATTTATGTTCCAGAGACGGATGGC

Rattus_norvegicus TTT----TG---GGG----------GATTTATGTTCCAGAGACGGATGGC

Jaculus_jaculus CTT----GG---GGG----------GATTTATGTTCCAGAGACGGATGGC

Cavia_porcellus CGT----GG---GGG----------GATTTATGTTCCAGAGACGGATGGC

Ochotona_princeps GGTTTTTTGGGGGGGTTGGGGGTACGGTTTCTGTTACAGAGACGGACGGC

Sorex_araneus CTT----GG---GGG----------G-TTTATGTTCCAGAGACGGACGGC

Sus_scrofa CTT----GG---GGG----------G-TTTATGTTCCAGAGACGGACGGC

Bos_taurus CTT----GG---GGG----------G-TTTATATTCCAGAGACGGACGGC

Tursiops_truncatus CTT----GG---GGG----------G-TTTATATTCCAGAGACTGACGGC

Canis_familiaris CTT----GGG--GGG----------G-CTTCTATTCCAGAGACGGACGGC

Loxodonta_africana CTT----GG---GGG----------G-CTTATGTTCCAGAGACGGACGGC

Procavia_capensis CTT----GG---GGG----------G-CTTATGTTCCAGAGACCGACGGC

Choloepus_didactylus CTTGG--GGTGGGGG----------G-TTTATGTTCCAGAGACGGATGGC

Myrmecophaga_tridactyla --------------------------------------------------

Macropus_eugenii ACTTGCTGGGGTGGG----------G-TTTATATTCCAGAGACAGACGGC

Sarcophilus_harrisii ACTTGCTGGGGTGGG----------G-TTTATATTCCAGAGACAGACGGC

Anolis_carolinensis TCT------------------------TTTATATTCCAGAAGCAGATGGC

Xenopus_tropicalis CCC------------------------CCTACTCCCTAGAAACGGACGGG

Homo_sapiens AAGCGGTCCCGAACCAGTTACACGCGCTACCAGACTCTGGAACTCGAGAA

Gorilla_gorilla AAGCGGTCCCGAACCAGTTACACGCGCTACCAGACTCTGGAACTCGAGAA

Otolemur_garnettii AAGCGGTCCCGAACCAGTTACACGCGCTACCAGACTCTGGAACTCGAGAA

Mus_musculus AAGCGGTCCCGAACCAGTTACACGCGCTACCAGACCCTGGAACTCGAGAA

Rattus_norvegicus AAGCGGTCCCGAACCAGTTACACGCGCTACCAGACCCTGGAACTCGAGAA

Jaculus_jaculus AAGCGGTCCCGAACCAGTTACACGCGCTACCAGACCCTGGAACTCGAGAA

Cavia_porcellus AAGCGGTCCCGAACCAGTTACACGCGCTACCAGACTCTGGAACTCGAGAA

Ochotona_princeps AAGCGGTCGCGAACCAGTTACACGCGCTACCAGACCCTGGAACTCGAGAA

Sorex_araneus AAGCGGTCCCGAACCAGTTACACGCGCTACCAGACTCTGGAACTGGAAAA

Sus_scrofa AAGCGGTCCCGAACCAGTTACACGCGCTACCAGACTCTGGAACTGGAAAA

Bos_taurus AAGCGGTCCCGAACCAGTTACACGCGCTACCAGACTCTGGAACTAGAAAA

Tursiops_truncatus AAGCGGTCCCGAACCAGTTACACGCGCTACCAGACTCTGGAACTGGAAAA

Canis_familiaris AAGCGGTCCCGAACCAGTTACACGCGCTACCAGACTCTGGAACTGGAAAA

Loxodonta_africana AAGCGGTCCCGAACCAGTTACACGCGCTACCAGACTCTGGAACTCGAGAA

Procavia_capensis AAGCGGTCCCGAACCAGTTACACGCGCTACCAGACTCTGGAACTGGAGAA

Choloepus_didactylus AAGCGATCCCGAACCAGTTACACGCGCTACCAGACTCTGGAATCACTAGT

Myrmecophaga_tridactyla --------------------------------------------------

Macropus_eugenii AAGCGATCCAGAACCAGTTACACGCGCTACCAGACTTTGGAATTAGAGAA

Sarcophilus_harrisii AAGCGATCCAGAACCAGTTACACGCGCTACCAGACTTTGGAATTAGAGAA

Anolis_carolinensis AAACGCTCCAGAACCAGCTACACTCGTTACCAAACTCTGGAACTAGAGAA

Xenopus_tropicalis AAGCGTTCCCGGACAAGTTACACTCGATACCAGACCCTGGAGCTGGAGAA

Homo_sapiens AGAATTCCACTTTAACCGCTACCTCACTCGCCGCAGGCGCATAGAGATCG

Gorilla_gorilla AGAATTCCACTTTAACCGCTACCTCACTCGCCGCAGGCGCATAGAGATCG

Otolemur_garnettii AGAATTTCACTTTAACCGCTACCTCACTCGCCGCAGGCGCATAGAGATCG

Mus_musculus AGAATTCCATTTTAACCGCTACCTCACTCGCCGCAGGCGCATAGAGATTG

Rattus_norvegicus AGAATTCCATTTTAACCGCTACCTCACTCGCCGCAGGCGCATAGAGATCG

Jaculus_jaculus AGAATTTCACTTTAACCGCTACCTCACTCGCCGGAGGCGCATAGAGATCG

Cavia_porcellus AGAATTCCACTTTAACCGCTACCTCACTCGCCGTAGGCGCATAGAGATCG

Ochotona_princeps AGAATTCCACTTTAACCGCTACCTCACTCGCCGCAGGCGCATCGAGATCG

Sorex_araneus AGAATTCCACTTTAACCGCTACCTCACTCGCCGCAGGCGCATAGAGATCG

Sus_scrofa AGAATTCCACTTTAACCGCTACCTCACTCGCCGCAGGCGCATAGAAATCG

Bos_taurus AGAATTCCACTTTAACCGCTACCTCACTCGCCGCAGGCGCATAGAGATCG

Tursiops_truncatus AGAATTCCACTTTAACCGCTACCTCACTCGCCGCAGGCGCATAGAGATCG

Canis_familiaris AGAATTCCACTTTAACCGCTACCTCACTCGCCGCAGGCGCATCGAGATCG

Loxodonta_africana AGAATTCCACTTTAACCGCTACCTAACTCGCCGCAGGCGCATAGAGATCG

Procavia_capensis AGAATTCCACTTTAACCGCTACCTCACTCGCCGCAGGCGCATAGAGATTG

Choloepus_didactylus GAATT---------------------------------------------

Myrmecophaga_tridactyla --------------------------------------------------

Macropus_eugenii AGAATTTCACTTTAACCGCTACCTCACCCGCCGAAGGCGAATTGAGATCG

Sarcophilus_harrisii AGAATTTCACTTTAACCGCTACCTCACCCGCCGAAGGCGAATTGAGATCG

Anolis_carolinensis AGAGTTCCATTTTAACAGATATCTCACCCGCCGGCGGCGCATAGAGATCG

Xenopus_tropicalis GGAATTCCACTTTAATAGATACCTGACTCGCCGAAGGCGCATAGAGATCG

Homo_sapiens CCAACAACTTGTGTCTCAATGAGAGACAGATCAAGATCT---GGTTCCAG

Gorilla_gorilla CCAACAACTTGTGTCTCAATGAGAGACAGATCAAGATCT---GGTTCCAG

Otolemur_garnettii CCAACAACCTGTGTCTCAATGAGAGACAGATCAAGATCCTTGGTTCCCAG

Mus_musculus CCAACAACTTGTGTCTCAACGAGAGACAGATCAAGATCT---GGTTCCAG

Rattus_norvegicus CCAACAACTTGTGTCTCAACGAGAGACAGATCAAGATCT---GGTTCCAG

Jaculus_jaculus CCAACAACTTGTGTCTCAACGAGAGACAGATCAAGATCT---GGTTCCAG

Cavia_porcellus CCAACAACTTATGTCTCAACGAGAGACAGATCAAGATTT---GGTTCCAG

Ochotona_princeps CCAACAACTTGTGTCTCAACGAGAGACAAATCAAGATCT---GGTTCCAG

Sorex_araneus CCAACAACTTGTGTCTCAACGAGAGACAGATCAAGATCT---GGTTCCAG

Sus_scrofa CCAACAACTTGTGTCTCAACGAGAGACAGATCAAGATCT---GGTTCCAG

Bos_taurus CCAACAACTTGTGTCTCAACGAGAGACAGATCAAGATCT---GGTTCCAG

Tursiops_truncatus CCAACAACTTGTGTCTCAACGAGAGACAGATCAAGATCT---GGTTCCAG

Canis_familiaris CCAACAACTTGTGTCTCAACGAGAGACAGATCAAGATCT---GGTTCCAG

Loxodonta_africana CCAACAACTTGTGTCTCAACGAGAGACAGATCAAGATCT---GGTTCCAG

Procavia_capensis CCAACAACTTGTGTCTCAACGAGAGACAGATCAAGATCT---GGTTCCAG

Choloepus_didactylus --------------------------------------------------

Myrmecophaga_tridactyla --------------------------------------------------

Macropus_eugenii CTAACAACTTGTGTCTCAACGAGAGACAGATCAAGATCT---GGTTCCAG

Sarcophilus_harrisii CCAACAACTTGTGTCTCAACGAGAGACAGATCAAGATCT---GGTTCCAG

Anolis_carolinensis CCAACAACCTCTGTCTGAATGAGAGGCAGATCAAAATAT---GGTTTCAA

Xenopus_tropicalis CCAACAACCTCTGTCTAAACGAAAGGCAGATCAAAATTT---GGTTCCAG

Homo_sapiens AACCGCAGGA-TGAAGTGGAAGAAAGA--TTCCAAAATGAAAA---GCAA

Gorilla_gorilla AACCGCAGGA-TGAAGTGGAAGAAAGA--TTCCAAAATGAAAA---GCAA

Otolemur_garnettii AACCGCATGATTGAAGTGGAAGAAAGGATTCCCAAAATGAAAAAGCAAAA

Mus_musculus AACCGCAGGA-TGAAGTGGAAGAAAGA--TTCCAAAATGAAGA---GCAA

Rattus_norvegicus AACCGCAGGA-TGAAGTGGAAGAAAGA--TTCCAAAATGAAGA---GCAA

Jaculus_jaculus AACCGCAGGA-TGAAGTGGAAGAAAGA--TTCCAAAATGAAAA---GCAA

Cavia_porcellus AACCGCAGGA-TGAAGTGGAAGAAAGA--TTCCAAAATGAAAA---GCAA

Ochotona_princeps AACCGAAGGA-TGAAGTGGAAGAAAGA--TTCCAAGATGAAAA---GCAA

Sorex_araneus AACCGCAGGA-TGAAGTGGAAGAAAGA--TTCCAAAATGAAAA---GCAA

Sus_scrofa AACCGCAGGA-TGAAGTGGAAGAAAGA--TTCCAAAATGAAAA---GCAA

Bos_taurus AACCGCAGGA-TGAAGTGGAAGAAAGA--TTCCAAAATGAAAA---GCAA

Tursiops_truncatus AACCGCAGGA-TGAAGTGGAAGAAAGA--TTCCAAAATGAAAA---GCAA

Canis_familiaris AACCGCAGGA-TGAAGTGGAAGAAAGA--TTCCAAAATGAAAA---GCAA

Loxodonta_africana AACCGCAGGA-TGAAGTGGAAGAAAGA--TTCCAAAATGAAAA---GCAA

Procavia_capensis AACCGCAGGA-TGAAGTGGAAGAAAGA--TTCCAAAATGAAAA---GCAA

Choloepus_didactylus --------------------------------------------------

Myrmecophaga_tridactyla --------------------------------------------------

Macropus_eugenii AACCGAAGGA-TGAAATGGAAGAAGGA--TTCCAAATTAAAAA---GCAA

Sarcophilus_harrisii AACCGAAGGA-TGAAATGGAAGAAGGA--TTCCAAATTAAAAA---GCAA

Anolis_carolinensis AACCGGAGGA-TGAAATGGAAGAAAGA--TTCCAAATTGAAAA---GCAA

Xenopus_tropicalis AACCGACGGA-TGAAATGGAAGAAAGA--TACAAAAGTCAAGA---GCAA

Homo_sapiens AGAGGCTCTTTAGAGG-CAGCG----------------GGGGAGG-----

Gorilla_gorilla AGAGGCTCTTTAGAGG-CAGCG----------------GGGGAGG-----

Otolemur_garnettii GAGGGCTCTTTAGAGGTCTGCG----------------GGGAGGC-----

Mus_musculus AGAAGCTCTTTAGAGGAAGCAG----------------GGAGC-------

Rattus_norvegicus AGAGGCTCTTTAGAGCAAGCAG----------------GGAGCCC-----

Jaculus_jaculus AGAGGCTCTTTAAAGG-AAGC-----------------GGGGAGG-----

Cavia_porcellus AGAGGCTCTTTAGAGG-CAGC-----------------GGGAAAG-----

Ochotona_princeps AGAGGCGCTTTAGAGG-CAGCG----------------GGACGAT-----

Sorex_araneus AGAGGCTCTTTAGAGG-CGGC-----------------CGGGAGG-----

Sus_scrofa AGAGGCTCTCTAGAGG-CCGC-----------------GGGGAGG-----

Bos_taurus AGAGGCTCTCTAGAGG-CATC-----------------GGGGAGG-----

Tursiops_truncatus AGAGGCTCTCTAGAGG-CAGC-----------------GGAGAGG-----

Canis_familiaris AGAGGCTCTCTAGAGG-CG-------------------GGGGGGC-----

Loxodonta_africana AGAGGCTCTTTAGAAG-CTGT-----------------GCGGAGG-----

Procavia_capensis AGAGGCTCTTTAGAAG-CCCT-----------------GCAGAGG-----

Choloepus_didactylus --------------------------------------------------

Myrmecophaga_tridactyla --------------------------------------------------

Macropus_eugenii AGAGGCTCTTTAA-------------------------------------

Sarcophilus_harrisii AGAGGCTCTTTAAAGGGAAGAG----------------GGAGATGTGGGA

Anolis_carolinensis AGAGTCCCTCTAGGTAGAACCGCCTGAATTCAGGCTGAGAAGAGG-----

Xenopus_tropicalis AGACTCCATGTAA-----AGGA----------------GCAGAGA-----

Homo_sapiens ----------CCCGCAGAGCGCGCCCC-TAGC-CGGTTCCTGTCCCT---

Gorilla_gorilla ----------CCCGCAGAGCGCGCCCCTTAGC-CTGTTCCTGTCCCT---

Otolemur_garnettii ----------CCCGCAGACAACACTTCCGAGCGGAGCTTTGGTCCAA---

Mus_musculus -----------CCTCAGAGAGCACCCC--GGT-CTGCTGTGGTCCCT---

Rattus_norvegicus ----------TCCTCAGAGAGCACCCT--GGT-CCGCTGTGGTCCCT---

Jaculus_jaculus ----------CCCGCAGAGAGCACCCC--GGT-GGGCTTCCGTCCTT---

Cavia_porcellus ----------CCCGCGGAGAGCACCCC-AA---CCGGCTTGGTCCCT---

Ochotona_princeps ----------GCCCCGCAAGCCGGCGGTGCTCCCCGCCTCTCTCCTG---

Sorex_araneus ----------CCCGCAGCGCGCACCCC-GAGC-CCGCTTCATCCCCG---

Sus_scrofa ----------CCCGCCGAGAGCACCCC-GAGC-CCACTTCGGTCCCC---

Bos_taurus ----------CCCGCCGAGAGCACCCC-GAGC-CCGCTTCGGTCCCG---

Tursiops_truncatus ----------CCCGCCGGGAGCACCCC-GAGC-CCGCTTCGGTCCCT---

Canis_familiaris ----------CCCGCACACAGCGCCCC-GAGC-CCGCTTTCCTCCCC---

Loxodonta_africana ----------CCCGCAGAGCGCGC---------CCGCTCCAGCTCCC---

Procavia_capensis ----------CCCGCA-AGCGCGC---------CGGCTCCAGTTCCC---

Choloepus_didactylus --------------------------------------------------

Myrmecophaga_tridactyla --------------------------------------------------

Macropus_eugenii --------------------------------------------------

Sarcophilus_harrisii TATGACAATCCCTTCAAATAGTTTCTC-TAGT-TGGCTTGAATCTACCTT

Anolis_carolinensis ----------GGAGCGGGAGTGGGTGTGGGGGAGTCCTTTTACTTTG---

Xenopus_tropicalis ----------CCGGAGGAGGCAGATATTCAACCTTAGTTTTGTT------

Homo_sapiens ----GCGCCTTTCCTTTTCGCCTTTC--CTCTCT--ATATTTCGGGTCG-

Gorilla_gorilla ----GCGCCTTTCCTTTTCGCCTTTC--CTCTTT--ATATTTCGGGTCG-

Otolemur_garnettii ----GCTGCTTTCCTTTTCGGGTTTCCTCTCTCTC-CTGTTTTGGGGCG-

Mus_musculus ----GCGCCCTTCCTTTTCGGTTTTCCTCCTTCT--AGATTTT-------

Rattus_norvegicus ----GCGCCCTTCCTTTTCGGTTTTCCTCCTTCT--AGATTTT-------

Jaculus_jaculus ----GCGCCTTCCCTTCTCGGTTTTCCTCTTTCT--AGATTTTTT-----

Cavia_porcellus ----GCAACTTTCCTTTTCCGGTTT-TTCTCTGT--GTGTTTTGGGGTG-

Ochotona_princeps -------ACTTCCCTTCTCCGTTTTCTTCTCTTTCTGTATTTTGGGGCC-

Sorex_araneus ----CGCCTTCCCCCTTTCCTTCTTCCTCTCTCT--CTATTTTGGGTCT-

Sus_scrofa ----GCGCTTTTCCTTTTCCGTTTTCCTCTTTCG--ATATTTTGGGGCGA

Bos_taurus ----CGCCTTTCCCTGTCGGTTTTTCCTCTCTAG--ATATCTTGGGGGCA

Tursiops_truncatus ----GCGCCTTTTCTTTTCGGTTTTCCTTTCTCG--GTAT----------

Canis_familiaris ----GCGCCTTTCCTCCTCGGGTTTCCTCTGTCT--GTGTCT-GGGGCA-

Loxodonta_africana ----GCTCCTTTCCTTTTCTGTTTTCCTCTCTCT--AGATTTCGGGGCT-

Procavia_capensis ----GAGCCTCTCCTTTTCTGTTTTCCTCTCTCT--GTATTTTGGGGCA-

Choloepus_didactylus --------------------------------------------------

Myrmecophaga_tridactyla --------------------------------------------------

Macropus_eugenii --------------------------------------------------

Sarcophilus_harrisii GGGGAAACTTTTCATTTTCTTTTTTCCTGTTTTT--TTGTTTTGTTTTGT

Anolis_carolinensis ----AAGCAGAGTGTTTGGGGGTAGCCTACCTTGAGAAAACTTTGTCTT-

Xenopus_tropicalis ----ACATCTACTTTATGAAACTTTCTGCTTTTTGTGTG-----------

Homo_sapiens --------------------------------------------------

Gorilla_gorilla --------------------------------------------------

Otolemur_garnettii --------------------------------------------------

Mus_musculus --------------------------------------------------

Rattus_norvegicus --------------------------------------------------

Jaculus_jaculus --------------------------------------------------

Cavia_porcellus --------------------------------------------------

Ochotona_princeps --------------------------------------------------

Sorex_araneus --------------------------------------------------

Sus_scrofa --------------------------------------------------

Bos_taurus --------------------------------------------------

Tursiops_truncatus --------------------------------------------------

Canis_familiaris --------------------------------------------------

Loxodonta_africana --------------------------------------------------

Procavia_capensis --------------------------------------------------

Choloepus_didactylus --------------------------------------------------

Myrmecophaga_tridactyla --------------------------------------------------

Macropus_eugenii --------------------------------------------------

Sarcophilus_harrisii TTTATTTTGTTTTGTGTGTGTGTGCGTGTGTGTGAAATTTTTTTAGGAGT

Anolis_carolinensis --------------------------------------------------

Xenopus_tropicalis --------------------------------------------------

Homo_sapiens ---------------------GGGGCAGGTGCT-GGAGCACTGGGCTCC-

Gorilla_gorilla ---------------------GGGGCAGGTGCT-GGAGCACTGGGCTCC-

Otolemur_garnettii ----------------AG---AGAATATGTGCT-GGAGCCCC-TCGCTC-

Mus_musculus ---------------------GAGGCGGAGGCT-AGAGCACT-GGCTC--

Rattus_norvegicus ---------------------GAGGCAAAGGCT-AGAGCACT-GGCTC--

Jaculus_jaculus ---------------------GGGGCTGAGGCT-----------------

Cavia_porcellus ----------------G----GGGGCAGGAGCT-GGAGCTTT-GGCTTC-

Ochotona_princeps ---------------------AAGGGAGGGGTG-GGGGCTC---------

Sorex_araneus ----------------ATTTGGGGGCAGGGGCG-GGGGACCC-CCCTCCA

Sus_scrofa ---------------------AGGGCGGCGGCT-GGAGCACC-GGCTCC-

Bos_taurus ---------------------AGAACGGGGGCT-GGAGCGCC-GGTTCC-

Tursiops_truncatus ---------------------AGGGCGGGGGCTGGGAGCATC-GGCTCC-

Canis_familiaris ---------------------CGGCCGGGCGGC-CGGGCACC-GGC----

Loxodonta_africana ---------------------GGGGCTAGGGCT-GGAGCACC-GGCTCC-

Procavia_capensis ---------------------GGGGCTAAGGCT-GACATATC-GGCTCC-

Choloepus_didactylus --------------------------------------------------

Myrmecophaga_tridactyla --------------------------------------------------

Macropus_eugenii --------------------------------------------------

Sarcophilus_harrisii GGGAGTAGAGGTATATGTGTGTGTGTTGGAGCT-GGGGGGATTTGTCACG

Anolis_carolinensis ---------------------TGGGAAGTGGGGGAGA-------------

Xenopus_tropicalis ---------------------AGGGGAGATCCAGGGAAGAGA--------

Homo_sapiens ----------CGGGCCCCACAGACAAAAGCGCTTT-TCCTTGGCATTCCG

Gorilla_gorilla ----------CGGGCCCCACAGACAAAAGCGCTTT-TCCTTGGCATTCCG

Otolemur_garnettii ----------CCGGGCTCACAGACAAAAGCCTATA-CCCTTGGCATACCG

Mus_musculus ----------CGGGCCTCCCAGACAAAAGCGCATT-TCTTTGGCATTCCG

Rattus_norvegicus ----------CCGGCCTCCCAGACAAAAGCGCATT-TCTTTGGCATTCCG

Jaculus_jaculus ----------CAGACCTCTAACACAAAAGCTCATT-TCCCTGGCATTCTG

Cavia_porcellus ----------TGAGCCTCACAGACATAAGCGCTCT-TCCTTGGTATTCTG

Ochotona_princeps ----------TCAGACAAAAAAAAAAATGCTCTTT-CCTTCGGCATCCT-

Sorex_araneus GTCGCGGTTATCAGCTTTGCAGACAAAAGCGCTTT-CCTTTGGCAT----

Sus_scrofa ----------CGGGCCTCTCAGACAAAAGCTCTTTCCCCCTGGCATTCCG

Bos_taurus -------------CCACCTCAGACAAAAGCTCTTT-CCCTTGGCATTGCG

Tursiops_truncatus ----------CCGGCCTCACAGACAAAAGCTCTTT-CCCTTGGCATTCCG

Canis_familiaris ----------TCGGCCCCGCAGACAAAAGCGCTT--CCCCCGGCTTCCCG

Loxodonta_africana ----------CCCGCCTCACAGAC-AAAGCGCTTT-CCCTTGGCATTCCG

Procavia_capensis ----------CCGGCCTCACAGAC-AAAGCGCTTT-CCCCTGGTATTCCA

Choloepus_didactylus --------------------------------------------------

Myrmecophaga_tridactyla --------------------------------------------------

Macropus_eugenii --------------------------------------------------

Sarcophilus_harrisii GACTC---TCCCAACCCTGCAGACAAAAGCGGTTTTCGATTAGCATTCAG

Anolis_carolinensis ----------GGGAAGGCGCGGGGAGGGGCGGGTTGCAAACTGGACTGAA

Xenopus_tropicalis ----------TCGAGTCCACAGGGATCCAAAAGTCATTTGTAAGGATCTA

Homo_sapiens CATCCCTACCGA-CCCAGGGTTCCCGCGGGGCTGTCGGCGCTGCC-----

Gorilla_gorilla CATCCCTACCGA-CCCAGGGTTCCCGCGGGGCTGTCGGCGCTGCC-----

Otolemur_garnettii AATCCCCCACTGTCCCAGGGTACCCGCGGGGCTGCCTGCTCTGCC-----

Mus_musculus AATCCCCATCGA--CCCAGGGTTCCGCAGGACCGCCTGCACTGC------

Rattus_norvegicus AATCCCCATCGA--CCCAGGGTTCTGCAGGACCGCCTGCACTGC------

Jaculus_jaculus CATCCCCACCG--TCCCAGGGTTCCTCAGGGCCGCCTGCCCTGC------

Cavia_porcellus CATCCCCACTGA---CTGGGTTTCCCAGGGGCTGCCTGCTCTGCCTGTCT

Ochotona_princeps CATTACCACCGA---CCTACCTACCCCGGAGCTTCCGTGGGGGACTACC-

Sorex_araneus ---CCCCACTGACCCCCTGGTTCCTGCAGGGCTGCTGGCGCGACC-----

Sus_scrofa CATCCCCACCGA-CCCCAGGCTCCCGCGGGGCTGCCCGGACT--------

Bos_taurus CATCCCTACCGA-CCCCAGGTTCCCGCGGGGCTGCCCGCGCCGAC-----

Tursiops_truncatus CATCCCCAGCGA-CCCCAGGTTCCCGCGTGGCTGCCCGCGCTGCC-----

Canis_familiaris CACCCTCGCC---CCTCGCCCAGCCCAGGGCCCGGCGGGGCGGCC-----

Loxodonta_africana CATCTCCCTCGC-CCCCGGGTTCCCACGGAGCTGTT--------------

Procavia_capensis CATCCCCATGGA-CCCCGTGTTCCCGCGGGGCTGTC--------------

Choloepus_didactylus --------------------------------------------------

Myrmecophaga_tridactyla --------------------------------------------------

Macropus_eugenii --------------------------------------------------

Sarcophilus_harrisii CTTTCCAACTGC-----AGCTTGGCTCAGAGTGTGCTGTTGTGTTCATTC

Anolis_carolinensis CC------------------------------------------------

Xenopus_tropicalis AGGCCCTAATGTAATATATGGCGCCCACT---------------------

Homo_sapiens -------------CCATCTCCCCTCAGCTCGGCTCAGCTCGGTACCCGGG

Gorilla_gorilla -------------CCATCTCCCCTCAGCTCGGCTCAGCTCGGTACCCGGG

Otolemur_garnettii -------------CTGTCTCTCCTCATCTCAGAA---------AACCGGG

Mus_musculus -------------CTGTCTTGCCTCAGCTCAGCTGAGC-----ACCCCAG

Rattus_norvegicus -------------CTGTCTTGCCTCAGCTCAGCTGAGC-----ACCCCAG

Jaculus_jaculus -------------CTGGCTCCCCTCAGCTTAGCTGGGT-----ACCTGCC

Cavia_porcellus CCCTTCGGCTCAGCAAAGTTCATTTAGCTCTGCAGGGC-----ATCTAGG

Ochotona_princeps -------------CTGTCTCCCCTCATCTGGGTTTAAC-CAGCACCAGAG

Sorex_araneus -------------CCGCCTCCCCTCAGC---------------ACCGGGG

Sus_scrofa --------------------CCCTCGGTTCTGCTCCGC-----GCCGAGG

Bos_taurus -------------CGGACTCCCCTCAGTTCTGCTCAGC-----ACCGTGG

Tursiops_truncatus -------------CGGACTCCCCTCGGTTCTGCTCAGC-----ACCGAGG

Canis_familiaris -------------CGGGCTCCCTGCGTCCTGGCGGCCC-----GGCCCGG

Loxodonta_africana -------------CTATCTCTCCCCAGCTCTGCTCAGC-----ACCCGGG

Procavia_capensis -------------CTATCTCTACCCAGTTCCGCTCAGC-----ACCCGGG

Choloepus_didactylus --------------------------------------------------

Myrmecophaga_tridactyla --------------------------------------------------

Macropus_eugenii --------------------------------------------------

Sarcophilus_harrisii ACTTCTTCAG---TATGTCTCCCTAAATCCTCCGGGCG-----ACCCAAT

Anolis_carolinensis -------------TCTTCTTCTTGCAAATCTGTCAGACAAAGGCTTCTCC

Xenopus_tropicalis -------------CATACTCTTTTTATTTCCCAAACAAAATAGTGTATAG

Homo_sapiens GCCCAG---GGCAAGCTCCGCAGGACTTCCCCGGAGGGC-TGCGGCGTAC

Gorilla_gorilla GCCCAG---GGCAAGCTCCGTAGGGCTTCCCCGGAGGGC-CGCGGCGTAC

Otolemur_garnettii GCCCAG---GGCAAGCTGCACAGGGCTTCCCCCAAGGGC-TGCGGCATAG

Mus_musculus GCCCGG---GGCACATTCTTGTA---------------------------

Rattus_norvegicus GCCCGG---GGCACATTCTTGTA---------------------------

Jaculus_jaculus CCCCCACCCCGCATGCTCTTTAGGGCTTGCCCAGAGGGCTGTGGGCACTG

Cavia_porcellus GCCCAG---GGCTATCTCTGCAGAGTCATTTCATAGGCT-----------

Ochotona_princeps GCCTGA---AACAGGCT---------------------------------

Sorex_araneus TCCCAG---GGCCAGCTCCACGGGGGTTCCCCAGAGCCC-TGAGGAGCAA

Sus_scrofa TCCTAG---GGCAGGCTCCGCAGTGGTTCCCCAGAGGGCTTGCGGCGCAG

Bos_taurus T-CCGG---GACAAGTTCCGCAGGGGACCCCCAGACGGC-TGTGGCACAG

Tursiops_truncatus TCCCAG---GGCAGGCTCTCCGCAGGATCCCCAGAGGGC-TGTGGCATAG

Canis_familiaris GCTCAG---GGCGAGCTCCGCAGGCC--------------TGCGGTGCCG

Loxodonta_africana TCCCAG---GGCGAGCTCCGCAGGGGCTCCAGACGGC---TGCGGCGTGC

Procavia_capensis TTCCAG---GGCGAGCTCCGCAGGAACTCGAGGGC-----CGCTGAGTGC

Choloepus_didactylus --------------------------------------------------

Myrmecophaga_tridactyla --------------------------------------------------

Macropus_eugenii --------------------------------------------------

Sarcophilus_harrisii AACCCAGTGAAATCACCGTAGGGGGTCTAACTAAAATGATCTAGACACCA

Anolis_carolinensis GATTAG--------------------------------------------

Xenopus_tropicalis ACCTGT--------------------------------------------

Homo_sapiens -AGGCTGGCGCAGAACGAACCTTGGCCTG-GGCCGTATCT-----CCGGC

Gorilla_gorilla -AGGCTGGCGCAGAACGAACCTTGGCCTG-GGCCGTCTCT-----CCGGC

Otolemur_garnettii -ATGCTGGTGCTGAACGAACCTCGGCCTGAGGTTACCTCT-----CCAGC

Mus_musculus -GGGCGGGTACTGGATAAGTCTCAGCCTG-GGCTGCCTCT----CTCGGC

Rattus_norvegicus -GGGCGGGTACTGAATAAGCCTCAGTCTG-GGTTGCCTCT----CCCAGC

Jaculus_jaculus CAAGGCTCGGTTGGATGAATCTCAGCCTG-GGCTGCCTCTTAAGCTAGGC

Cavia_porcellus ------AGTGCTAAACTAAACTCAACCTG-GACCACCTCT-----CTGGT

Ochotona_princeps --GGCATGGAGCGAATCCGGCTGGGCCTG-GGTGGACTCT---CTTTCGC

Sorex_araneus --------------------ACCGGCCTG-----------------GGAC

Sus_scrofa -GGGCTGGTGCCGAGCGAA-CCCGGCCTG-GGCCGCCT-------CGGGA

Bos_taurus -GAGCTGGTGCTGAGCGAA-CCTGGCTTG-GGCCGCCTCT-----CAGAA

Tursiops_truncatus -GGGCCGGTGCTGAGCGAA-CCCGGCCCG-GGCTGCCTCT-----CAGGA

Canis_familiaris -GGGCTGCCGCGAA---GGCCCAGGGCCG-GGCGGCCTCT-----CCGGC

Loxodonta_africana TGG------------------------------------------CTGGC

Procavia_capensis TGTTTGTCTTCGGAAAGAAGCCCTGTCTG-GACCACCTCT-----TTGGT

Choloepus_didactylus --------------------------------------------------

Myrmecophaga_tridactyla --------------------------------------------------

Macropus_eugenii --------------------------------------------------

Sarcophilus_harrisii GGACTGTCTGATGGGCTAGGTGTGGGGAAGGAG------------AGAGC

Anolis_carolinensis -------TATGCAAACAGACCCTGGTCTTTAGCTCCTAGCAGAATTCAAA

Xenopus_tropicalis -------ATTTTGTCCATTAAGTGTTCTGTTTTGACCAATTTTGTTTAAA

Homo_sapiens TCCC-AGCCTCAGCGCGGCCCTCCCGAGTTAAGGTGGGCCCGGCCCGCGC

Gorilla_gorilla TCCC-AGCCTCAGCGCGGCCCTCCCGAGTTAAGGTGGGCCCGGCCCGCGC

Otolemur_garnettii TCCC-AGCCTCAGCTTGGACCGCCCAAGTTAAGCTGAGCCCCAGCGGCAC

Mus_musculus TCCG-AGCCCTAGTTCAGCTAGCCTGAGTTAAGGTGAGACAGACTGACTG

Rattus_norvegicus TCTG-AGCCCTAGCTCGGCTAACCTGAGTTAAGGTGAGACA----GACTG

Jaculus_jaculus TCCA-ACCCCTAGCTTGACCTGCCTGAGCTAAGGTGGGACCAGCCAGCTT

Cavia_porcellus CCCCAAACCCCTGCGAGGCCCACCTGAATTAAAGTGGGCCCAG--TCTGC

Ochotona_princeps TCCCAGGTCTTTGGGGCCCACCCTGAAGTGGCTGTGGACCGGTCTAGTGC

Sorex_araneus TCAC-ATCCCCAGGATGGCTGTCCCGGAGCTGAAGGGGGCCTTGGGGCGC

Sus_scrofa TCCC-TGCCTCAGCCTGGCCCGCCCGAGTTCAGGTGGGCCCTGCGGGGGC

Bos_taurus TTCC-TGCCCCAGCGTGGGCCGCCGGGGTTCAGGTGGGCCCTGCCGCCGC

Tursiops_truncatus TTCC-TGCCCCAGCGTGG-CCGCCGGGGTTCAGGTGGGCCCTGCGGGCGC

Canis_familiaris TTCC---CCCCGGCGCCGCCCGGCCGCGTGAAGGGGGGCCCCG--GGCGC

Loxodonta_africana TCCC-AGCCCCGGCGCGGCCTGCCCGAGTAAAGGTGGGCCC----CGCGC

Procavia_capensis TCCC-AGCCCCAGCGCCTTCTGCCCGAGTAAAGGTGGGCCC----GGCGC

Choloepus_didactylus --------------------------------------------------

Myrmecophaga_tridactyla --------------------------------------------------

Macropus_eugenii --------------------------------------------------

Sarcophilus_harrisii TCTTTGCCTTCGAAATCCTCTTCCCGAATCTCGCCGCCACCAGTCAAGGA

Anolis_carolinensis CATAAAGTTGTCA------TGGCCCGGGATCACGGGGTTGTTGTGTGTTT

Xenopus_tropicalis GAAA-AACTCTGGAAAAATTGTCTTGGTAACACCCTGCTCCATGTAATTT

Homo_sapiens CACAGGACCC-TCGC-CGGACC-----------CTCTAACCTCGCCCTCT

Gorilla_gorilla CACAGGACCC-TCGC-CGGACC-----------CTCTAACCTCGCCCTCT

Otolemur_garnettii CACAGACCCC-TGGCCCCCACT-----------CTCCAGCCTCGCCCTCT

Mus_musculus T---AACCCCTTCTCCCCGACC-----------TCC--GCCTCACCCTCT

Rattus_norvegicus T---AACCCCTTCTCCCCGACC-----------TCC--ACCTCACCCCCT

Jaculus_jaculus C---AGCCCT-TCACTCCGACC-----------TCC--ACCTCGCCCTCT

Cavia_porcellus TGCAGGCCCC-TCACCCCAACC-----------TCC--------TTTCCT

Ochotona_princeps CACGGGCCCCTGGCTCAGGCCC-----------TGCAAGCCTTGCTCTCT

Sorex_araneus CGCAGGCCC--GCACCCCAACC-----------CTCGGGCCTTGCCCGCT

Sus_scrofa CTCAGGCCCCTGCGCCCGGACC-----------CTCTGGCCTCGCCCTCT

Bos_taurus GGCAGGCCCC-ACGCCCGGACC-----------CTGCGGCCTCGCCCTGT

Tursiops_truncatus CTCAGGCCCC-GCGCCGGGACC-----------CTCCGGCCTCGCCCTCT

Canis_familiaris CCCAGGCCCC-GCGGCCCGACC-----------CCTCGGCCTC-CCCTGC

Loxodonta_africana TGAAGGCCCT-TTGCCCCGACC-----------CTCCGGCCTCGCCCTGT

Procavia_capensis CCTAGGCCAC-TTGCTCTGACC-----------CTCTGGCCTCGCCCTGT

Choloepus_didactylus --------------------------------------------------

Myrmecophaga_tridactyla --------------------------------------------------

Macropus_eugenii --------------------------------------------------

Sarcophilus_harrisii AGCAGCCAAGAACCCCCTACCC-----------CTAGCATTTTGTCCCCT

Anolis_carolinensis TCCGGGCTGTATGGCCATGTTC-----------CAGAAGTCCAGAAGAAT

Xenopus_tropicalis CCCGAGCGGCATTGTATTAGACTTGCAAGCTGTCTGGGGTCAAAGAGAGT

Homo_sapiens CCTTTGTTCCCGG-CTGGACGGGTTAGAC-AGCCAAAGGCT---------

Gorilla_gorilla CCTTTGTTCCCGG-CTGGACGGGTTAGAC-AGCCAAAGGCT---------

Otolemur_garnettii CCTTTGTTTCTGG-CTAGGCGATTCAAGC-AGCGGCAGACT---------

Mus_musculus TCTTTGTTCCTGC-CTAGGCTGACCAGCC---------------------

Rattus_norvegicus TCTTTGTTCCTGC-CTAGGCTGGCCAGGC---------------------

Jaculus_jaculus TCTTTGTTTCTGT-CTAGGCAGGCCAGGC-CGCTGCAGGCT---------

Cavia_porcellus CTTTTGTTTCCAG-CTAGGTGAACCAGAG-GACCCCAGACCTCA------

Ochotona_princeps CCCTTTGTTCCTGATGAGGCGGGCCAAAGCTGCTGCAAGGCTGACGGGAG

Sorex_araneus CCTTTGTTTCTGG-CGGGGCT-----------------------------

Sus_scrofa CCTTTGTTCCCGGTTTAGGCCGACCAGGC-GGCTGCGGGCTT--------

Bos_taurus CCTTTGTTCGGGC-TTAGGCGGGCCAGGCGGGCTGCCGGCTT--------

Tursiops_truncatus CCTTTGTTCCCGG-------------------CTGCGGGCTC--------

Canis_familiaris CCTTTGTTCCCGGCTTAGGCCGGC--------------------------

Loxodonta_africana CCTTTGTTCCTGG-CCGGACTGGCCAGGC-AGCCGCCGGCT---------

Procavia_capensis TCTTTGTTCCTGG-CTAGACTGGCCAGGC-AGCCACCGGCT---------

Choloepus_didactylus --------------------------------------------------

Myrmecophaga_tridactyla --------------------------------------------------

Macropus_eugenii --------------------------------------------------

Sarcophilus_harrisii TCATCTCTGGCTACATTTCCTGACCGAGA---------------------

Anolis_carolinensis ACTTCT--------------------------------------------

Xenopus_tropicalis TCTTTTGGGGGAGAAAATGCTAATTGAG----------------------

Homo_sapiens GGCGA--------------------GAGTCTGGCCCTAG----ACTCGGG

Gorilla_gorilla GGCGA--------------------GAGTCTGGCCCTAG----ACTCGGG

Otolemur_garnettii GGGGAGAGAGTGATTGTGGTTGTTGGAGTCCGTCTCCAG----A-TTGGG

Mus_musculus TGCTGAAGGCTGGTTGTGCCAGGCAGAGGCTATGACTAGCTAGAATCGGG

Rattus_norvegicus TGCTGAAGGCTGGTTGTGCCAGGCAGAGCCTATGGCCAG---AATCCGGG

Jaculus_jaculus GGCGAGAGAGTGG----------CAGAGTCTATGGCAAG----ACTC---

Cavia_porcellus GGCTGGCAGGTGCTTGTTACTGACAGAGTCCATTGTCA-----GCCTGGG

Ochotona_princeps AGTGAGAGAGTGCCTGAGCCGATGGGAACTGGGTCTGCA---ATCTTGGG

Sorex_araneus GGCGATGGAACGGTTGTGCTTGGTGGAGTCCGGCGCGA-----ATTCGGG

Sus_scrofa GGTGAGAGAACGGGTGTGCCTGGCCGAATCTGGCACCAG----ACTCGGG

Bos_taurus GGTGAGAGCATGGTTGTGCTTGGCAAACCCCGGCACCA-----TCTCGGG

Tursiops_truncatus GGTAAGAGGATGGTTGTGCCTGGCGGAGTCCGGCACCAG----ACTCGGG

Canis_familiaris -----GAGAGTGGTTGTGCTTGGCCGGGTC-GGCGCCGG----ACTCGGG

Loxodonta_africana GGCGAGAGAGTGGTTGTGCCT----GAACCCGGCGCCAG----ACTCGGG

Procavia_capensis GGTGGAAAAGTGGTTGTGCCT----GAACCCGGCGCCAG----ACTTGGG

Choloepus_didactylus --------------------------------------------------

Myrmecophaga_tridactyla --------------------------------------------------

Macropus_eugenii --------------------------------------------------

Sarcophilus_harrisii GGCTGGCAGTGGTCTGTTCGGGGTTGGG----------------------

Anolis_carolinensis GGAACATGGCCATACAGGCCCGGAAAAACCACACTACAACCCAATATAAA

Xenopus_tropicalis ATCTTTAGGCTGAAGAGGGCAGGGAAAGTGTGGCTGTA-----TTATATT

Homo_sapiens GTGCTT---------CCTTGTAGCGACTAAACTAGATTTTCACTTAT-GA

Gorilla_gorilla GTGCTT---------CCTTGTAGCGACTAAACTAGATTTTCACTTAT-GA

Otolemur_garnettii GTGCCT---------CCTTATAGCGACTAAACTCGATTTTCACTTATT-A

Mus_musculus GTGCTTTCAACTAACCTTTGTAACTTCTAAACT-GTCTTTGCTCATT--A

Rattus_norvegicus GTGCTTTCAACTAACCTTTATAACTTCTAAACTCGTCTTTGCTTATT--A

Jaculus_jaculus --------------------------------------------------

Cavia_porcellus GTACTT---------CTTCATGGTGACTAAGTTTAGTCTTCACTTATTAA

Ochotona_princeps GTGCTT---------CCTGTTAGAGATTTAACTGTATCTTCACTTAGT-G

Sorex_araneus GTGCTT----------CTGGTAGCGACTAAACTTGATCTTAATTTTTTGA

Sus_scrofa GTGCTT---------CCTTGTAGCGACTAAACTGATGTTCATTTTTAAAA

Bos_taurus GTACTT---------CTTTGTAGCCACTCAACTCGATGCTCATTTTTTAA

Tursiops_truncatus GTGCTT---------CCTTGTAGCCACTAAACTCGATGTTCATTTTTTAA

Canis_familiaris GTGCTT---------CCGTGTAGCGACTAAACTCCACGTTCGGTCTTTAA

Loxodonta_africana GTGCTT---------TCTTGTCGCGTCTAAACTCGAACTTCATTTTT--A

Procavia_capensis GTACTG---------CGTTGTCGCGTTTAAACTCGAACTTCATTTTT--A

Choloepus_didactylus --------------------------------------------------

Myrmecophaga_tridactyla --------------------------------------------------

Macropus_eugenii --------------------------------------------------

Sarcophilus_harrisii ----------------------------TGGCTTCAACTCCGTC------

Anolis_carolinensis GTTCTC-----TGGAGTTTTCTTCAACTCCCCTCCCCCCACTTTCTCTAA

Xenopus_tropicalis GTGCTTACTATAATAGGGGTCATAGATTTAATGGCCGGTTAGATATGGAA

Homo_sapiens ATGATTTGCATATGAAAGGAGAGCA---------------TCGGCCTAGG

Gorilla_gorilla ATGATTTGCATATGAAAGGAGAGCA---------------TCGGCCTAGG

Otolemur_garnettii ACGATTTGCATATGAAAGAGGAAAA-AGAC----TGAATCTCCGCCTAAG

Mus_musculus ATTATTTGCATATGAAAGAGGGAGA-TAAG----TGAAAGTGGGCCTGTA

Rattus_norvegicus ATGATTTGCATATGAAAGAGGGAGA-TAAG----TGAAAGTGGGCCTGTA

Jaculus_jaculus --------------------------------------------------

Cavia_porcellus --TGTTTGCATATGAAAGGGGGAGA------------------------A

Ochotona_princeps GTGTTCTGTCTGTAAAAGGGGGAGGTAGACAT--TGACGGCTACCCAGGG

Sorex_araneus ATGATTTACATATAAAAGGGGGACACCAC---------------------

Sus_scrofa ATGATTTGCATATGAAAGGGGGAGA-TGAC----TGAACCTCTGCCTAGG

Bos_taurus ATGATCTGCATATGAAAGGGGGAGA---------TGGCTGTCTGCCTAGG

Tursiops_truncatus ATGATTTGCATATGAAAGGGGGAAGATGAC----TGACTGTCTGCTTAGG

Canis_familiaris ATGATTTGCATATGAAAGGGGGGAGATGAC----TGAAGGCCCGCCTAGG

Loxodonta_africana ATGATTTGCATATGAAAGGGGGGAGATGTC----TGAACGTCAGCCTAGG

Procavia_capensis ATGATTTGCATATGAAAGGGGGGAGATGTC----TCAATGTCAGCCTAGG

Choloepus_didactylus --------------------------------------------------

Myrmecophaga_tridactyla --------------------------------------------------

Macropus_eugenii --------------------------------------------------

Sarcophilus_harrisii --------------------------------------------------

Anolis_carolinensis ATTGGGAGGAAATATCAGAACCAGTCTTTTAA--TTAGCTAAGGCATTAA

Xenopus_tropicalis ATATATTAAAAACGAGGAGAAGAGGTTCTTAAGTTAAACTTCTCCATTCG

Homo_sapiens GCCCCCACAGTTGCTCTATGCTTTCCAAACCTTATCTCCACAACCTCTTC

Gorilla_gorilla GCCCCCACAGTTGCTCTATGCTTTCCAAACCTTATCTCCACAACCTCTTC

Otolemur_garnettii GGCCCATCGATTTCTCT---------------------------------

Mus_musculus ACCCCGGAGGTTGTCCTATGCTCTCTATACCTTCCAATC---------CC

Rattus_norvegicus GCCCTGGAGGTTGTCCTATGCTCTCTAAACCTTCCAACC---------CC

Jaculus_jaculus --------------------------------------------------

Cavia_porcellus GACTGATAGGTTGCCCTATCCTCTCCACATTTTCTTCCC---------CT

Ochotona_princeps GTCCCCACAGTTGCTCTGCACTCGCTTTTGGAAACACCCTTCTGAAACCT

Sorex_araneus --------------------------------------------------

Sus_scrofa GCCCCCTCCATTGCCCTATGCTCTTCAAACCTGCTCCCC---------CA

Bos_taurus GCCCCCTCGGTTGCCCTATGCTCTCTA-----------------------

Tursiops_truncatus GCGCCCTTGGTTGCCCTATGCTCTCCCAACCTGCGCCCC---------CC

Canis_familiaris GCCCCCCCGGTTGCCCTAGGCTCTCCAAACCTTCTCAGC---------CA

Loxodonta_africana CCCCCCTCTGTTGCCCTATGCTCTTCAAACCTTCTTTCC---------TC

Procavia_capensis GCCCCCTCTGTTGCCCTATGCTCTTCAAACCTTCTTTCC---------TC

Choloepus_didactylus --------------------------------------------------

Myrmecophaga_tridactyla --------------------------------------------------

Macropus_eugenii --------------------------------------------------

Sarcophilus_harrisii --------------------------------------------------

Anolis_carolinensis GCCCTTAAATATGA---------GAATGATCGTGAGACTCAAAAGCTTAT

Xenopus_tropicalis TTCCATCCAAATGGAATAGACCCGAGTGTGTGTACAATCCACACATTAAT

Homo_sapiens CCCCCAAAACCCGGGAACCTCCCCAGCCTGCGCCTGCTGCATGCCCTCTC

Gorilla_gorilla CCCCCAAAACCCGGGAACCTCCCCAGCCTGCGCCTGCTGCATGCCCTCTC

Otolemur_garnettii --------------------------------------------------

Mus_musculus CTTCCCAAAGCCCGGGAACTTCCCTAGCCTGGCTTGCTGCATGCACTCTC

Rattus_norvegicus CTCCCTAAAGCCCGGGATCTTCCCTAGCCTGGCTTGCTGCATGCACTCTC

Jaculus_jaculus --------------------------------------------------

Cavia_porcellus ACCCCAAA------------------------------------------

Ochotona_princeps TGGGAGAAGCCCCGAGGACTCCCCTCCCCAGCCCAGCCCACTGCATGCTC

Sorex_araneus --------------------------------------------------

Sus_scrofa CCCCAAAGCCCGGGGAACCTCTCCAGCCTGCACTCTCTGCATGCTCTCTC

Bos_taurus --------------------------------------------------

Tursiops_truncatus ACCC----------------------------------------------

Canis_familiaris CCCCTCAGCCCC-GTAACGTCTCCAGCCTG--------------------

Loxodonta_africana CGGGAGAACCTAGGAACCTCCCCAGCCCTCGCCAAGCTGCATGCCCGCTC

Procavia_capensis CAGGAAAGC-----------------------------------------

Choloepus_didactylus --------------------------------------------------

Myrmecophaga_tridactyla --------------------------------------------------

Macropus_eugenii --------------------------------------------------

Sarcophilus_harrisii --------------------------------------------------

Anolis_carolinensis GGGAAAAAAACCCGG-----------------------------------

Xenopus_tropicalis CATTTCATGCACTTGTATAGTC----------------------------

Homo_sapiens -AGGCCGGCAGCCCCAGCCTGCTAGCTAGCTCAACTAGTGGGGTTTCCTG

Gorilla_gorilla -AGGCCGGCAGCCCCAGCCTGCTAGCTAGCTCAACTATTGGGGTTTCCTG

Otolemur_garnettii --------------------------------------------------

Mus_musculus -AGGTTCACAGTTCCAGCCTGCTAGCTAGCTCAACTATTGGGGTCTCTTG

Rattus_norvegicus -AGGTTCACAGTCCCAGCCTGCTAGCTAGCTCAACTATTGGGGTCTCTTG

Jaculus_jaculus --------------------------------------------------

Cavia_porcellus --------------------------------------------------

Ochotona_princeps TCAGGCCTCAGCCCCAGCCTGCTAGCTAGTGCAAATGTTGGGGTGTCCTG

Sorex_araneus --------------------------------------------------

Sus_scrofa -AGGCCCCCAGCCTCAGGCCGCTAGCTAGCTCAACTATTGGGGTTCTCTG

Bos_taurus --------------------------------------------------

Tursiops_truncatus --------------------------------------------------

Canis_familiaris --------------------------------------------------

Loxodonta_africana -AGGCCCGCAGCCCCAGCCC----GCTAGCTCCACTGTTGGGGTCACCTG

Procavia_capensis --------------------------------------------------

Choloepus_didactylus --------------------------------------------------

Myrmecophaga_tridactyla --------------------------------------------------

Macropus_eugenii --------------------------------------------------

Sarcophilus_harrisii --------------------------------------------------

Anolis_carolinensis --------------------------------------------------

Xenopus_tropicalis --------------------------------------------------

Homo_sapiens GCACTGGACCCCAGCAAGTGG-TCCTAGAGGCCCTTTGCTGTCCCATAGT

Gorilla_gorilla GCACTGGACCCCAGCAAGTGG-TCCTAGAGGCCCTTTGCTGTCCCATAGT

Otolemur_garnettii --------------------------------------------------

Mus_musculus TCTTTGGACCCTAGCAAGTTGTTCTTTGAGGCTTTTTGCTATCCTATATA

Rattus_norvegicus CCTTTGGACCCTAGCAAGTGGTTCTTTGAAGCTTTTTGCTATCCTATGTA

Jaculus_jaculus --------------------------------------------------

Cavia_porcellus --------------------------------------------------

Ochotona_princeps CCA-TGGAACCCAGCAAACGTTCCTACAGGCCCCTTTGCGGGTCCACAGT

Sorex_araneus --------------------------------------------------

Sus_scrofa CTACTGG-------------------------------------------

Bos_taurus --------------------------------------------------

Tursiops_truncatus --------------------------------------------------

Canis_familiaris --------------------------------------------------

Loxodonta_africana CCACTGGACCTCAGTAAGCAG-TCCCAGAGGCTTTTGGTTGCCCTATAGT

Procavia_capensis --------------------------------------------------

Choloepus_didactylus --------------------------------------------------

Myrmecophaga_tridactyla --------------------------------------------------

Macropus_eugenii --------------------------------------------------

Sarcophilus_harrisii --------------------------------------------------

Anolis_carolinensis --------------------------------------------------

Xenopus_tropicalis --------------------------------------------------

Homo_sapiens CCCTGCCACGAATTTCTGTGCCCTCCTGACCCATTGCTGTTGTCCAACTA

Gorilla_gorilla CCCTGCCACGAATTTCTGTGCCCTCCTGACCCATTGCTGTTGTCCAACTA

Otolemur_garnettii --------------------------------------------------

Mus_musculus TCTTGCCATGACTTTCTCACCTTCCTG-CCCCATTGCAGTTGCCCAACTA

Rattus_norvegicus TCTTGCCATGACTTTCTCACCTTCCTGCCCCCATTGCAGTTGCCCAACTA

Jaculus_jaculus --------------------------------------------------

Cavia_porcellus --------------------------------------------------

Ochotona_princeps CCCTGCCAC---TCTCTGTGCCCTCCTGCTCCATCACTGATGTCCAACTA

Sorex_araneus --------------------------------------------------

Sus_scrofa --------------------------------------------------

Bos_taurus --------------------------------------------------

Tursiops_truncatus --------------------------------------------------

Canis_familiaris --------------------------------------------------

Loxodonta_africana CCCTGCCACG----------------------------------------

Procavia_capensis --------------------------------------------------

Choloepus_didactylus --------------------------------------------------

Myrmecophaga_tridactyla --------------------------------------------------

Macropus_eugenii --------------------------------------------------

Sarcophilus_harrisii --------------------------------------------------

Anolis_carolinensis --------------------------------------------------

Xenopus_tropicalis --------------------------------------------------

Homo_sapiens TTTATTGACTCTGGGTCCTTCCT---------------------------

Gorilla_gorilla TTTATTGACTCTGGGTCCTTCCTGAAACTATATTTTGTCATAT--CAAAT

Otolemur_garnettii --------------------------------------------------

Mus_musculus TTTATTGACTCCAAGCCCTTCCTGAAACTATATTTTGTCATAT--CAAAT

Rattus_norvegicus TTTATTGACTCCAAGTCCTTCCTGAAACTATATTTTGTCATAT--CAAAT

Jaculus_jaculus --------------------------------------------------

Cavia_porcellus --------------------------------------------------

Ochotona_princeps TTTATTGACTGCGGGTCCTTCCTGAAACTATATTTTCTCATATATCAAAT

Sorex_araneus --------------------------------------------------

Sus_scrofa --------------------------------------------------

Bos_taurus --------------------------------------------------

Tursiops_truncatus --------------------------------------------------

Canis_familiaris --------------------------------------------------

Loxodonta_africana --------------------------------------------------

Procavia_capensis --------------------------------------------------

Choloepus_didactylus --------------------------------------------------

Myrmecophaga_tridactyla --------------------------------------------------

Macropus_eugenii --------------------------------------------------

Sarcophilus_harrisii --------------------------------------------------

Anolis_carolinensis --------------------------------------------------

Xenopus_tropicalis --------------------------------------------------

Homo_sapiens --------------------------------------------------

Gorilla_gorilla AAAG------AGAGAACAGGACTAAAGATGCAGTGGCTCCTGTCTGTTTG

Otolemur_garnettii --------------------------------------------------

Mus_musculus AAAGCCAGAAAAAGAACAGGACTAAAGATTCAATTACTTCTGTCTGTTTA

Rattus_norvegicus AAAGCCAGAAAAAGAACAGGACTAAAGATTCAATGACTTCTGTCTGTTTA

Jaculus_jaculus --------------------------------------------------

Cavia_porcellus --------------------------------------------------

Ochotona_princeps AAAGCTGAAGAGAGAACGGGGCTCAAAGTGCAGTGGCTCCCGTCTGTTTG

Sorex_araneus --------------------------------------------------

Sus_scrofa --------------------------------------------------

Bos_taurus --------------------------------------------------

Tursiops_truncatus --------------------------------------------------

Canis_familiaris --------------------------------------------------

Loxodonta_africana --------------------------------------------------

Procavia_capensis --------------------------------------------------

Choloepus_didactylus --------------------------------------------------

Myrmecophaga_tridactyla --------------------------------------------------

Macropus_eugenii --------------------------------------------------

Sarcophilus_harrisii --------------------------------------------------

Anolis_carolinensis --------------------------------------------------

Xenopus_tropicalis --------------------------------------------------

Homo_sapiens --------------------------------------------------

Gorilla_gorilla GGGCATGTATTGGGTAAAATTGTCTAAATGGGCTGTGAGATGTAGAAGGA

Otolemur_garnettii --------------------------------------------------

Mus_musculus GGGCATGCATTGAATTGATGTGTCTAAATAGGCTCTGAGATGTATTGGGA

Rattus_norvegicus GGGCATGCATTGAGTAGATGTGTGTAAATAGGCTCTGAGATGTATGGGGA

Jaculus_jaculus --------------------------------------------------

Cavia_porcellus --------------------------------------------------

Ochotona_princeps GGGCATGTGTTTGGGGTGAAAGTGTCTAAACGGGGTGGTAAGATGTAGGA

Sorex_araneus --------------------------------------------------

Sus_scrofa --------------------------------------------------

Bos_taurus --------------------------------------------------

Tursiops_truncatus --------------------------------------------------

Canis_familiaris --------------------------------------------------

Loxodonta_africana --------------------------------------------------

Procavia_capensis --------------------------------------------------

Choloepus_didactylus --------------------------------------------------

Myrmecophaga_tridactyla --------------------------------------------------

Macropus_eugenii --------------------------------------------------

Sarcophilus_harrisii --------------------------------------------------

Anolis_carolinensis --------------------------------------------------

Xenopus_tropicalis --------------------------------------------------

Homo_sapiens --------------------------------------------------

Gorilla_gorilla GACCCACAAACTTTAAGCCACCCCTTTAAAAAATTTTCTAGCTTCTAGGG

Otolemur_garnettii --------------------------------------------------

Mus_musculus GGCCCAGTCTTTAAACCATCCCTTAAAAAATATTTTTAAACACCTTTAGG

Rattus_norvegicus GGCCCTGTCTCTAAGCCACCCCTTTAAAAATATTTTTAAGCACGTTTAGG

Jaculus_jaculus --------------------------------------------------

Cavia_porcellus --------------------------------------------------

Ochotona_princeps AGAGGCCTACAGTCTTTAAGACTCTCT-----------------------

Sorex_araneus --------------------------------------------------

Sus_scrofa --------------------------------------------------

Bos_taurus --------------------------------------------------

Tursiops_truncatus --------------------------------------------------

Canis_familiaris --------------------------------------------------

Loxodonta_africana --------------------------------------------------

Procavia_capensis --------------------------------------------------

Choloepus_didactylus --------------------------------------------------

Myrmecophaga_tridactyla --------------------------------------------------

Macropus_eugenii --------------------------------------------------

Sarcophilus_harrisii --------------------------------------------------

Anolis_carolinensis --------------------------------------------------

Xenopus_tropicalis --------------------------------------------------

Homo_sapiens --------------------------------------------------

Gorilla_gorilla GCAGTCAGTAGAGGGAGGTGAAATTAAGCTCCTTTCCTGTTCCTTGGCTG

Otolemur_garnettii --------------------------------------------------

Mus_musculus AGCAGCAAGAATAGGGAACAATGGAACTGAGTTCTTTTCTTGATTCATGG

Rattus_norvegicus ATCAGCAAGGATAGGGAAAAACTGAG------TGCCTTTTTGATTCATGG

Jaculus_jaculus --------------------------------------------------

Cavia_porcellus --------------------------------------------------

Ochotona_princeps --------------------------------------------------

Sorex_araneus --------------------------------------------------

Sus_scrofa --------------------------------------------------

Bos_taurus --------------------------------------------------

Tursiops_truncatus --------------------------------------------------

Canis_familiaris --------------------------------------------------

Loxodonta_africana --------------------------------------------------

Procavia_capensis --------------------------------------------------

Choloepus_didactylus --------------------------------------------------

Myrmecophaga_tridactyla --------------------------------------------------

Macropus_eugenii --------------------------------------------------

Sarcophilus_harrisii --------------------------------------------------

Anolis_carolinensis --------------------------------------------------

Xenopus_tropicalis --------------------------------------------------

Homo_sapiens --------------------------------------------------

Gorilla_gorilla CAACAAGAGCCTCTGGGGGAAGAGAAGGGCACCTAGGCCTCGGGTACTGG

Otolemur_garnettii --------------------------------------------------

Mus_musculus TTCCAAGAAGGGCCTCTGGGGTGAAGGGGAGTACACTTGAGGACTCCTGT

Rattus_norvegicus TTCCAAGAAGGGCCTCTGGGGTGAAGGGGAGTACACTGGAGAGCTCCTGT

Jaculus_jaculus --------------------------------------------------

Cavia_porcellus --------------------------------------------------

Ochotona_princeps --------------------------------------------------

Sorex_araneus --------------------------------------------------

Sus_scrofa --------------------------------------------------

Bos_taurus --------------------------------------------------

Tursiops_truncatus --------------------------------------------------

Canis_familiaris --------------------------------------------------

Loxodonta_africana --------------------------------------------------

Procavia_capensis --------------------------------------------------

Choloepus_didactylus --------------------------------------------------

Myrmecophaga_tridactyla --------------------------------------------------

Macropus_eugenii --------------------------------------------------

Sarcophilus_harrisii --------------------------------------------------

Anolis_carolinensis --------------------------------------------------

Xenopus_tropicalis --------------------------------------------------

Homo_sapiens --------------------------------------------------

Gorilla_gorilla CTTTTC-----------------CCATGCTGTGGCTGTCCAAGGCCTTCA

Otolemur_garnettii --------------------------------------------------

Mus_musculus ACTGTT--------GTTTCCACTTGCTGT-GTGTGTCTAAGTTACCCAGA

Rattus_norvegicus ACTGTTGTACTGTCGTTTCCACATGATGTGGGGTGTACAAGGCACCCAGA

Jaculus_jaculus --------------------------------------------------

Cavia_porcellus --------------------------------------------------

Ochotona_princeps --------------------------------------------------

Sorex_araneus --------------------------------------------------

Sus_scrofa --------------------------------------------------

Bos_taurus --------------------------------------------------

Tursiops_truncatus --------------------------------------------------

Canis_familiaris --------------------------------------------------

Loxodonta_africana --------------------------------------------------

Procavia_capensis --------------------------------------------------

Choloepus_didactylus --------------------------------------------------

Myrmecophaga_tridactyla --------------------------------------------------

Macropus_eugenii --------------------------------------------------

Sarcophilus_harrisii --------------------------------------------------

Anolis_carolinensis --------------------------------------------------

Xenopus_tropicalis --------------------------------------------------

Homo_sapiens --------------------------------------------------

Gorilla_gorilla CGCCTCTGCACTCCATGAGGCCTCTGGACTGGCAGCAGCCCCCACTGTAT

Otolemur_garnettii --------------------------------------------------

Mus_musculus CTTTTCTATATCCTATTCTCAACGTTGGCTAAAACAACTTGAGACTTGAA

Rattus_norvegicus CTTTTCTATATCTTATTCTC-ATGTTGGCTAAAAAGACTCGAGGCTTCTA

Jaculus_jaculus --------------------------------------------------

Cavia_porcellus --------------------------------------------------

Ochotona_princeps --------------------------------------------------

Sorex_araneus --------------------------------------------------

Sus_scrofa --------------------------------------------------

Bos_taurus --------------------------------------------------

Tursiops_truncatus --------------------------------------------------

Canis_familiaris --------------------------------------------------

Loxodonta_africana --------------------------------------------------

Procavia_capensis --------------------------------------------------

Choloepus_didactylus --------------------------------------------------

Myrmecophaga_tridactyla --------------------------------------------------

Macropus_eugenii --------------------------------------------------

Sarcophilus_harrisii --------------------------------------------------

Anolis_carolinensis --------------------------------------------------

Xenopus_tropicalis --------------------------------------------------

Homo_sapiens --------------------------------------------------

Gorilla_gorilla GCCAAAGCTGTGGTGCAGGAACCAGTGGCTCCGCCGTGCTCAGCCTTAGG

Otolemur_garnettii --------------------------------------------------

Mus_musculus GCTTCTAGACTGTAGTCTGTACTTTATGCCAGAGAATAGTAGCTCTGTGG

Rattus_norvegicus GACTTT---CTGTAGTCTGTGCTTTATGTCAGAGCAAAGGAACT-TGTGG

Jaculus_jaculus --------------------------------------------------

Cavia_porcellus --------------------------------------------------

Ochotona_princeps --------------------------------------------------

Sorex_araneus --------------------------------------------------

Sus_scrofa --------------------------------------------------

Bos_taurus --------------------------------------------------

Tursiops_truncatus --------------------------------------------------

Canis_familiaris --------------------------------------------------

Loxodonta_africana --------------------------------------------------

Procavia_capensis --------------------------------------------------

Choloepus_didactylus --------------------------------------------------

Myrmecophaga_tridactyla --------------------------------------------------

Macropus_eugenii --------------------------------------------------

Sarcophilus_harrisii --------------------------------------------------

Anolis_carolinensis --------------------------------------------------

Xenopus_tropicalis --------------------------------------------------

Homo_sapiens --------------------------------------------------

Gorilla_gorilla CCCTTCTGGGCCTCTGAAGATCTTTCCTGGGCCTAGGTGGTCCCTGAGAG

Otolemur_garnettii --------------------------------------------------

Mus_musculus CTCCACTGACTTAGATTCAGGCTCTTCTGGGTCTAGGTCATCCTTGTAGG

Rattus_norvegicus CTCCACTGACTTAGATTCAGGCCCTTCTGGGTCTAGGTTATCCCTGGGGT

Jaculus_jaculus --------------------------------------------------

Cavia_porcellus --------------------------------------------------

Ochotona_princeps --------------------------------------------------

Sorex_araneus --------------------------------------------------

Sus_scrofa --------------------------------------------------

Bos_taurus --------------------------------------------------

Tursiops_truncatus --------------------------------------------------

Canis_familiaris --------------------------------------------------

Loxodonta_africana --------------------------------------------------

Procavia_capensis --------------------------------------------------

Choloepus_didactylus --------------------------------------------------

Myrmecophaga_tridactyla --------------------------------------------------

Macropus_eugenii --------------------------------------------------

Sarcophilus_harrisii --------------------------------------------------

Anolis_carolinensis --------------------------------------------------

Xenopus_tropicalis --------------------------------------------------

Homo_sapiens --------------------------------------------------

Gorilla_gorilla GCCTTGCTGGACATAGAGGGAGGGCTGAGCTCTCACTCTAGGGACCCATT

Otolemur_garnettii --------------------------------------------------

Mus_musculus TCCTTTCT-GACATAGAAGAAAGGTTGAGTCCTCTGTCTAG---------

Rattus_norvegicus TCTTTTCTGGACATGGAAGAACGGCTGGGTCCTCAGTCTTGGGCTATGTT

Jaculus_jaculus --------------------------------------------------

Cavia_porcellus --------------------------------------------------

Ochotona_princeps --------------------------------------------------

Sorex_araneus --------------------------------------------------

Sus_scrofa --------------------------------------------------

Bos_taurus --------------------------------------------------

Tursiops_truncatus --------------------------------------------------

Canis_familiaris --------------------------------------------------

Loxodonta_africana --------------------------------------------------

Procavia_capensis --------------------------------------------------

Choloepus_didactylus --------------------------------------------------

Myrmecophaga_tridactyla --------------------------------------------------

Macropus_eugenii --------------------------------------------------

Sarcophilus_harrisii --------------------------------------------------

Anolis_carolinensis --------------------------------------------------

Xenopus_tropicalis --------------------------------------------------

Homo_sapiens --------------------------------------------------

Gorilla_gorilla TGGTCTCATAAGGGCCTCTAGAGAGAGGTGGTATTGCTTGGTAAGGGGGT

Otolemur_garnettii --------------------------------------------------

Mus_musculus -ATGCCAGAAGGATTCGCAGCAATAGTAGGTGCTGCTTGGTAAAGAGGTG

Rattus_norvegicus TGATCTCAGAGGATTCTCAGCAATCGTAGGCAGTACTTGGTAAAGGGGTT

Jaculus_jaculus --------------------------------------------------

Cavia_porcellus --------------------------------------------------

Ochotona_princeps --------------------------------------------------

Sorex_araneus --------------------------------------------------

Sus_scrofa --------------------------------------------------

Bos_taurus --------------------------------------------------

Tursiops_truncatus --------------------------------------------------

Canis_familiaris --------------------------------------------------

Loxodonta_africana --------------------------------------------------

Procavia_capensis --------------------------------------------------

Choloepus_didactylus --------------------------------------------------

Myrmecophaga_tridactyla --------------------------------------------------

Macropus_eugenii --------------------------------------------------

Sarcophilus_harrisii --------------------------------------------------

Anolis_carolinensis --------------------------------------------------

Xenopus_tropicalis --------------------------------------------------

Homo_sapiens ---------------GAAACTATATTTTGTCATATCAAATA------AAG

Gorilla_gorilla GTTGGGAGAGTTGGAGGAGGTGTGCCTCAGTATCTAAGATACTGGGGAAG

Otolemur_garnettii --------------------------------------------------

Mus_musculus TTGGGGAAGGAGAAATGGCCACGGTTTGAGATGTTCTTCAGCCTTCTAAG

Rattus_norvegicus TTGGAGAAGAGGAGTTGACCTTGGTTTGAGATGTTCTTCAGCTTTCTAAG

Jaculus_jaculus --------------------------------------------------

Cavia_porcellus --------------------------------------------------

Ochotona_princeps --------------------------------------------------

Sorex_araneus --------------------------------------------------

Sus_scrofa --------------------------------------------------

Bos_taurus --------------------------------------------------

Tursiops_truncatus --------------------------------------------------

Canis_familiaris --------------------------------------------------

Loxodonta_africana --------------------------------------------------

Procavia_capensis --------------------------------------------------

Choloepus_didactylus --------------------------------------------------

Myrmecophaga_tridactyla --------------------------------------------------

Macropus_eugenii --------------------------------------------------

Sarcophilus_harrisii --------------------------------------------------

Anolis_carolinensis --------------------------------------------------

Xenopus_tropicalis --------------------------------------------------

Homo_sapiens AGAGAACAGGA-------------------------CTAA----------

Gorilla_gorilla AGGGAGCAGGGAGGACAATCTGCAGTGTGTATCAGCCTGG----------

Otolemur_garnettii --------------------------------------------------

Mus_musculus ATATGGGATGGGGCAAGGGGGACTAAGGAGACAGTGAATGCAACCTGCAG

Rattus_norvegicus ACATGGGATGGGGCAAGGGGGACTAGGGGGACACTGAATGCAACCTGCAG

Jaculus_jaculus --------------------------------------------------

Cavia_porcellus --------------------------------------------------

Ochotona_princeps --------------------------------------------------

Sorex_araneus --------------------------------------------------

Sus_scrofa --------------------------------------------------

Bos_taurus --------------------------------------------------

Tursiops_truncatus --------------------------------------------------

Canis_familiaris --------------------------------------------------

Loxodonta_africana --------------------------------------------------

Procavia_capensis --------------------------------------------------

Choloepus_didactylus --------------------------------------------------

Myrmecophaga_tridactyla --------------------------------------------------

Macropus_eugenii --------------------------------------------------

Sarcophilus_harrisii --------------------------------------------------

Anolis_carolinensis --------------------------------------------------

Xenopus_tropicalis --------------------------------------------------

Homo_sapiens --------------------------------------------------

Gorilla_gorilla --------------------------------------------------

Otolemur_garnettii --------------------------------------------------

Mus_musculus TGTGTGCCTGCCTTAGAGAAAACATACAGACTGCTCCCTGTCTTCTAGGG

Rattus_norvegicus TGTGTGCTTGCCTTAGAGAAAACATACAAACTGCTCCCTGTCTTCTAAGG

Jaculus_jaculus --------------------------------------------------

Cavia_porcellus --------------------------------------------------

Ochotona_princeps --------------------------------------------------

Sorex_araneus --------------------------------------------------

Sus_scrofa --------------------------------------------------

Bos_taurus --------------------------------------------------

Tursiops_truncatus --------------------------------------------------

Canis_familiaris --------------------------------------------------

Loxodonta_africana --------------------------------------------------

Procavia_capensis --------------------------------------------------

Choloepus_didactylus --------------------------------------------------

Myrmecophaga_tridactyla --------------------------------------------------

Macropus_eugenii --------------------------------------------------

Sarcophilus_harrisii --------------------------------------------------

Anolis_carolinensis --------------------------------------------------

Xenopus_tropicalis --------------------------------------------------

Homo_sapiens --------------------------------------------------

Gorilla_gorilla --------------------------------------------------

Otolemur_garnettii --------------------------------------------------

Mus_musculus TCTAAGCCAATGAAGGACAGTTAACTGGAATAAGAGCTGTATAGATCAGT

Rattus_norvegicus TCTAAGCCAATGCAGGGCAGTTAACTGGAGTAAGAGCGGTGTAGACCAGG

Jaculus_jaculus --------------------------------------------------

Cavia_porcellus --------------------------------------------------

Ochotona_princeps --------------------------------------------------

Sorex_araneus --------------------------------------------------

Sus_scrofa --------------------------------------------------

Bos_taurus --------------------------------------------------

Tursiops_truncatus --------------------------------------------------

Canis_familiaris --------------------------------------------------

Loxodonta_africana --------------------------------------------------

Procavia_capensis --------------------------------------------------

Choloepus_didactylus --------------------------------------------------

Myrmecophaga_tridactyla --------------------------------------------------

Macropus_eugenii --------------------------------------------------

Sarcophilus_harrisii --------------------------------------------------

Anolis_carolinensis --------------------------------------------------

Xenopus_tropicalis --------------------------------------------------

Homo_sapiens --------------------------------------------------

Gorilla_gorilla --------------------------------------------------

Otolemur_garnettii --------------------------------------------------

Mus_musculus GTGGGAGCCATGCCTGTTTGCTGTCATCTATCATA-TCTCTGAGATTTTG

Rattus_norvegicus GCAGGATCCATGCCTATT---TGTCATCTATCACAGTCTCTGAGATTTTG

Jaculus_jaculus --------------------------------------------------

Cavia_porcellus --------------------------------------------------

Ochotona_princeps --------------------------------------------------

Sorex_araneus --------------------------------------------------

Sus_scrofa --------------------------------------------------

Bos_taurus --------------------------------------------------

Tursiops_truncatus --------------------------------------------------

Canis_familiaris --------------------------------------------------

Loxodonta_africana --------------------------------------------------

Procavia_capensis --------------------------------------------------

Choloepus_didactylus --------------------------------------------------

Myrmecophaga_tridactyla --------------------------------------------------

Macropus_eugenii --------------------------------------------------

Sarcophilus_harrisii --------------------------------------------------

Anolis_carolinensis --------------------------------------------------

Xenopus_tropicalis --------------------------------------------------

Homo_sapiens --------------------------------------------------

Gorilla_gorilla --------------------------------------------------

Otolemur_garnettii --------------------------------------------------

Mus_musculus TATACAGGGGGAGGGTGCCCTATAGGAGAGGGTATGCAGCTAATTCA---

Rattus_norvegicus TATAAATGGGGAGGGTGGCTGATAGGAGAGGGGACACAGCTAATTCACAG

Jaculus_jaculus --------------------------------------------------

Cavia_porcellus --------------------------------------------------

Ochotona_princeps --------------------------------------------------

Sorex_araneus --------------------------------------------------

Sus_scrofa --------------------------------------------------

Bos_taurus --------------------------------------------------

Tursiops_truncatus --------------------------------------------------

Canis_familiaris --------------------------------------------------

Loxodonta_africana --------------------------------------------------

Procavia_capensis --------------------------------------------------

Choloepus_didactylus --------------------------------------------------

Myrmecophaga_tridactyla --------------------------------------------------

Macropus_eugenii --------------------------------------------------

Sarcophilus_harrisii --------------------------------------------------

Anolis_carolinensis --------------------------------------------------

Xenopus_tropicalis --------------------------------------------------

Homo_sapiens --------------------------------------------------

Gorilla_gorilla --------------------------------------------------

Otolemur_garnettii --------------------------------------------------

Mus_musculus --GGTGGGGCTGGCCTAGGCTGATGCGCTGCTTAGCTCCACTCCAGGCTC

Rattus_norvegicus GGGGCGGGGCTGGCCTAGGCTCATGCTCTTGCCAGCTCCACTCCAGGGTC

Jaculus_jaculus --------------------------------------------------

Cavia_porcellus --------------------------------------------------

Ochotona_princeps --------------------------------------------------

Sorex_araneus --------------------------------------------------

Sus_scrofa --------------------------------------------------

Bos_taurus --------------------------------------------------

Tursiops_truncatus --------------------------------------------------

Canis_familiaris --------------------------------------------------

Loxodonta_africana --------------------------------------------------

Procavia_capensis --------------------------------------------------

Choloepus_didactylus --------------------------------------------------

Myrmecophaga_tridactyla --------------------------------------------------

Macropus_eugenii --------------------------------------------------

Sarcophilus_harrisii --------------------------------------------------

Anolis_carolinensis --------------------------------------------------

Xenopus_tropicalis --------------------------------------------------

Homo_sapiens --------------------------------------------------

Gorilla_gorilla --------------------------------------------------

Otolemur_garnettii --------------------------------------------------

Mus_musculus CAGGACTTGTTGAAGGAGGAAGTACTTTACAGGAGAAACAAACGCACGGA

Rattus_norvegicus CACGA---GATGGAGGAGGGAGTACTTTACAGGAGAAACAAATG--CGGT

Jaculus_jaculus --------------------------------------------------

Cavia_porcellus --------------------------------------------------

Ochotona_princeps --------------------------------------------------

Sorex_araneus --------------------------------------------------

Sus_scrofa --------------------------------------------------

Bos_taurus --------------------------------------------------

Tursiops_truncatus --------------------------------------------------

Canis_familiaris --------------------------------------------------

Loxodonta_africana --------------------------------------------------

Procavia_capensis --------------------------------------------------

Choloepus_didactylus --------------------------------------------------

Myrmecophaga_tridactyla --------------------------------------------------

Macropus_eugenii --------------------------------------------------

Sarcophilus_harrisii --------------------------------------------------

Anolis_carolinensis --------------------------------------------------

Xenopus_tropicalis --------------------------------------------------

Homo_sapiens --------------------------------------------------

Gorilla_gorilla --------------------------------------------------

Otolemur_garnettii --------------------------------------------------

Mus_musculus ATGGGCCCATAAAACCTTACTGCATTTCCTCTCCAGCCTTCCACAATGGG

Rattus_norvegicus GTAGGTCCATAAAACTTTACTGCACTTCCTCTCCAGCTTCCCACAATGGG

Jaculus_jaculus --------------------------------------------------

Cavia_porcellus --------------------------------------------------

Ochotona_princeps --------------------------------------------------

Sorex_araneus --------------------------------------------------

Sus_scrofa --------------------------------------------------

Bos_taurus --------------------------------------------------

Tursiops_truncatus --------------------------------------------------

Canis_familiaris --------------------------------------------------

Loxodonta_africana --------------------------------------------------

Procavia_capensis --------------------------------------------------

Choloepus_didactylus --------------------------------------------------

Myrmecophaga_tridactyla --------------------------------------------------

Macropus_eugenii --------------------------------------------------

Sarcophilus_harrisii --------------------------------------------------

Anolis_carolinensis --------------------------------------------------

Xenopus_tropicalis --------------------------------------------------

Homo_sapiens --------------------------------------------------

Gorilla_gorilla --------------------------------------------------

Otolemur_garnettii --------------------------------------------------

Mus_musculus GTGATTTCCTGGAGCCAAGCCTAGGCCCCCCACACTGGAGGAGTGGGGAA

Rattus_norvegicus GTGATTTCCTGGAGCCAAGCCTAGGCCCCCCACCCTGGAGGAGTGGGG-A

Jaculus_jaculus --------------------------------------------------

Cavia_porcellus --------------------------------------------------

Ochotona_princeps --------------------------------------------------

Sorex_araneus --------------------------------------------------

Sus_scrofa --------------------------------------------------

Bos_taurus --------------------------------------------------

Tursiops_truncatus --------------------------------------------------

Canis_familiaris --------------------------------------------------

Loxodonta_africana --------------------------------------------------

Procavia_capensis --------------------------------------------------

Choloepus_didactylus --------------------------------------------------

Myrmecophaga_tridactyla --------------------------------------------------

Macropus_eugenii --------------------------------------------------

Sarcophilus_harrisii --------------------------------------------------

Anolis_carolinensis --------------------------------------------------

Xenopus_tropicalis --------------------------------------------------

Homo_sapiens --------------------------------------------------

Gorilla_gorilla --------------------------------------------------

Otolemur_garnettii --------------------------------------------------

Mus_musculus TCTGAAACTGGGCTAAGGGCTGCTCTACACTAGGACAGAAAGGAAACAGA

Rattus_norvegicus TCTGAAGCCGGGCTAAAGGCTGCTCTACACTAGGACAGAAAGAGAACAGA

Jaculus_jaculus --------------------------------------------------

Cavia_porcellus --------------------------------------------------

Ochotona_princeps --------------------------------------------------

Sorex_araneus --------------------------------------------------

Sus_scrofa --------------------------------------------------

Bos_taurus --------------------------------------------------

Tursiops_truncatus --------------------------------------------------

Canis_familiaris --------------------------------------------------

Loxodonta_africana --------------------------------------------------

Procavia_capensis --------------------------------------------------

Choloepus_didactylus --------------------------------------------------

Myrmecophaga_tridactyla --------------------------------------------------

Macropus_eugenii --------------------------------------------------

Sarcophilus_harrisii --------------------------------------------------

Anolis_carolinensis --------------------------------------------------

Xenopus_tropicalis --------------------------------------------------

Homo_sapiens --------------------------------------------------

Gorilla_gorilla --------------------------------------------------

Otolemur_garnettii --------------------------------------------------

Mus_musculus GTGGCTCTGGGGAGGGGCAGGGAGATTCCTTTTTATGATTATTAATGCTT

Rattus_norvegicus GTAACTCTGAGGAGGGGCAGGGAGATTTCTTTTTATGATTATTAATGTTT

Jaculus_jaculus --------------------------------------------------

Cavia_porcellus --------------------------------------------------

Ochotona_princeps --------------------------------------------------

Sorex_araneus --------------------------------------------------

Sus_scrofa --------------------------------------------------

Bos_taurus --------------------------------------------------

Tursiops_truncatus --------------------------------------------------

Canis_familiaris --------------------------------------------------

Loxodonta_africana --------------------------------------------------

Procavia_capensis --------------------------------------------------

Choloepus_didactylus --------------------------------------------------

Myrmecophaga_tridactyla --------------------------------------------------

Macropus_eugenii --------------------------------------------------

Sarcophilus_harrisii --------------------------------------------------

Anolis_carolinensis --------------------------------------------------

Xenopus_tropicalis --------------------------------------------------

Homo_sapiens --------------------------------------------------

Gorilla_gorilla --------------------------------------------------

Otolemur_garnettii --------------------------------------------------

Mus_musculus ATGCTCTTTGAATTTATTAAAGGTCTGACTTCACTGCATCCCACTGTCTA

Rattus_norvegicus ATGCTCTTTGGATTTATTGAAGGTCTGACTTCACTGCATCCCACTGTCTA

Jaculus_jaculus --------------------------------------------------

Cavia_porcellus --------------------------------------------------

Ochotona_princeps --------------------------------------------------

Sorex_araneus --------------------------------------------------

Sus_scrofa --------------------------------------------------

Bos_taurus --------------------------------------------------

Tursiops_truncatus --------------------------------------------------

Canis_familiaris --------------------------------------------------

Loxodonta_africana --------------------------------------------------

Procavia_capensis --------------------------------------------------

Choloepus_didactylus --------------------------------------------------

Myrmecophaga_tridactyla --------------------------------------------------

Macropus_eugenii --------------------------------------------------

Sarcophilus_harrisii --------------------------------------------------

Anolis_carolinensis --------------------------------------------------

Xenopus_tropicalis --------------------------------------------------

Homo_sapiens --------------------------------------------------

Gorilla_gorilla --------------------------------------------------

Otolemur_garnettii --------------------------------------------------

Mus_musculus AGCAATTGTGTCTGTCACTTTTAGAAGACTCAAGAGCCTGTCCTCATCCC

Rattus_norvegicus AACAAT--TGTCTGTCACTTTTAGAAAACTCAAGAGCC------------

Jaculus_jaculus --------------------------------------------------

Cavia_porcellus --------------------------------------------------

Ochotona_princeps --------------------------------------------------

Sorex_araneus --------------------------------------------------

Sus_scrofa --------------------------------------------------

Bos_taurus --------------------------------------------------

Tursiops_truncatus --------------------------------------------------

Canis_familiaris --------------------------------------------------

Loxodonta_africana --------------------------------------------------

Procavia_capensis --------------------------------------------------

Choloepus_didactylus --------------------------------------------------

Myrmecophaga_tridactyla --------------------------------------------------

Macropus_eugenii --------------------------------------------------

Sarcophilus_harrisii --------------------------------------------------

Anolis_carolinensis --------------------------------------------------

Xenopus_tropicalis --------------------------------------------------

Homo_sapiens --------------------------------------------------

Gorilla_gorilla --------------------------------------------------

Otolemur_garnettii --------------------------------------------------

Mus_musculus ATGCTTAGCATGTAGGAGGTCTCAGCACATGTTTGTCAAGCTGCACCCCA

Rattus_norvegicus ----------TGTAGGAGGTCTCAGCACATGTTTGTCAAGCTACA-CCCA

Jaculus_jaculus --------------------------------------------------

Cavia_porcellus --------------------------------------------------

Ochotona_princeps --------------------------------------------------

Sorex_araneus --------------------------------------------------

Sus_scrofa --------------------------------------------------

Bos_taurus --------------------------------------------------

Tursiops_truncatus --------------------------------------------------

Canis_familiaris --------------------------------------------------

Loxodonta_africana --------------------------------------------------

Procavia_capensis --------------------------------------------------

Choloepus_didactylus --------------------------------------------------

Myrmecophaga_tridactyla --------------------------------------------------

Macropus_eugenii --------------------------------------------------

Sarcophilus_harrisii --------------------------------------------------

Anolis_carolinensis --------------------------------------------------

Xenopus_tropicalis --------------------------------------------------

Homo_sapiens --------------------------------------------------

Gorilla_gorilla --------------------------------------------------

Otolemur_garnettii --------------------------------------------------

Mus_musculus TTACCACCCTTCCCTTTGCTCTGTGCTCAGGCAAGGTTAGAACACTGCTA

Rattus_norvegicus TCACCACCCTTCCCTTTGCTCTGTGCTCAGGCAAGGTTAGAACACTGCTA

Jaculus_jaculus --------------------------------------------------

Cavia_porcellus --------------------------------------------------

Ochotona_princeps --------------------------------------------------

Sorex_araneus --------------------------------------------------

Sus_scrofa --------------------------------------------------

Bos_taurus --------------------------------------------------

Tursiops_truncatus --------------------------------------------------

Canis_familiaris --------------------------------------------------

Loxodonta_africana --------------------------------------------------

Procavia_capensis --------------------------------------------------

Choloepus_didactylus --------------------------------------------------

Myrmecophaga_tridactyla --------------------------------------------------

Macropus_eugenii --------------------------------------------------

Sarcophilus_harrisii --------------------------------------------------

Anolis_carolinensis --------------------------------------------------

Xenopus_tropicalis --------------------------------------------------

Homo_sapiens --------------------------------------------------

Gorilla_gorilla --------------------------------------------------

Otolemur_garnettii --------------------------------------------------

Mus_musculus AAATCTCCCCTCAACTTGCACCCTGCAGACATGCAGCCCAGCCCCAACCA

Rattus_norvegicus AAGTCTCCCCTCAACTTGCACCCTGCAGGCATGCAGCCCAGCCTCAAGCA

Jaculus_jaculus --------------------------------------------------

Cavia_porcellus --------------------------------------------------

Ochotona_princeps --------------------------------------------------

Sorex_araneus --------------------------------------------------

Sus_scrofa --------------------------------------------------

Bos_taurus --------------------------------------------------

Tursiops_truncatus --------------------------------------------------

Canis_familiaris --------------------------------------------------

Loxodonta_africana --------------------------------------------------

Procavia_capensis --------------------------------------------------

Choloepus_didactylus --------------------------------------------------

Myrmecophaga_tridactyla --------------------------------------------------

Macropus_eugenii --------------------------------------------------

Sarcophilus_harrisii --------------------------------------------------

Anolis_carolinensis --------------------------------------------------

Xenopus_tropicalis --------------------------------------------------

Homo_sapiens --------------------------------------------------

Gorilla_gorilla --------------------------------------------------

Otolemur_garnettii --------------------------------------------------

Mus_musculus ACCAACCCTCCTCTCTCTCTCTCTCTCTCTCTCTCTCTCTCTCTCTCTCT

Rattus_norvegicus AC---CCCTCCTCTCTCTCTCTCTCTCTCTCTCTCTCTCTCTCTCTCTCT

Jaculus_jaculus --------------------------------------------------

Cavia_porcellus --------------------------------------------------

Ochotona_princeps --------------------------------------------------

Sorex_araneus --------------------------------------------------

Sus_scrofa --------------------------------------------------

Bos_taurus --------------------------------------------------

Tursiops_truncatus --------------------------------------------------

Canis_familiaris --------------------------------------------------

Loxodonta_africana --------------------------------------------------

Procavia_capensis --------------------------------------------------

Choloepus_didactylus --------------------------------------------------

Myrmecophaga_tridactyla --------------------------------------------------

Macropus_eugenii --------------------------------------------------

Sarcophilus_harrisii --------------------------------------------------

Anolis_carolinensis --------------------------------------------------

Xenopus_tropicalis --------------------------------------------------

Homo_sapiens --------------------------------------------------

Gorilla_gorilla --------------------------------------------------

Otolemur_garnettii --------------------------------------------------

Mus_musculus CTCT---------------------CTCTCTCTCTCTCTC----------

Rattus_norvegicus CTCTGTGCACACACACACACACACACACACACACACACACAGAGAGAGAG

Jaculus_jaculus --------------------------------------------------

Cavia_porcellus --------------------------------------------------

Ochotona_princeps --------------------------------------------------

Sorex_araneus --------------------------------------------------

Sus_scrofa --------------------------------------------------

Bos_taurus --------------------------------------------------

Tursiops_truncatus --------------------------------------------------

Canis_familiaris --------------------------------------------------

Loxodonta_africana --------------------------------------------------

Procavia_capensis --------------------------------------------------

Choloepus_didactylus --------------------------------------------------

Myrmecophaga_tridactyla --------------------------------------------------

Macropus_eugenii --------------------------------------------------

Sarcophilus_harrisii --------------------------------------------------

Anolis_carolinensis --------------------------------------------------

Xenopus_tropicalis --------------------------------------------------

Homo_sapiens --------------------------------------------------

Gorilla_gorilla --------------------------------------------------

Otolemur_garnettii --------------------------------------------------

Mus_musculus -----------------------------------AGAGACAGCCAGAGA

Rattus_norvegicus AGAGAGAGAGAGAGAGAGAGAGAGGGGAGAGAGAGAGAGAGAGAGAGAGA

Jaculus_jaculus --------------------------------------------------

Cavia_porcellus --------------------------------------------------

Ochotona_princeps --------------------------------------------------

Sorex_araneus --------------------------------------------------

Sus_scrofa --------------------------------------------------

Bos_taurus --------------------------------------------------

Tursiops_truncatus --------------------------------------------------

Canis_familiaris --------------------------------------------------

Loxodonta_africana --------------------------------------------------

Procavia_capensis --------------------------------------------------

Choloepus_didactylus --------------------------------------------------

Myrmecophaga_tridactyla --------------------------------------------------

Macropus_eugenii --------------------------------------------------

Sarcophilus_harrisii --------------------------------------------------

Anolis_carolinensis --------------------------------------------------

Xenopus_tropicalis --------------------------------------------------

Homo_sapiens --------------------------------------------------

Gorilla_gorilla --------------------------------------------------

Otolemur_garnettii --------------------------------------------------

Mus_musculus GAGAGAGAGAGAGAGAAATAAAATGTGTGGTTTTCTTCAGGGAGTTATTT

Rattus_norvegicus GAGAGAGAGAGAGAGAGAGAGAATGTGTGGTTTTCTTCAGGGAGTTATTT

Jaculus_jaculus --------------------------------------------------

Cavia_porcellus --------------------------------------------------

Ochotona_princeps --------------------------------------------------

Sorex_araneus --------------------------------------------------

Sus_scrofa --------------------------------------------------

Bos_taurus --------------------------------------------------

Tursiops_truncatus --------------------------------------------------

Canis_familiaris --------------------------------------------------

Loxodonta_africana --------------------------------------------------

Procavia_capensis --------------------------------------------------

Choloepus_didactylus --------------------------------------------------

Myrmecophaga_tridactyla --------------------------------------------------

Macropus_eugenii --------------------------------------------------

Sarcophilus_harrisii --------------------------------------------------

Anolis_carolinensis --------------------------------------------------

Xenopus_tropicalis --------------------------------------------------

Homo_sapiens --------------------------------------------------

Gorilla_gorilla --------------------------------------------------

Otolemur_garnettii --------------------------------------------------

Mus_musculus TAATTTTTTCTACATGCTGCGTAAAACTATTGGGTGAGCATTGAAGCATG

Rattus_norvegicus TAATTTTTTCTACATGCTGCGTAAAACTATTGGGTGAGCATTGAAGCATG

Jaculus_jaculus --------------------------------------------------

Cavia_porcellus --------------------------------------------------

Ochotona_princeps --------------------------------------------------

Sorex_araneus --------------------------------------------------

Sus_scrofa --------------------------------------------------

Bos_taurus --------------------------------------------------

Tursiops_truncatus --------------------------------------------------

Canis_familiaris --------------------------------------------------

Loxodonta_africana --------------------------------------------------

Procavia_capensis --------------------------------------------------

Choloepus_didactylus --------------------------------------------------

Myrmecophaga_tridactyla --------------------------------------------------

Macropus_eugenii --------------------------------------------------

Sarcophilus_harrisii --------------------------------------------------

Anolis_carolinensis --------------------------------------------------

Xenopus_tropicalis --------------------------------------------------

Homo_sapiens --------------------------------------------------

Gorilla_gorilla --------------------------------------------------

Otolemur_garnettii --------------------------------------------------

Mus_musculus AAGAAGCACTGGCAGCTCAGATGGGTAACAGTTAAATAAGGAAGA-GGTT

Rattus_norvegicus AAGAAGCACTGGCGGCTCGGATGGGTAGCAGTTAAATAAGGAAGAGGGTT

Jaculus_jaculus --------------------------------------------------

Cavia_porcellus --------------------------------------------------

Ochotona_princeps -------------------------------TTAAAAAAAAAAAAAAGCT

Sorex_araneus --------------------------------------------------

Sus_scrofa --------------------------------------------------

Bos_taurus --------------------------------------------------

Tursiops_truncatus --------------------------------------------------

Canis_familiaris --------------------------------------------------

Loxodonta_africana --------------------------------------------------

Procavia_capensis --------------------------------------------------

Choloepus_didactylus --------------------------------------------------

Myrmecophaga_tridactyla --------------------------------------------------

Macropus_eugenii --------------------------------------------------

Sarcophilus_harrisii --------------------------------------------------

Anolis_carolinensis --------------------------------------------------

Xenopus_tropicalis --------------------------------------------------

Homo_sapiens --------------------------------------------------

Gorilla_gorilla --------------------------------------------------

Otolemur_garnettii --------------------------------------------------

Mus_musculus ATGGTGCCCAAACTCACTGGTTCCAGCCTCCCAGACTTCAGGGGGACTCA

Rattus_norvegicus ATGGCGCCCACACTCACCGGTTCCAGCCTCCCAGACTTCAGGAGGACCTG

Jaculus_jaculus --------------------------------------------------

Cavia_porcellus --------------------------------------------------

Ochotona_princeps TTAGAAACTTCTAGGAACAAGGAGAAAAAGAGAGATGAAAGTGGCCTTCT

Sorex_araneus --------------------------------------------------

Sus_scrofa --------------------------------------------------

Bos_taurus --------------------------------------------------

Tursiops_truncatus --------------------------------------------------

Canis_familiaris --------------------------------------------------

Loxodonta_africana --------------------------------------------------

Procavia_capensis --------------------------------------------------

Choloepus_didactylus --------------------------------------------------

Myrmecophaga_tridactyla --------------------------------------------------

Macropus_eugenii --------------------------------------------------

Sarcophilus_harrisii --------------------------------------------------

Anolis_carolinensis --------------------------------------------------

Xenopus_tropicalis --------------------------------------------------

Homo_sapiens --------------------------------------------------

Gorilla_gorilla --------------------------------------------------

Otolemur_garnettii --------------------------------------------------

Mus_musculus TGCTTCTTCCCCAGGGGG-GCACACCAGGGCTTGATGGGAAGGGAATAAG

Rattus_norvegicus TGCTTCCTCCCCAGGGGGAACACACCAGGGCTTGATGGGGAGGGAATAGG

Jaculus_jaculus --------------------------------------------------

Cavia_porcellus --------------------------------------------------

Ochotona_princeps TGCTGATTTCTCGGAGAGGCGAGCAGCCGGAGGGATCAGGGGAGGGCTTG

Sorex_araneus --------------------------------------------------

Sus_scrofa --------------------------------------------------

Bos_taurus --------------------------------------------------

Tursiops_truncatus --------------------------------------------------

Canis_familiaris --------------------------------------------------

Loxodonta_africana --------------------------------------------------

Procavia_capensis --------------------------------------------------

Choloepus_didactylus --------------------------------------------------

Myrmecophaga_tridactyla --------------------------------------------------

Macropus_eugenii --------------------------------------------------

Sarcophilus_harrisii --------------------------------------------------

Anolis_carolinensis --------------------------------------------------

Xenopus_tropicalis --------------------------------------------------

Homo_sapiens --------------------------------------------------

Gorilla_gorilla --------------------------------------------------

Otolemur_garnettii --------------------------------------------------

Mus_musculus GGTGATATTGATCAGACCAGACTGGCAAAGTTCATCAGCACTCTATGAGT

Rattus_norvegicus AGCGATATTGATCAG-----------------------------------

Jaculus_jaculus --------------------------------------------------

Cavia_porcellus --------------------------------------------------

Ochotona_princeps AAAGCCG-------------------------------------------

Sorex_araneus --------------------------------------------------

Sus_scrofa --------------------------------------------------

Bos_taurus --------------------------------------------------

Tursiops_truncatus --------------------------------------------------

Canis_familiaris --------------------------------------------------

Loxodonta_africana --------------------------------------------------

Procavia_capensis --------------------------------------------------

Choloepus_didactylus --------------------------------------------------

Myrmecophaga_tridactyla --------------------------------------------------

Macropus_eugenii --------------------------------------------------

Sarcophilus_harrisii --------------------------------------------------

Anolis_carolinensis --------------------------------------------------

Xenopus_tropicalis --------------------------------------------------

Homo_sapiens -----

Gorilla_gorilla -----

Otolemur_garnettii -----

Mus_musculus TTTAT

Rattus_norvegicus -----

Jaculus_jaculus -----

Cavia_porcellus -----

Ochotona_princeps -----

Sorex_araneus -----

Sus_scrofa -----

Bos_taurus -----

Tursiops_truncatus -----

Canis_familiaris -----

Loxodonta_africana -----

Procavia_capensis -----

Choloepus_didactylus -----

Myrmecophaga_tridactyla -----

Macropus_eugenii -----

Sarcophilus_harrisii -----

Anolis_carolinensis -----

Xenopus_tropicalis -----

**Supplement S3B**

RNAz output for the alignment of miR-615 precursors across Eutheria.

Structural conservation index (SCI) is a measure of structural conservation and is dependent on mean pairwise identity and number of sequences in the alignment. The SCI reported here is 0.87 with a mean pairwise identity of 92.62%, suggesting that the fold is conserved.

The mean z-score for the fold is -3.78, indicating a stable structure that is unlikely to have arisen by chance.

RNA-class probability is > 0.9 with an associated false positive rate of ~1%.


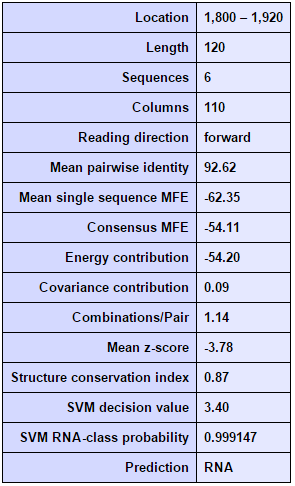


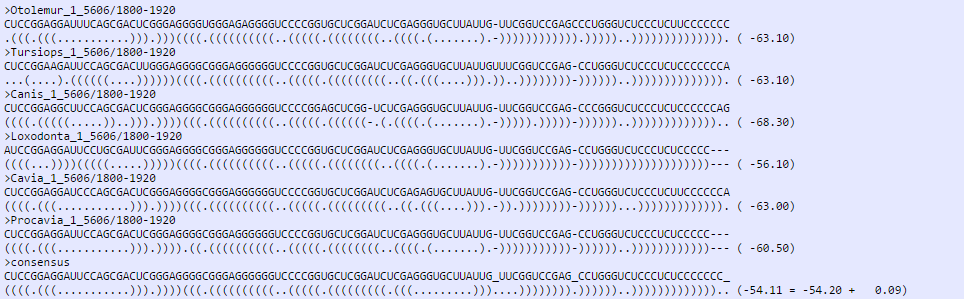


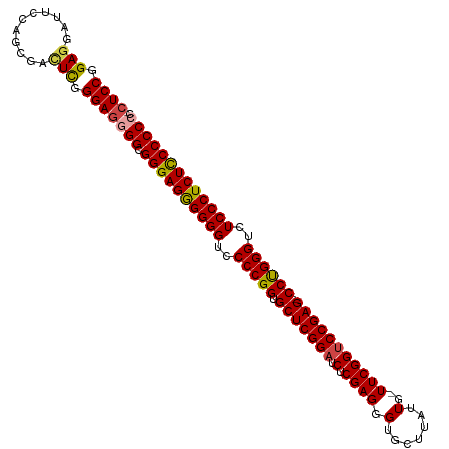


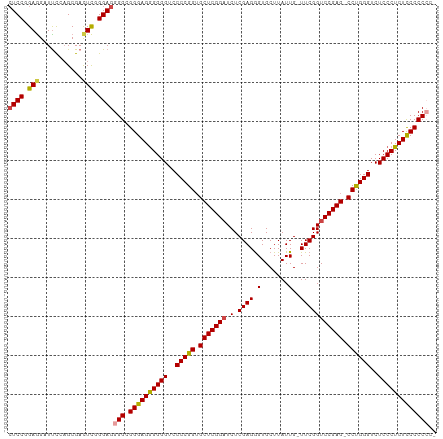

Supplement: Supplementary file 3 — 10.1186/s13227-015-0027-1 (A) Alignment of tetrapod Hoxc5 loci including exonic regions. (B) RNAz analysis for eutherian pre-miR-615. [file 13227_2015_27_MOESM3_ESM.docx]
