# Supplementary material for: The Hox cluster microRNA miR-615: a case study of intronic microRNA evolution
Source: EvoDevo. 2015 Oct 7;6:31. doi: 10.1186/s13227-015-0027-1 (PMC4597612; doi:10.1186/s13227-015-0027-1)
Supplement: Supplementary file 4 — 10.1186/s13227-015-0027-1 Predicted secondary structures and minimum free energies for the region corresponding to mir-615 across therian mammals. [file 13227_2015_27_MOESM4_ESM.docx]

**Supplement S4**

| Cell line/Tissue | Description | Sex | RNAseq data available? |
| --- | --- | --- | --- |
| A549 | Epithelial cell line, lung carcinoma | Male | Yes |
| AG04450 | Foetal lung fibroblast | Male | Yes |
| BJ | Skin fibroblast | Male | Yes |
| CD14+ | Monocytes | Female | Yes |
| B cells CD20+ | B cells | Female | Yes |
| CD34+ | Haematopoietic progenitor cells - mobilized | Male | Yes |
| GM12878 | Lymphoblastoid | Female | Yes |
| H1-hESC | Embryonic stem cell | Male | Yes |
| H1-neurons | Neurons derived from H1 ESCs | Male | No |
| HAoAF | Aortic adventitial fibroblasts | Both | Yes |
| HAoEC | Aortic endothelial cells | Both | Yes |
| HCH | Undifferentiated chondrocytes | Female | Yes |
| HeLa-S3 | Cervical carcinoma | Female | Yes |
| HepG2 | Liver carcinoma | Male | Yes |
| HFDPC | Follicle dermal papilla cells | Female | Yes |
| HMEpC | Mammary epithelial cells | Female | Yes |
| hMSC-AT | Undifferentiated mesenchymal stem cells from abdomen adipose tissue | Female | Yes |
| hMSC-BM | Undifferentiated mesenchymal stem cells from femoral head | Female | Yes |
| hMSC-UC | Undifferentiated mesenchymal stem cells from matrix (umbilical cord) | Female | Yes |
| HOB | Undifferentiated osteoblasts | Female | Yes |
| HPC-PL | Undifferentiated pericytes | Female | Yes |
| HPIEpC | Placental epithelial cells amniotic membrane | Female | Yes |
| HSaVEC | Saphenous vein endothelial cells | Male | Yes |
| HVMF | Villous mesenchymal fibroblasts | Male | Yes |
| HWP | Undifferentiated white preadipocytes | Male | Yes |
| IMR90 | Foetal lung fibroblast | Female | Yes |
| K562 | Chronic myelogenous leukaemia | Female | Yes |
| MCF-7 | Mammary gland, adenocarcinoma | Female | Yes |
| NHDF | Dermal fibroblasts | Female | Yes |
| NHEK | Epidermal keratinocytes | Unknown | Yes |
| NHEM.f_M2 | Epidermal melanocytes | Male | Yes |
| NHEM_M2 | Epidermal melanocytes | Both | Yes |
| Prostate | Prostate tissue | Male | No |
| SkMC | Skeletal striated muscle cells | Both | Yes |
| SK-N-SH | Neuroblastoma | Female | Yes |
| SK-N-SH_RA | Neuroblastoma cell line, treated with retinoic acid | Female | Yes |

Cell lines available through the ENCODE Consortium (2003-2012) for which small RNA sequencing data is available.

Summary of data representing small RNA and mRNA sequencing in ENCODE cell lines

| Cell line/Tissue | miR-615 expression | *HoxC5* expression |
| --- | --- | --- |
| A549 | + | - |
| AG04450 | Low | - |
| BJ | Low | - |
| CD14+ | - | - |
| B cells CD20+ | - | - |
| CD34+ | - | - |
| GM12878 | + | - |
| H1-hESC | - | - |
| H1-neurons | - | (No data available) |
| HAoAF | + | Low |
| HAoEC | Low | - |
| HCH | + | + |
| HeLa-S3 | + | - |
| HepG2 | Low | - |
| HFDPC | + | - |
| HMEpC | + | - |
| hMSC-AT | + | + |
| hMSC-BM | + | + |
| hMSC-UC | + | + |
| HOB | + | + |
| HPC-PL | + | Low |
| HPIEpC | + | - |
| HSaVEC | + | - |
| HVMF | + | Low |
| HWP | + | - |
| IMR90 | + | - |
| K562 | + | - |
| MCF-7 | + | - |
| NHDF | + | + |
| NHEK | + | Low |
| NHEM.f_M2 | + | Low |
| NHEM_M2 | + | - |
| Prostate | + | (No data available) |
| SkMC | + | + |
| SK-N-SH | + | + |
| SK-N-SH_RA | + | + |
