# Supplementary figures and images for: The Hox cluster microRNA miR-615: a case study of intronic microRNA evolution
Source: EvoDevo. 2015 Oct 7;6:31. doi: 10.1186/s13227-015-0027-1 (PMC4597612; doi:10.1186/s13227-015-0027-1)

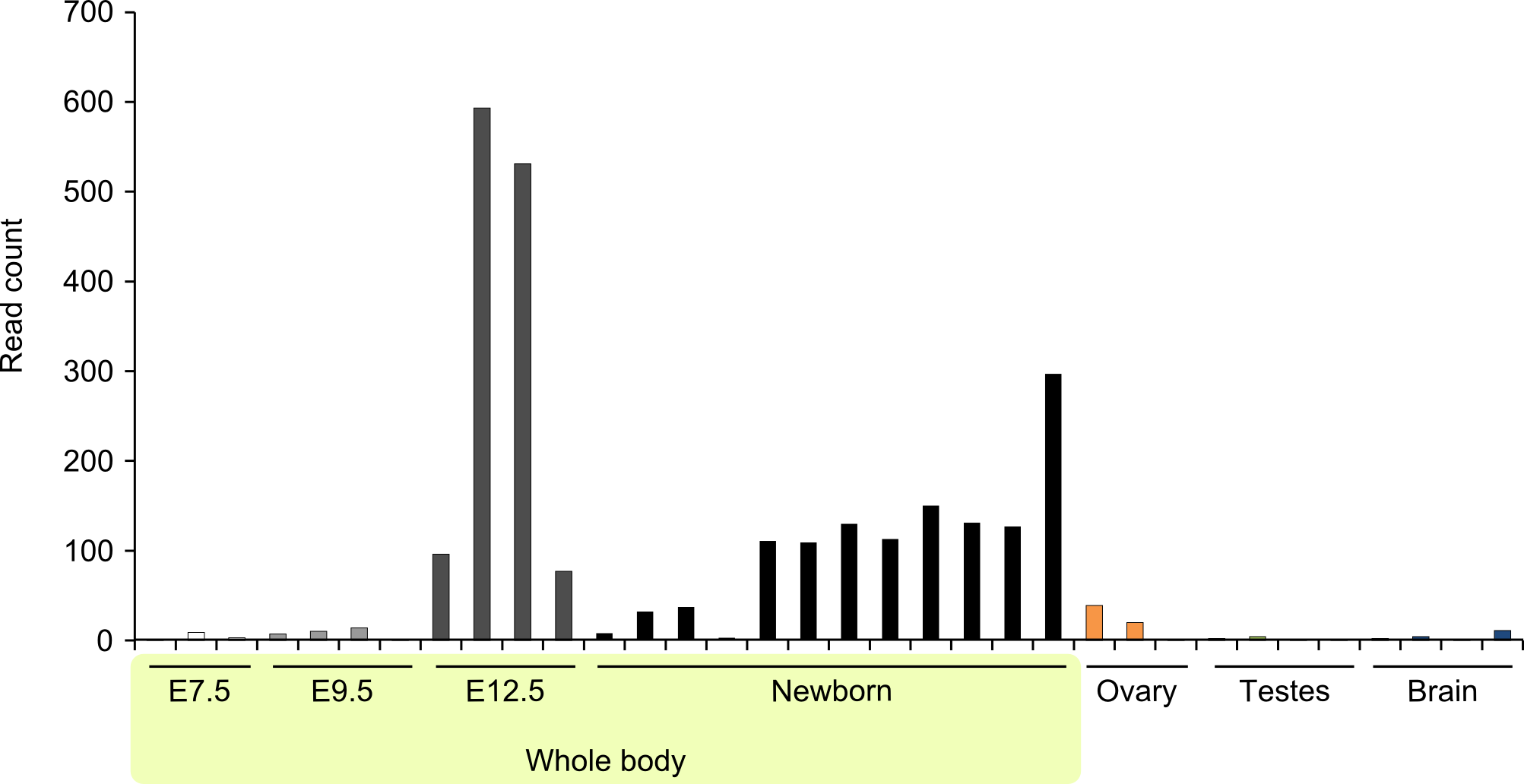

Supplement: Supplementary file 6 — 10.1186/s13227-015-0027-1 (A) Read counts for miR-615 mature products in the GM12878 and K562 cell lines, as generated by the miRDeep2 quantifier.pl script. Raw small RNA sequencing data were obtained from the ENCODE Consortium. (B) Data from Zhang et al. (2013) showing normalized tissue-specific read counts for the dominant product of miR-615 (miR-615-3p). Sertoli cells, spermatocytes and spermatids were only sampled in mouse [27]. (C) Read counts for both mature products of miR-615 in pooled ovarian sample from six pregnant and six non-pregnant goats. The dataset used for this analysis was obtained from Zhang et al. (2013). [file 13227_2015_27_MOESM6_ESM.png]

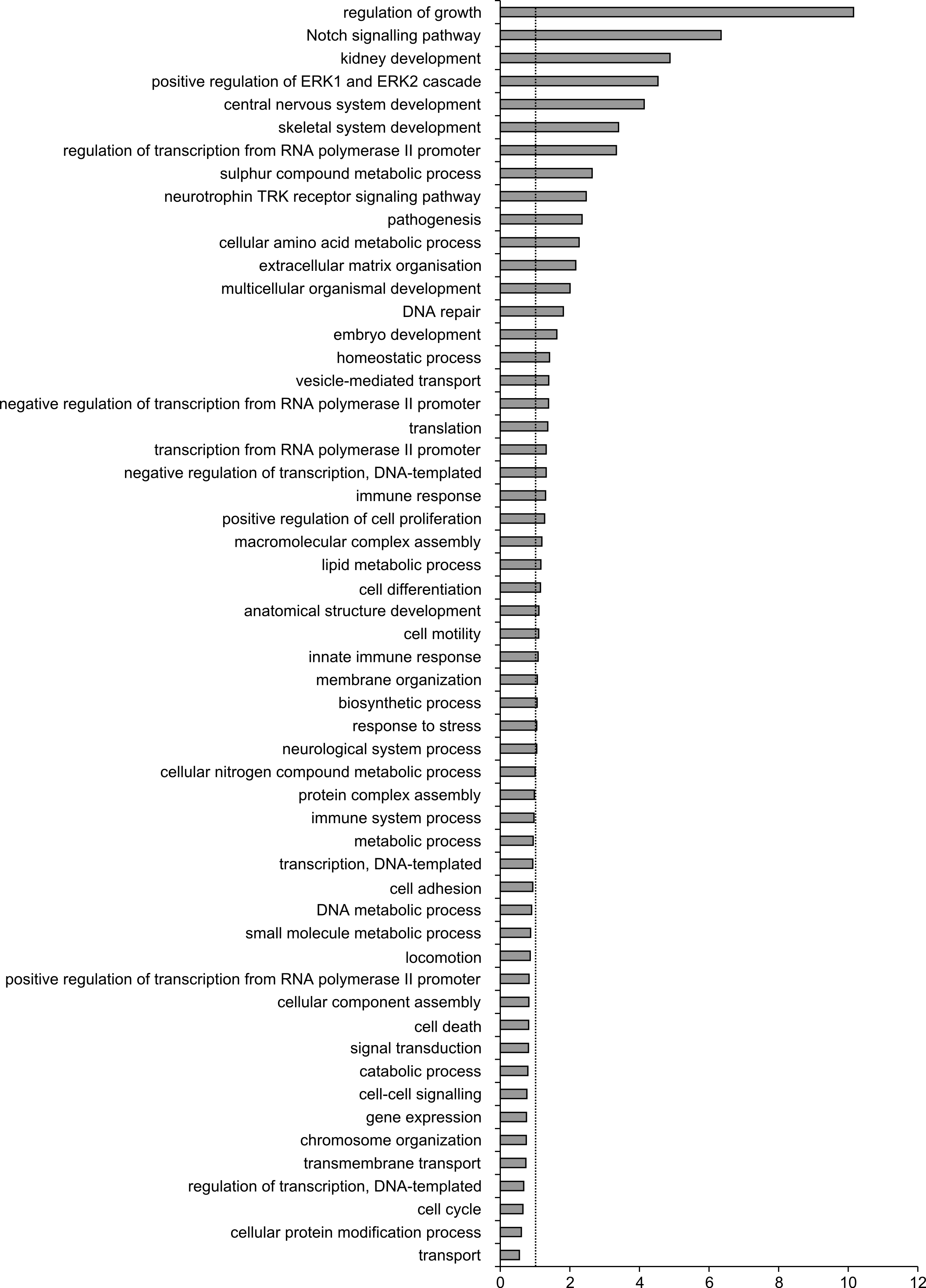

Supplement: Supplementary file 10 — 10.1186/s13227-015-0027-1 GO terms identified in the miR-615-3p target prediction dataset. The normalised frequency of each term is plotted relative to its frequency in a set of all human cDNAs. The dotted line indicates equal frequency of expression in both datasets. [file 13227_2015_27_MOESM10_ESM.png]
