# Supplementary material for: The Hox cluster microRNA miR-615: a case study of intronic microRNA evolution
Source: EvoDevo. 2015 Oct 7;6:31. doi: 10.1186/s13227-015-0027-1 (PMC4597612; doi:10.1186/s13227-015-0027-1)
Supplement: Supplementary file 7 — 10.1186/s13227-015-0027-1 miR-615-3p read counts in mouse small RNA libraries as reported in the dataset of Chiang et al. (2010), refer to [21]. Data were obtained through miRBase. [file 13227_2015_27_MOESM7_ESM.docx]

**Supplement S7**

RPKM values for *HoxC* genes in a K562 transcriptome assembly. Raw reads were obtained through the ENCODE Consortium.

| **HoxC4** | **HoxC5** | **HoxC6** | **HoxC8** | **HoxC9** | **HoxC10** | **HoxC11** | **HoxC12** | **HoxC13** |
| --- | --- | --- | --- | --- | --- | --- | --- | --- |
| 1.08 | 0 | 0.73 | 0.72 | 4.19 | 0 | 0 | 0 | 0 |
