# Supplementary material for: The Hox cluster microRNA miR-615: a case study of intronic microRNA evolution
Source: EvoDevo. 2015 Oct 7;6:31. doi: 10.1186/s13227-015-0027-1 (PMC4597612; doi:10.1186/s13227-015-0027-1)
Supplement: Supplementary file 8 — 10.1186/s13227-015-0027-1 RPKM values for HOXC genes in a K562 transcriptome assembly. Raw reads were obtained through the ENCODE Consortium. [file 13227_2015_27_MOESM8_ESM.docx]

**Supplement S8**

RPKM values as determined by RSEM on ovarian transcriptome assemblies for *M. musculus* and *H. sapiens*. Raw data used was obtained from the ENCODE Consortium.

| **Species** | **Description** | *Hoxc4* | *Hoxc5* | *Hoxc6* | *Hoxc8* | *Hoxc9* | *Hoxc10* | *Hoxc11* | *Hoxc12* | *Hoxc13* |
| --- | --- | --- | --- | --- | --- | --- | --- | --- | --- | --- |
| *Mus musculus* | Ovary, age = 10 weeks. | 13.8 | 5.05 | 78.56 | 32.38 | 5.1 | 14.9 | 0 | 0 | 0 |
| *Homo sapiens* | Ovary, age = 47 years | 8.78 | 131.88 | 19.01 | 12.12 | 7.06 | 7.09 | 0 | 0 | 0 |
