# Supplementary material for: The Hox cluster microRNA miR-615: a case study of intronic microRNA evolution
Source: EvoDevo. 2015 Oct 7;6:31. doi: 10.1186/s13227-015-0027-1 (PMC4597612; doi:10.1186/s13227-015-0027-1)
Supplement: Supplementary file 9 — 10.1186/s13227-015-0027-1 PITA prediction targets for miR-615-3p. [file 13227_2015_27_MOESM9_ESM.docx]

**Supplement S9**

PITA prediction targets for miR-615-3p

| **Gene** | **Function** | **Kidney** | **Testis** | **Ovary** | **Cerebellum** |
| --- | --- | --- | --- | --- | --- |
| ACACB | Regulation of fatty acid oxidation | 20 | 12 | 21 | 5.12 |
| AES | Corepressor for NF-kappaB | 178 | 98 | 165 | 39.03 |
| AGO3 | RISC complex component | 4 | 12 | 5 | 19.19 |
| AMER1 | Inhibition of Wnt signalling through promoting β-catenin degradation | 2 | 1 | 6 | 4.43 |
| ARHGAP35 | Regulation over formation of the cytokinetic furrow during cell division | 20 | 59 | 17 | 33.51 |
| BATF2 | Possible role in regulation of Wnt signalling pathway | 4 | 1 | 3 | 0.08 |
| BIN2 | Promotes cell motility and migration | 2 | 12 | 2 | 0.48 |
| BLOC1S3 | Component of the BLOC-1 complex, required for normal biogenesis of lysosome-related organelles | 3 | 5 | 5 | 1.07 |
| C12orf65 | Mitochondrial matrix protein, possibly involved in mitochondrial translation | 14 | 16 | 19 | 6.37 |
| CACNA1A | Component of voltage-gated calcium channel, predominantly neuronal expression | 0 | 3 | 1 | 28.03 |
| CAMK2D | Serine/threonine kinase important in cardiac and skeletal muscle function | 16 | 24 | 27 | 41.93 |
| CARNS1 | Synthesis of carnosine and homocarnosine | 1 | 1 | 1 | 0.19 |
| CD14 | Cell surface antigen involved in innate immune response to bacterial lipopolysaccharide | 20 | 31 | 15 | 7.53 |
| CENPP | Kinetochore component | 1 | 1 | 1 | 1.64 |
| CES1 | Triglyceride and cholesterol metabolism | 3 | 8 | 20 | 0.33 |
| CFC1B | Unknown | 0 | 0 | 0 | 0 |
| CHST12 | Catalyzes the transfer of sulphate to position 4 on N-acetylgalactosamine | 5 | 4 | 3 | 3.57 |
| CHST6 | Catalyzes the transfer of sulphate to position 6 on N-acetylgalactosamine | 0 | 1 | 0 | 1.37 |
| CIC | Transcriptional repressor, possibly involved in development of the central nervous system | 15 | 39 | 28 | 10.15 |
| CYSLTR2 | Receptor for cysteinyl leukotrienes | 1 | 1 | 1 | 0.52 |
| DCHS1 | Calcium-dependent cell adhesion protein, may be involved in neurogenesis | 3 | 4 | 8 | 26.4 |
| DCLRE1B | 5'-3' exonuclease, involved in telomere protection and maintenance | 2 | 3 | 3 | 3.6 |
| DLL3 | Notch signalling pathway, potential roles in axial patterning and inhibition of neurogenesis | 0 | 1 | 0 | 7.13 |
| EIF3B | Required for initiation of protein synthesis | 39 | 73 | 56 | 24.68 |
| ENSA | Regulation of ATP-sensitive potassium channels | 89 | 40 | 71 | 43.31 |
| EPN1 | Regulation of receptor-mediated endocytosis, required for activation of Notch signalling in mammals | 38 | 32 | 22 | 10.41 |
| FAH | Amino acid degradation | 56 | 19 | 25 | 4.18 |
| FAM134B | Required for long-term survival of nociceptive and autonomic ganglion neurons | 38 | 42 | 3 | 10.6 |
| FURIN | Ubiquitous endoprotease activity within constitutive secretory pathways | 25 | 8 | 10 | 4.93 |
| FXYD3 | Regulator of Na-K ATPase activity | 9 | 0 | 1 | 0.32 |
| GAB2 | Adapter protein acting downstream of several membrane receptors | 5 | 6 | 19 | 7.07 |
| GCN1L1 | Translational activator | 15 | 23 | 19 | 31.32 |
| GDAP2 | Unknown | 6 | 12 | 4 | 18.22 |
| HAPLN4 (BRAL2) | Synaptic stabilization, involved in central nervous system development | 0 | 0 | 0 | 0.63 |
| HSD17B12 | Fatty acid elongation | 72 | 43 | 48 | 37.15 |
| IQGAP3 | Rho family GTPase, promotes axon and neurite outgrowth in brain | 0 | 4 | 0 | 2.67 |
| KIF1A | Motor protein for anterograde axonal transport of synaptic vesicle precursors | 0 | 14 | 2 | 90.37 |
| LMNTD2 | Unknown | 3.7 | 12.3 | 0 | 0.06 |
| LPIN3 | Regulates fatty acid metabolism | 11 | 4 | 12 | 0.15 |
| LSP1 | Cytoskeletal organisation and motility in neutrophils | 5 | 17 | 5 | 0.34 |
| MADCAM1 | Immunoglobulin superfamily adhesion molecule expressed by lymphocytes | 0 | 1 | 0 | 0.77 |
| MED22 | Mediator complex component, involved in transcriptional regulation of Pol II-dependent genes | 9 | 7 | 13 | 8.81 |
| NARS | Asparagine--tRNA ligase | 103 | 43 | 48 | 33.5 |
| NDN | Growth suppressor, promotes cell cycle arrest particularly in postmitotic neurons in the brain | 10 | 21 | 64 | 27.84 |
| NFRKB | Component of the INO80 complex involved in chromatin remodelling, transcriptional regulation and DNA repair | 9 | 23 | 20 | 9.86 |
| NODAL | Morphogen required for mesoderm induction and axial patterning | 0 | 1 | 0 | 0.43 |
| NPEPL1 | Unknown | 24 | 25 | 19 | 5.06 |
| ORMDL3 | Endoplasmic reticulum transmembrane protein, mediates sphingolipid homeostasis | 39 | 37 | 36 | 18.63 |
| PAX6 | Transcription factor, involved in development of the eye, nose, forebrain and pancreas | 1 | 2 | 0 | 63.86 |
| PBX3 | Transcriptional activator | 7 | 13 | 76 | 53.12 |
| PES1 | Ribosomal RNA processing | 25 | 29 | 30 | 12.91 |
| PHACTR3 (SCAPININ) | Regulation of the actin cytoskeleton involved in cell motility | 2 | 1 | 5 | 8.54 |
| PIF1 | Inhibition of telomerase activity | 1 | 3 | 0 | 2.23 |
| PLA2G2C | Unknown, possibly fertilization. Expressed in mouse testis. | 0 | 0 | 0 | 0 |
| PRAME | Dominant repressor of retinoic acid signalling | 1 | 78 | 3 | 0.04 |
| PRG2 (LPPR3) | Integral membrane protein, modulates bioactive lipid phosphates in the context of cell migration, neurite retraction and mitogenesis | 0 | 1 | 0 | 0.16 |
| PTP4A3 | Promotes cell proliferation | 9 | 12 | 5 | 13.33 |
| RBFA | Ribosomal RNA processing | 12 | 11 | 11 | 26.72 |
| RSL1D1 (CSIG) | Regulation of cell cycle, cell senescence and apoptosis | 59 | 53 | 249 | 24.01 |
| SESN2 | Modulator of peroxide signalling, antioxidant defence, regulation of cell growth | 15 | 8 | 13 | 1.36 |
| SKA3 | Component of Ska complex which mediates kinetochore-microtubule interactions during mitosis | 0 | 8 | 0 | 5.26 |
| SLC2A11 (GLUT11) | Glucose transporter | 25 | 8 | 13 | 13.95 |
| SPECC1 | Unknown, highly expressed in testicular tissue | 6 | 35 | 23 | 28.82 |
| STRA6 | Receptor for retinol binding protein, required for retinol transport to specific sites e.g. eye | 13 | 7 | 1 | 5.53 |
| STRBP | Microtubule-associated RNA binding protein, highly expressed in testis and brain and possibly important to development of these structures | 12 | 49 | 4 | 34.87 |
| STX16-NPEPL1 | Naturally occuring read-through transcript between STX16 and NPEPL1, unlikely to be functional | 4 | 2 | 3 | 5.21 |
| TMEM161A | Unknown | 12 | 6 | 12 | 4.52 |
| TRAPPC4 | Component of TRAPP complex involved in vesicle trafficking | 62 | 57 | 39 | 13.96 |
| TRAPPC5 | Component of TRAPP complex involved in vesicle trafficking | 27 | 52 | 16 | 19.42 |
| TRIM31 | TRIM family member, possibly involved in cell compartment specification | 0 | 2 | 0 | 0.02 |
| UBA1 | Ubiquitin conjugation, required for ubiquitylation-dependent signalling of double-stranded breaks and replication stress in cells | 83 | 84 | 80 | 47.62 |
| YY1AP1 | Coactivator for transcriptional regulator YY1 | 21 | 71 | 42 | 92.15 |
